# A Pan‐Methylome Framework for Population‐Scale Bacterial Epigenomics

**DOI:** 10.1002/advs.76559

**Published:** 2026-07-13

**Authors:** Bin Ma, Huimin Gong, Ran Zhuo, Zaixiao Rao, Yanxi Wan, Mingyang Wang, Zahra Zeinalzadeh, Yuan Gao, Aohan Guan, Xinrun Li, Wanting Chen, Xiliang Wang, Yun Wan, Sihua Zhang, Yuncai Xiao, Qi Huang, Paul R. Langford, Xiangru Wang, Rui Zhou, Rui Luo, Hongbo Zhou, Chengliang Zhu, Hui Jin

**Affiliations:** ^1^ State Key Laboratory of Agricultural Microbiology College of Veterinary Medicine Huazhong Agricultural University Wuhan China; ^2^ Key Laboratory of Preventive Veterinary Medicine in Hubei Province The Cooperative Innovation Center for Sustainable Pig Production Wuhan China; ^3^ Hubei Jiangxia Laboratory Wuhan China; ^4^ Department of Biological Sciences Smith College Northampton MA USA; ^5^ Wuhan Animal Disease Control Center Wuhan Hubei China; ^6^ Section of Paediatric Infectious Disease Imperial College London South Kensington London UK; ^7^ Department of Clinical Laboratory Renmin Hospital of Wuhan University Wuhan China

**Keywords:** biology, computational biology, dna methylation, epigenetics, epigenomics, epigenome, genetic code, genetics, quantitative trait locus

## Abstract

Epigenetic modifications furnish a hidden regulatory layer that shapes cellular fate and evolutionary potential without rewriting the genetic code. In eukaryotes, methylome maps have profoundly transformed our understanding of development, disease, and lineage diversification. In bacteria, which represent the most abundant and ecologically versatile forms of life, the epigenome remains a largely uncharted dimension. To enable population‐scale comparison of bacterial methylomes, we develop a principled strategy to prioritize methylation systems that are enzymatically encoded, broadly conserved and phylogenetically informative, thereby defining a stable substrate for quantitative comparative analysis. We reconstruct the first population‐scale bacterial methylation‐informed phylogenies that broadly recapitulate sequence‐based relationships while resolving epi‐phylogroups associated with GC content and environmental stress resilience. We introduce a three‐metric quantitative framework (MPK, MR, and MFR) that converts site‐level methylation calls into standardized, cross‐sample comparable quantitative traits, enabling robust identification of highly methylated core genes from as few as 30 *Escherichia coli* strains. Finally, co‐methylation network analysis identifies virulence‐enriched modules and coordinated methylation of horizontally acquired virulence loci, providing the first evidence that horizontally acquired virulence loci are epigenetically assimilated into host regulatory circuitry. Together, this framework enables locus‐resolved, population‐scale integration of bacterial methylation data to interrogate epigenetic contributions to evolution, fitness and pathogenicity.

## Graphical abstract

A scalable quantitative framework unlocks population‐level comparative epigenomics in bacteria. By transforming site‐level data into standardized traits, this approach reconstructs methylation‐informed phylogenies and defines the core epigenome. Co‐methylation networks uncover the epigenetic assimilation of horizontally acquired virulence loci, revealing a hidden regulatory dimension that drives bacterial evolution, environmental resilience, and pathogenicity.

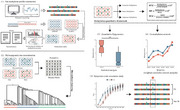

## 1 Introduction

Unlike DNA sequence variation exemplified by mutations, rearrangements, or insertions, epigenetic modifications such as DNA methylation regulate gene expression and chromatin architecture without altering the underlying nucleotide sequence [1, 2]. In a landmark study, Haghani et al. used conserved CpG methylation profiles from 348 mammalian species to construct phyloepigenetic trees that accurately recapitulate and resolve species‐level evolutionary relationships [3]. In humans and other eukaryotes, methylome profiling has transformed our understanding of development, disease, and evolution by charting precise, heritable patterns of DNA modification [3, 4, 5]. In contrast, the bacterial methylome is still viewed through a narrow lens. Most studies have focused on model species/strains and single‐genome contexts [6, 7, 8], leaving the population‐level diversity, organization, and functional significance of bacterial methylomes remain largely unexplored.

Recent advances in pan‐genomics have expanded our understanding of intraspecies genetic diversity by defining the collective gene repertoire of a species across multiple individuals [9]. This framework has since inspired the emerging concept of pan‐epigenomics, which systematically characterizes population‐wide epigenetic variation beyond single reference genomes. Tisza et al. recently provided a valuable exploration of population‐scale motif discovery in the *Bacteroides fragilis* group, where a large‐scale pangenomic analysis across 268 clinical isolates identified hundreds of DNA methylation motifs using Nanopore sequencing [10]. Nevertheless, no high‐throughput quantification of methylation levels at specific genomic loci was performed, nor were methylation‐based phylogenetic trees or dynamic comparisons of methylation patterns across strains systematically explored. These limitations leave the functional and evolutionary implications of the observed epigenomic diversity largely unaddressed. However, no analogous framework exists for the prokaryotic epigenome, and fundamental questions remain. For instance, do methylation patterns exhibit structured variability across bacterial populations? Additionally, can such patterns be functionally linked to phenotype, adaptation, or pathogenicity?

Here, we establish a population‐scale pan‐methylome framework for bacteria that integrates long‐read methylation profiling with pangenome‐aware mapping. This framework defines a stable and comparable methylation substrate across strains, introduces quantitative representations of methylation variation, and adapts phyloepigenetic and epigenome‐wide association analyses that have previously been developed primarily in eukaryotes for application to bacterial methylomes. Applied to *Escherichia coli* (*E. coli*), our approach enables systematic interrogation of methylation variation at population scale and provides a generalizable foundation for bacterial epigenomic studies.

## 2 Results

### 2.1 A Diverse Bacterial Population as a Testbed for Pan‐Methylome Method Development

**FIGURE 1:**
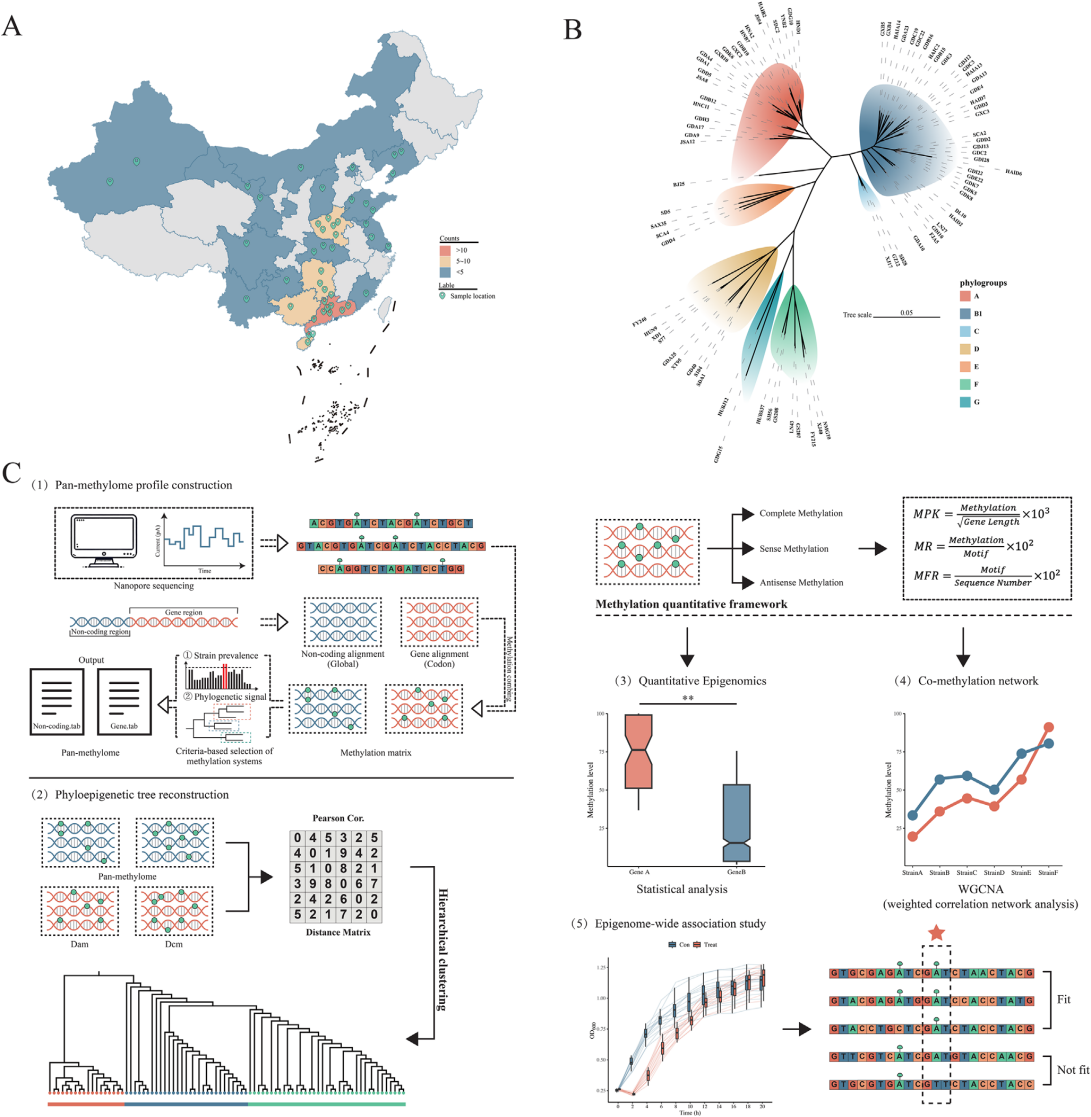
Sample collection, phylogenetic analysis, and pan‐methylome framework of *E. coli*. (A) Map of China showing the geographic distribution of *E. coli* sampling sites. Provinces are colored according to the number of *E. coli* strains collected, and green dots indicate the specific sampling locations. The map was generated using the amCharts Pixel Map Generator (https://pixelmap.amcharts.com). (B) Phylogenetic tree of 84 *E. coli* isolates. The tree was constructed using core genome SNPs and rooted at the midpoint. Different colors represent distinct phylogroups. (C) Analytical framework for pan‐methylome analysis of *E. coli*. The workflow consists of five main steps: pan‐methylome profile construction, phyloepigenetic tree reconstruction, quantitative epigenomics, co‐methylation network, and epigenome‐wide association study (EWAS).

A collection of 84 *E. coli* strains isolated from avian, human, and swine sources between 2018 and 2020 was assembled. The 84 *E. coli* strains were isolated from 45 sampling sites across 19 Chinese provinces: Guangdong, Fujian, Hunan, Hainan, Liaoning, Shandong, Guangxi, Jiangsu, Sichuan, Yunnan, Beijing, Hubei, Henan, Gansu, Guizhou, Inner Mongolia, Shanxi, Shanghai, and Xinjiang (Figure 1A and Table S1). The genome sequences of the 84 *E. coli* strains were determined from 1 Gbp of Oxford Nanopore long reads and 2 Gbp of Illumina short reads. Oxford Nanopore sequencing enabled simultaneous genome sequencing and detection of DNA methylation based on ionic current shifts associated with base modifications. Illumina short reads were used to polish the assemblies and improve base‐level accuracy.

A pan‐genome constructed from the 84 isolates identified a total of 18 464 genes, including 2828 core genes (prevalence ≥ 99%), 630 soft core genes (95% ≤ prevalence < 99%), 2598 shell genes (15% ≤ prevalence < 95%), and 12 408 cloud genes (prevalence < 15%) (Figure S1). Phylogenetic relationships were resolved using a maximum likelihood tree based on core‐gene SNPs, which aligned with traditional phylogroup classification. Seven phylogroups (A, B1, C, D, E, F, G) were identified, with A and B1 comprising 69% of the isolates [11] (Figure 1B).

### 2.2 Filtering Methylation Systems by Population Prevalence and Phylogenetic Signal

**FIGURE 2:**
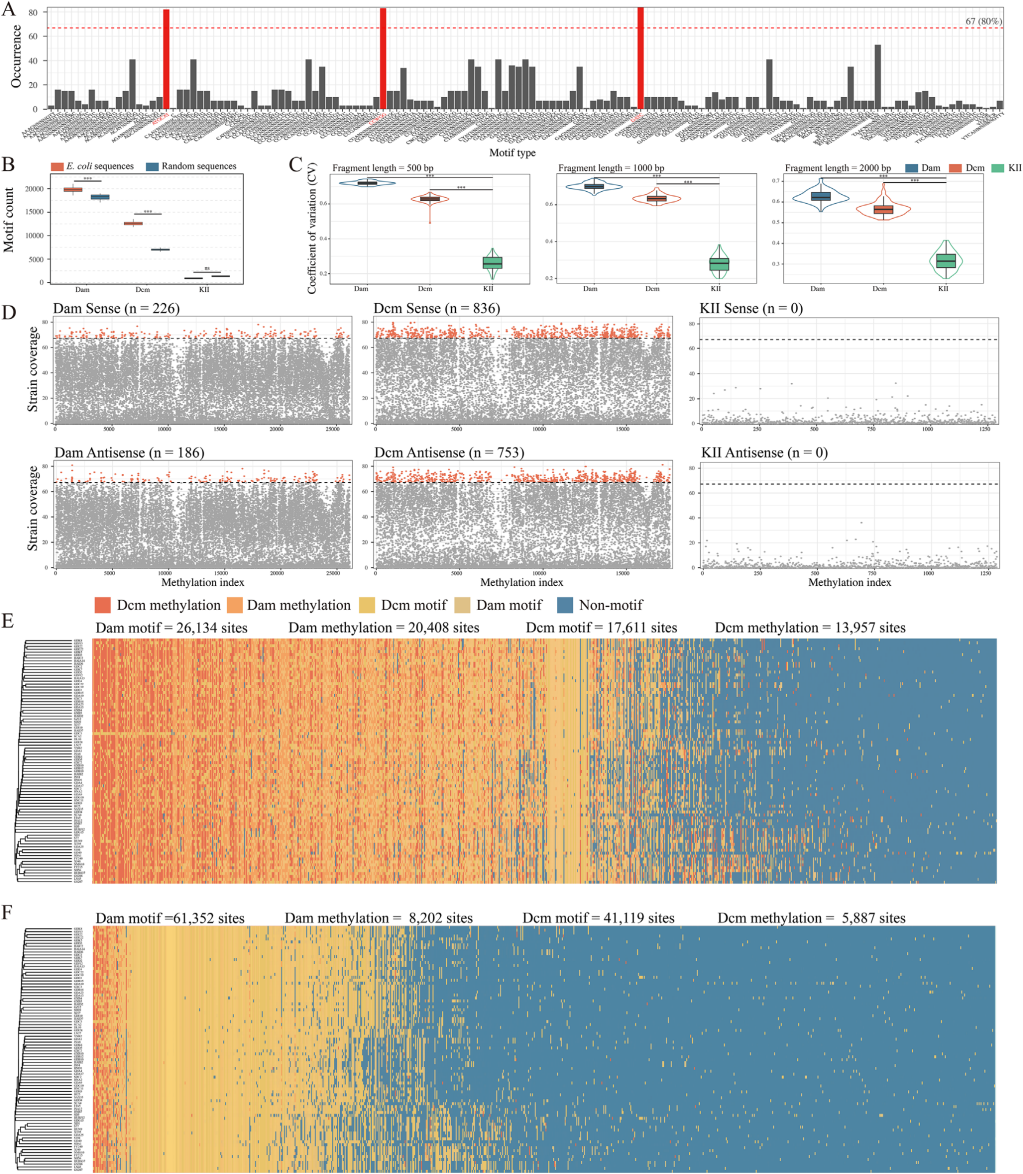
Methylation type selection and pan‐methylome construction. (A) Presence/absence profiles of all methyltransferases (MTases) from REBASE across 84 *E. coli* strains. The x‐axis represents the recognition motifs of the MTases and the y‐axis represents the coverage among 84 *E. coli* strains. (B) Simulation of genomes for the 84 strains based on the phylogenetic tree and the substitution model. Comparison of motif counts of three MTase types (Dam, Dcm, KII) between simulated and real genomes. (C) Coefficient of variation (CV) of the three MTase‐associated motifs across different genome fragment lengths, reflecting motif distribution variability. (D) Methylation sites present in more than 80% of the 84 *E. coli* strains were defined as core methylation sites. The *y*‐axis represents the number of strains (out of the 84 *E. coli* isolates) in which a given methylation site among gene regions is detected (Table S2). (E) The pan‐methylome constructed by sense Dam and sense Dcm methylation in gene regions. (F) The pan‐methylome constructed by sense Dam and sense Dcm methylation in non‐coding regions. As indicated, different colors represent each type of methylation, motif, and non‐motif. Group comparisons were performed using the one‐sided Mann–Whitney U test. *P* < 0.05 is denoted by *, *p* < 0.01 by **, and *p* < 10^−4^ by ***.

To enable population‐scale comparisons of bacterial methylomes, we developed a modular pan‐methylome analysis framework that integrates long‐read methylation profiling with pan‐genomic architecture (Figure 1C). As a first step, we applied this framework to evaluate the conservation of DNA methylation systems across the *E. coli* population by analyzing 3,636 REBASE‐annotated DNA methyltransferases and their 141 corresponding recognition motifs across 84 genomes [12]. All 141 motifs were detected, reflecting the broad distribution and evolutionary retention of MTase‐mediated methylation throughout the *E. coli* lineage (Figure S2). However, only three motifs, i.e., “GATC”, “CCWGG”, and “ATGCAT”, recognized by Dam, Dcm, and EcoKII MTases, respectively, were highly prevalent (>80%). “GATC” was present in all 84 strains (100%) (Figure 2A).

To investigate whether Dam, Dcm, or EcoKII‐mediated methylation have phylogenetic signals, we analyzed both motif distribution and methylation patterns. For motif‐level evaluation, based on the phylogenetic tree, 84 *E. coli* genomes were simulated, and the numbers of the three recognition motifs were quantified. The number of motifs recognized by Dam and Dcm methylation was significantly higher in real genomes compared to simulated ones, suggesting evolutionary selection rather than random distribution (Figure 2B). Additionally, we calculated the coefficient of variation for motif distribution across genomic fragments of varying lengths. There was significantly greater variability in distribution (*p *< 10^−4^) across all fragment sizes in motifs targeted by Dam and Dcm when compared to EcoKII (Figure 2C).

For methylation‐level evaluation, methylation present in > 80% of strains was defined as core and ≤ 80% as accessory methylation. In the gene region, 412 core methylation sites were identified in Dam methylation, while 1589 core sites were identified in Dcm methylation (Table s2). Notably, no core methylation sites were detected for EcoKII methylation, indicating its limited phylogenetic conservation across the population (Figure 2D). Gene Ontology (GO) functional annotation revealed that genes associated with these core methylation sites were predominantly involved in biosynthetic and metabolic processes (Figure S3). These findings suggest that Dam and Dcm methylation carry stronger phylogenetic signals than EcoKII methylation. Consequently, we prioritized Dam (“GATC”) and Dcm (“CCWGG”) methylation, both mediated by orphan MTases, as robust markers for bacterial methylation analysis.

### 2.3 Construction of the *E. coli* Pan‐Methylome Profile Enabled by High‐Throughput Methylation Level Analysis

Dam and Dcm methylation occurred in both gene and non‐coding regions. To minimize bias from sparsely represented genes, the analyses were restricted to 4375 genes present in > 10 of the 84 *E. coli* genomes. Linear regression analysis revealed no dependence of methylation counts on sequencing depth (regression coefficient between −8.46 and −3.24), validating coverage saturation and ensuring robust, unbiased detection for downstream analyses (Figure S4). Methylation was more prevalent in gene regions than in non‐coding regions, with 88.4% and 88.2% of genes showing Dam sense and antisense hemi‐methylation, respectively, and 84.7% and 84.6% showing Dcm sense and antisense hemi‐methylation (Figure S5A). In contrast, only ∼36% of non‐coding regions had associated Dam or Dcm methylation in either orientation. In addition, we assessed methylation in gene and non‐coding regions by calculating the Observed / Expected (O/E) ratios with zero‐order and first‐order Markov models. For both MTases, methylation O/E ratios were significantly elevated in gene regions compared with non‐coding regions (*p* < 10^−4^) (Figure S5B). These findings highlight the necessity of considering methylation patterns in both Dam/Dcm and gene/non‐coding regions. We further compared the O/E ratios between the 200 bp regions upstream of coding sequences and the entire coding regions, but found no significant differences. In contrast, we identified 47 core methylation sites within the upstream 200 bp regions. Fisher's exact test revealed that these sites were significantly enriched relative to non‐coding regions, particularly for 5mC on both the sense and antisense strands and for 6mA on the antisense strand (Table S3). Thus, despite the absence of significant differences in overall O/E ratios, conserved core methylation sites showed preferential enrichment in the upstream regions of coding sequences.

Based on Dam/Dcm methylation analysis, we constructed a pan‐methylome profile for both sense and antisense strands in gene and non‐coding regions. A total of 1 46 216 motif positions were analyzed, with 51 668 positions showing methylation. In gene regions, 43 745 motif sites were identified, with 26 134 recognized by Dam and 17 611 by Dcm MTases, respectively. On the sense strand, 20 408 motif sites underwent Dam and 13,957 sites underwent Dcm methylation (Figure 2E). On the antisense strand, 20,331 motif sites underwent Dam methylation, and 13 872 sites underwent Dcm methylation (Figure S6A). In non‐coding regions, 102,471 motif sites were identified, with 61 352 motifs recognized by Dam MTase and 41 119 motifs by Dcm MTase (Figure 2F). On the sense strand, 8202 motif sites underwent Dam methylation, and 5887 sites underwent Dcm methylation. On the antisense strand, 8267 motif sites underwent Dam methylation, and 5816 sites underwent Dcm methylation (Figure S6B). These results provide a comprehensive overview of the distribution of Dam and Dcm methylation across both gene and non‐coding regions, and provided essential data for subsequent analyses.

### 2.4 Pan‐Methylome–Based Reconstruction of Bacterial Phyloepigenetic Tree

**FIGURE 3:**
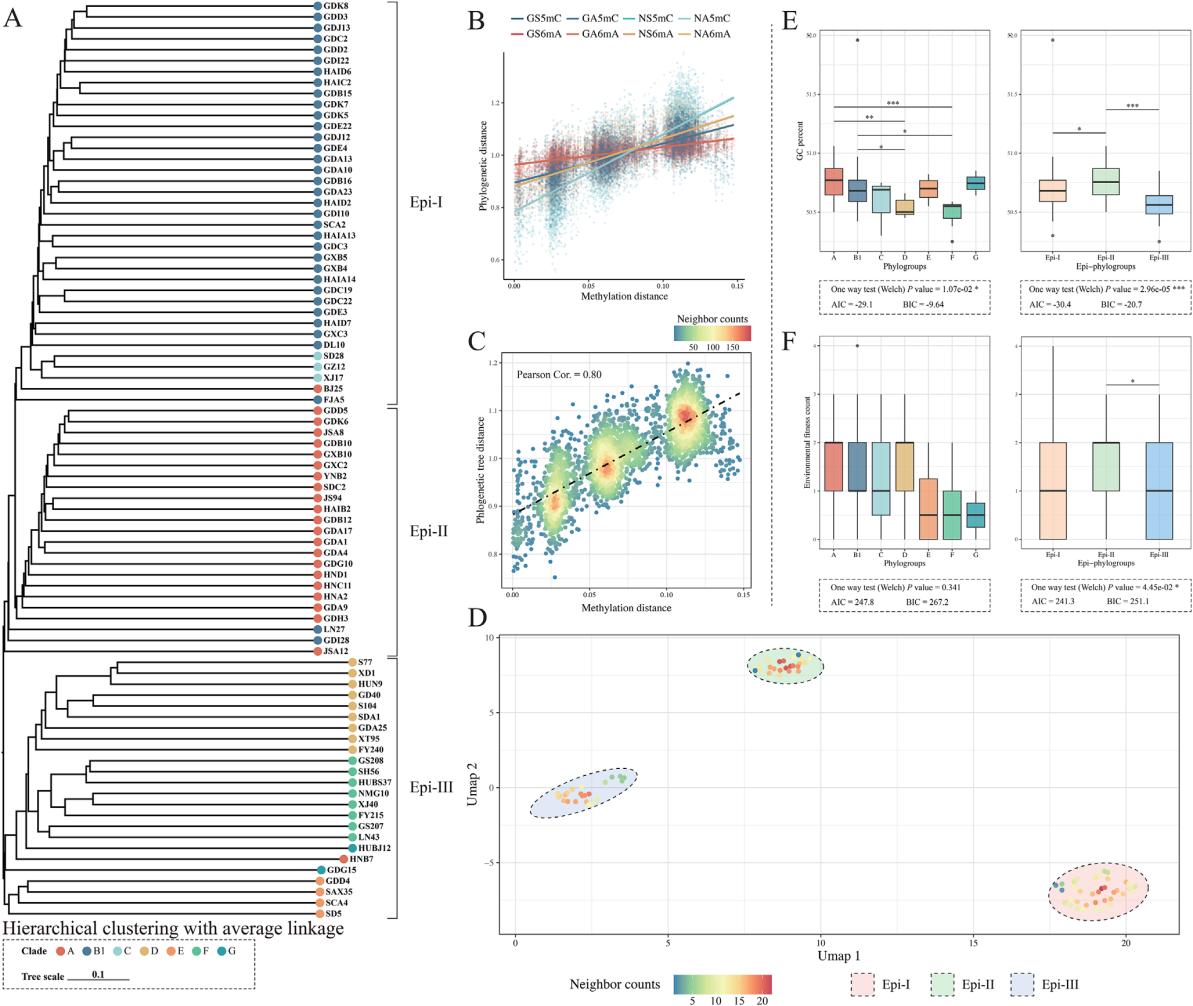
Phyloepigenetic trees provide enhanced resolution for the classification of *E. coli*. (A) Phyloepigenetic tree of 84 *E. coli* isolates constructed based on Dam and Dcm methylation across both gene and non‐coding regions of the core genome using hierarchical clustering with average linkage. Node colors correspond to the phylogroups assigned in the traditional phylogenetic tree. (B) Correlations between phylogenetic distances and eight types of methylation distance, including Dam gene sense methylation, Dam gene antisense methylation, Dcm gene sense methylation, Dcm gene antisense methylation, Dam non‐coding sense methylation, Dam non‐coding antisense methylation, Dcm non‐coding sense methylation, and Dcm non‐coding antisense methylation. (C) Correlation between the integrated methylation distance (combining all eight types) and the phylogenetic distance. Color intensity corresponds to the local density of adjacent points, indicating the degree of regional enrichment. (D) UMAP dimensionality reduction based on the integrated methylation distances reveals three distinct epi‐phylogroups. Each circle indicates the 95% confidence interval for each epi‐phylogroup, and the color of each point indicate the degree of regional enrichment. (E) Comparison of classification performance between phylogroup and epi‐phylogroup based on GC content. (F) Comparative classification performance of phylogroup and epi‐phylogroup in explaining strain environmental fitness. (E, F) Overall differences were assessed using a one‐way Welch's ANOVA, with AIC and BIC used to evaluate model fit. Pairwise group comparisons were conducted using multiple hypothesis testing.

To investigate the epigenetic relationships among the 84 *E. coli* strains, we calculated methylation distances based on the methylation profiles of core genes. To account for heterogeneity in methylation patterns, eight categories were classified: Dam and Dcm methylation in gene and non‐coding regions, both in the sense and antisense strand. Specifically, we analyzed the following categories: Dam gene sense methylation (13 703 sites), Dam gene antisense methylation (13 681 sites), Dcm gene sense methylation (8973 sites), Dcm gene antisense methylation (8946 sites), Dam non‐coding sense methylation (4573 sites), Dam non‐coding antisense methylation (4,640 sites), Dcm non‐coding sense methylation (3272 sites), and Dcm non‐coding antisense methylation (3218 sites). The methylation distances for each category were standardized based on their mean values and then merged and used for hierarchical clustering to construct a phyloepigenetic tree. Comparison of the phyloepigenetic tree with phylogroup information from the phylogenetic tree demonstrated that the epigenetic tree could distinguish strains at the phylogroup level (Figure 3A).

Pearson correlation analysis revealed a positive correlation between methylation distances across all eight categories and phylogenetic distances (Figure 3B). When the merged distances were compared with phylogenetic distances, a significant positive correlation persisted (Pearson Corr. = 0.80), with three distinct “hot spots” identified, suggesting a sub‐cluster structure within the phyloepigenetic tree (Figure 3C). To further investigate this, we downscaled the merged methylation distance matrix using UMAP, revealing that the phyloepigenetic tree could be divided into three sub‐clusters, designated as epi‐phylogroups: Epi‐I, Epi‐II, and Epi‐III (Figure 3D). Mapping the epi‐phylogroups to the phylogroups showed that Epi‐I predominantly consists of the B1 and C phylogroups, Epi‐II of the A phylogroup, and Epi‐III includes the D, E, F, and G phylogroups (Figure S7). Strains from the A and B1 phylogroups were distributed across multiple epi‐phylogroups, indicating epigenetic polymorphism within these phylogroups.

In addition, the study further assessed the effectiveness of phylogroup and epi‐phylogroup classifications based on GC content and the environmental stress response as determined experimentally. For GC content, both classifications were significant, with epi‐phylogroup classification yielding lower *p* values (*p* = 2.96 × 10^−5^), as well as lower AIC and BIC values compared to phylogroup classification (Figure 3E). Regarding the environmental stress response, the 84 *E. coli* strains were exposed to four conditions: acidic (pH = 5), alkaline (pH = 9), high temperature (45°C), and low temperature (25°C). Growth curves were compared to control conditions using paired *t*‐tests, and significance thresholds were determined based on the shift in the number of unaffected strains (Figure S8). This analysis identified 21 high‐temperature‐tolerant, 35 low‐temperature‐tolerant, 37 acid‐tolerant, and 15 alkali‐tolerant strains. No significant differences were associated with phylogroup classification. However, there were significant differences (*p *< 0.05) associated with epi‐phylogroup classification with lower AIC (241.3) and BIC (251.1) values (Figure 3F). These findings suggest that epi‐phylogroups better capture phenotypic differences between strains compared to phylogroups, indicating superior classification capabilities, a novel perspective on the phylogenetic classification of *E. coli* based on bacterial DNA methylation.

### 2.5 Quantitative Methylation Features Enable Inference of Gene Conservation and Essentiality

Comparison across these two methylation types (Dam, Dcm) indicated that complete methylation was significantly (*p* < 10^−4^) more prevalent than both sense and antisense hemi‐methylation in the 84 *E. coli* strains (Figures S9 and S10). To explore the potential relationship between methylation levels and gene prevalence, we investigated the correlation between the amount of complete methylation and gene prevalence. As a positive control, a large pan‐genome consisting of 2373 *E. coli* genomes was constructed. We then categorized the 4375 genes (mentioned above) into eight groups, including essential genes, 100% prevalence genes, core genes, and five additional groups based on gene prevalence thresholds of 10% (90%∼99%, 80%∼90%, 70%∼80%, 60%∼70%, and <60%). This approach allowed us to systematically assess how methylation patterns correlate with gene prevalence across different categories.

**FIGURE 4:**
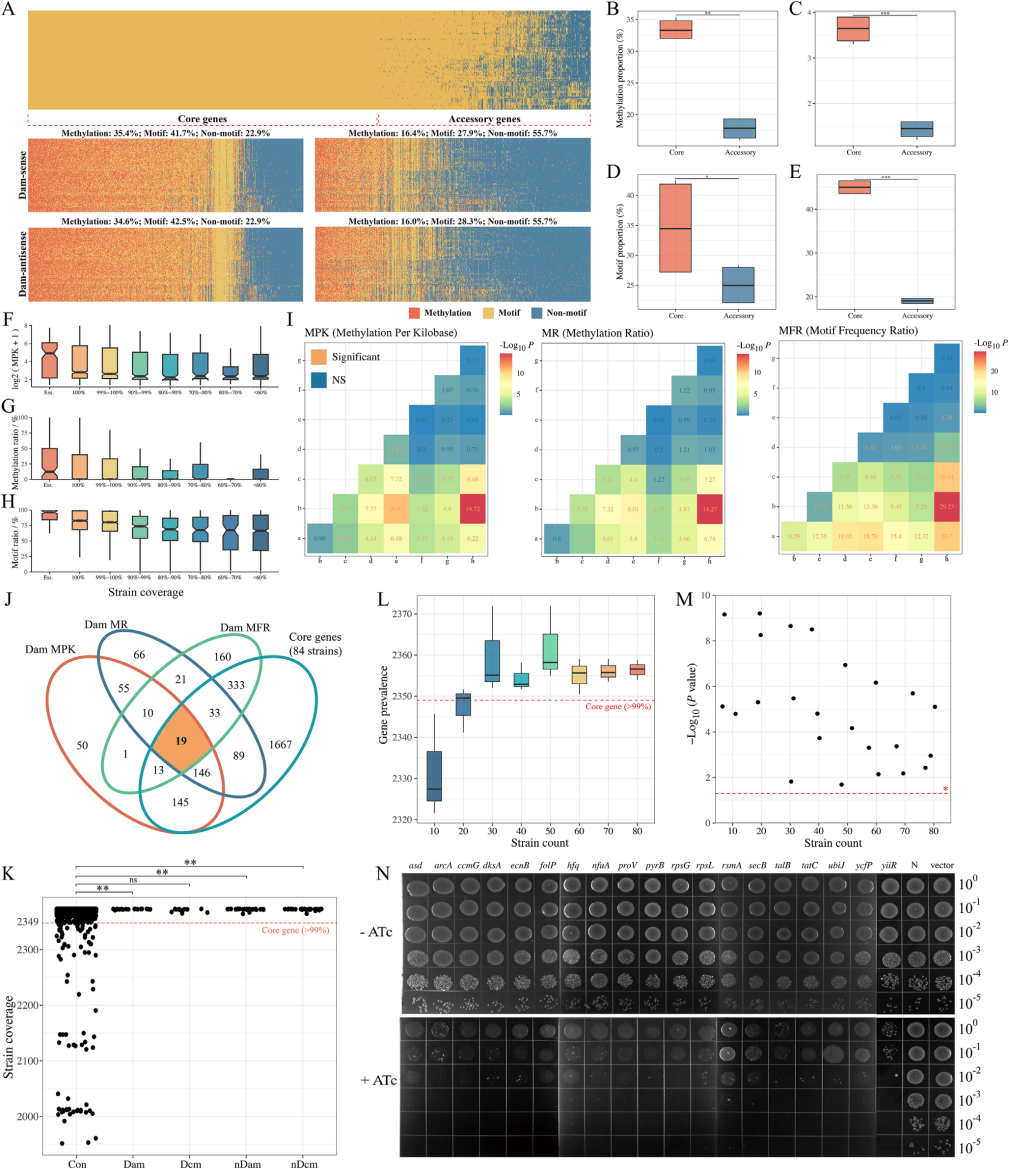
Dam methylation quantification of gene regions is associated with gene prevalence and gene quasi‐essentiality. (A) Dam methylation profiles of gene regions in core and accessory genes of 84 *E. coli* strains, showing the proportions of methylation, unmethylated, and missing motifs. (B‐E) Comparison of motif and methylation proportions between core and accessory genes in gene and non‐coding regions across 84 *E. coli* strains. Methylation proportions in gene (B) and non‐coding (C) regions. Motif proportions in gene (D) and non‐coding (E) regions. Statistical significance was assessed using one‐tailed paired t‐tests. (F–H) For Dam methylation in gene regions, the distributions of methylation per kilobase (MPK, F), methylation ratio (MR, G), and motif frequency ratio (MFR, H) are shown across essential genes and genes with varying prevalence. (I) The color of each square indicates the –log10 *p* value, and the font color indicates whether the difference between groups is statistically significant (*p* < 0.05). Gene groups are as follows: (a) essential genes, (b) genes with prevalence = 100%, (c) prevalence 99%–100%, (d) 90%–99%, (e) 80%–90%, (f) 70%–80%, (g) 60%–70%, and (h) < 60%. Group comparisons were performed using the one‐sided Mann–Whitney U test. (J) Identification of high‐methylation core genes based on Dam methylation in gene regions. Genes in the top 10% for MPK, MR, and MFR were intersected with the core genes shared by 84 *E. coli* strains, resulting in the identification of 19 high‐methylation core genes. (K) Comparison of gene prevalence between the core genes of 84 *E. coli* strains and high‐methylation core genes across 2373 strains. Each point represents the prevalence of a gene among the 2373 strains, with the red dashed line indicating the prevalence threshold for defining core genes (99%) in the 2373 strains. The prevalence of core genes from the 84 strains was compared with that of high‐methylation core genes identified in gene regions (Dam and Dcm methylation) and non‐coding regions (Dam and Dcm methylation) using the one‐sided Mann–Whitney U test. (L‐M) Impact of reducing the number of analyzed strains on high‐methylation core gene detection. (L) The *x*‐axis represents the number of strains used for analysis, and the y‐axis indicates the gene prevalence of high‐methylation core genes across 2373 *E. coli* strains. For each strain count, three random subsets were selected for analysis. (M) One‐sided Mann–Whitney U tests were performed to compare the gene prevalence (2373 *E. coli* strains) of high‐methylation core genes and core genes at each strain count. The red dashed line represents the significance threshold of *p* = 0.05. (N) CRISPRi knockdown of 19 high‐methylation core genes identified based on Dam methylation in gene regions to assess their quasi‐essentiality. aTc = anhydrotetracycline. Impaired or reduced growth in the presence of aTc at an equivalent dilution indicates gene quasi‐essentiality. *P* < 0.05 is denoted by *, *p* < 0.01 by **, and *p* < 10^−4^ by ***.

Comparison of pan‐methylation profiles between core and accessory genes found that, within both gene and non‐coding regions, the proportion of Dam and Dcm methylation, as well as motif on the sense and antisense strands were significantly (*p* < 10^−4^) higher in core genes than in accessory genes (Figure 4A–E and Figures S11A,S12A,S13A). Furthermore, we quantified methylation levels across gene groups using three indicators: methylation per kilobase (MPK), methylation ratio (MR), and motif frequency ratio (MFR). Strikingly, essential genes had significantly (*p* < 10^−4^) higher Dam complete methylation across all three indicators (MPK, MR, and MFR) compared to non‐essential genes, with core genes also displaying elevated levels relative to low‐prevalence genes (Figure 4F–I). A similar pattern was seen for Dcm complete methylation in gene regions, with essential and high‐prevalence genes consistently showing higher methylation levels (*p* < 10^−4^) (Figures S11B and 14A). The same trend was observed for Dam complete methylation in non‐coding regions, where regions adjacent to core and essential genes had significantly (*p* < 0.05) higher methylation levels than those near low‐prevalence genes (Figures S12B and S14B). Dcm complete methylation in non‐coding regions also displayed significantly (*p* < 10^−3^) higher MPK, MR, and MFR values in areas close to essential or core genes (Figures S13B and S14C).

In addition, the top 10% of genes for each indicator (MPK, MR, and MFR values) were compared with the core genes of 84 *E. coli* strains, identifying high‐methylation core genes (Figure 4J and Figures S11C, S12C, and S13C). Strain coverage statistics for the four high‐methylation core gene datasets in 2,373 *E. coli* strains showed that the three groups (gene Dam, non‐coding Dam, and non‐coding Dcm) had significantly higher coverage than the core genes of our 84‐strain collection (*p* < 0.01) (Figure 4K). Further investigation demonstrated that methylation quantification based on a limited number of strains was sufficient to recover the core genome of 2373 *E. coli* strains. As few as 30 genomes were adequate to identify high‐methylation core genes with a prevalence comparable to that of the core genes in the 2373 *E. coli* strains (Figure 4L). Additionally, methylation analysis, even with a reduced number of strains, significantly improved gene prevalence (*p* < 0.05) (Figure 4M). This analysis revealed that, despite using fewer strains, methylation patterns, when combined with genomic data, reliably identified high‐methylation core genes that met the standard of the full 2373‐strain core genome.

Moreover, we selected the Dam group, which contained 19 high‐methylation core genes with the highest strain coverage, for CRISPRi knockdown to assess their essentiality (Figure 4N). Notably, all 19 high‐methylation core genes were quasi‐essential genes, i.e., their knockdown resulted in impaired growth [13]. Our results underscore the association between bacterial core genes, essential genes, and methylation, and highlight the high value of methylation quantitative comparison.

### 2.6 Co‐methylation Network Analysis Reveals Functional Organization of Bacterial Methylomes

**FIGURE 5:**
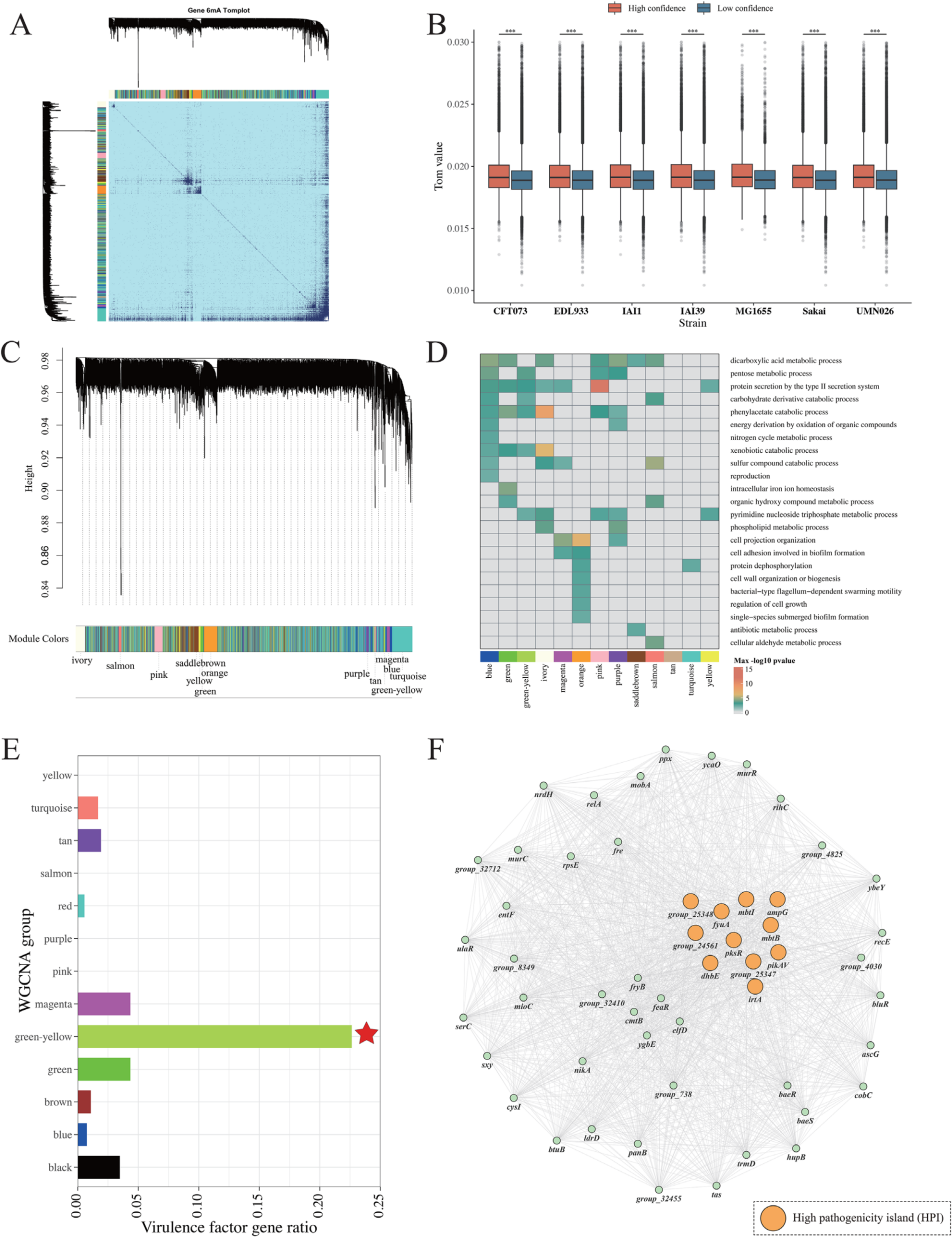
Co‐methylation network based on Dam methylation in gene regions is associated with gene connectivity, biological processes, and virulence. (A) TOM (Topological Overlap Matrix) of Dam methylation in gene regions. (B) Comparison of TOM values between high‐confidence and low‐confidence gene pairs across seven *E. coli* strains in the STRING database, including CFT073, EDL933, IAI1, IAI39, MG1655, and UMN026. Statistical significance was determined using a two‐sided Mann–Whitney U test. (C) Consensus co‐methylation network based on Dam methylation in gene regions, constructed using complete, sense, and antisense methylation data. Modules containing at least 20 genes were identified. The gene names for each module are provided in Table S1. (D) GO biological process annotations for each consensus co‐methylation module. Functional enrichment was performed using the ClusterProfiler2 package and grouped under broader ontology terms using the “rrvgo” package. (E) Proportion of virulence factors within each module. The green‐yellow module exhibited the highest percentage of virulence genes and was selected for downstream analyses. (F) Co‐methylation network of the green‐yellow module, clustered based on TOM values using the force‐directed algorithm. Yellow nodes indicate genes located in the high pathogenicity island (HPI). *P* < 0.05 is denoted by *, *p* < 0.01 by **, and *p* < 10^−4^ by ***.

We constructed four consensus co‐methylation networks stratified by methylation type (Dam/Dcm) and genomic context (gene/non‐coding), using three quantitative measures (complete methylation, sense, and antisense hemi‐methylation). WGCNA soft thresholds were determined by mean connectivity (Figures S15 and S16). Topological overlap matrices (TOM) revealed distinct modular architectures (Figure 5A and Figures S17A, S18A, and S19A). Functional relevance analysis showed TOM values significantly correlated with STRING gene interaction confidence (*p* < 0.001), with higher TOM values for gene‐region Dam and Dcm methylation under high‐confidence interactions (Figure 5B and Figure S17B). In contrast, non‐coding region methylation showed more variable TOM patterns in gene interaction across strains (Figures S18B and S19B). These findings suggest gene‐level co‐methylation may reflect the connectivity of gene interaction networks.

The four consensus co‐methylation networks were further partitioned into 11 to 14 modules, each displaying distinct functional enrichment profiles (Figure 5C, Figures S17C, S18C, S19C, and Tables S4, S5, S6, and S7). Dam‐associated gene methylation modules were enriched in metabolic processes, homeostasis, cell organization, adhesion, biofilm formation, motility, and reproduction (Figure 5D). In contrast, Dcm‐related modules were linked to protein modification, transport, stress responses, and motility (Figure S17D). Modules derived from non‐coding region methylation suggested regulatory roles in biosynthesis, stress adaptation, and cellular organization (Figures S18D and S19D). These results highlight DNA methylation as a multilayered regulator of bacterial physiology and environmental adaptation.

To investigate the relationship between methylation and pathogenicity, we analyzed the Dam gene co‐methylation network for enrichment of virulence‐associated genes. Among the 4375 genes examined, 440 were identified as virulence factors based on the VFDB database, with 47 present in the 84 *E. coli* strains (Figure S20). The green‐yellow module contained a higher proportion of virulence genes among the 13 co‐methylation modules (Figure 5E). Clustering based on TOM values found that the *E. coli* High‐Pathogenicity Island (HPI), which promotes iron acquisition and enhances pathogenicity, was found within this module (Figure 5F). Furthermore, 42 genes related to the HPI were identified, highlighting their potential role in pathogenicity. These results suggest that the green‐yellow module may act as a key epigenetic regulatory hub for *E. coli* virulence, and its constituent genes could represent potential targets for therapeutic intervention.

### 2.7 Adapting EWAS to Bacterial Populations

**FIGURE 6:**
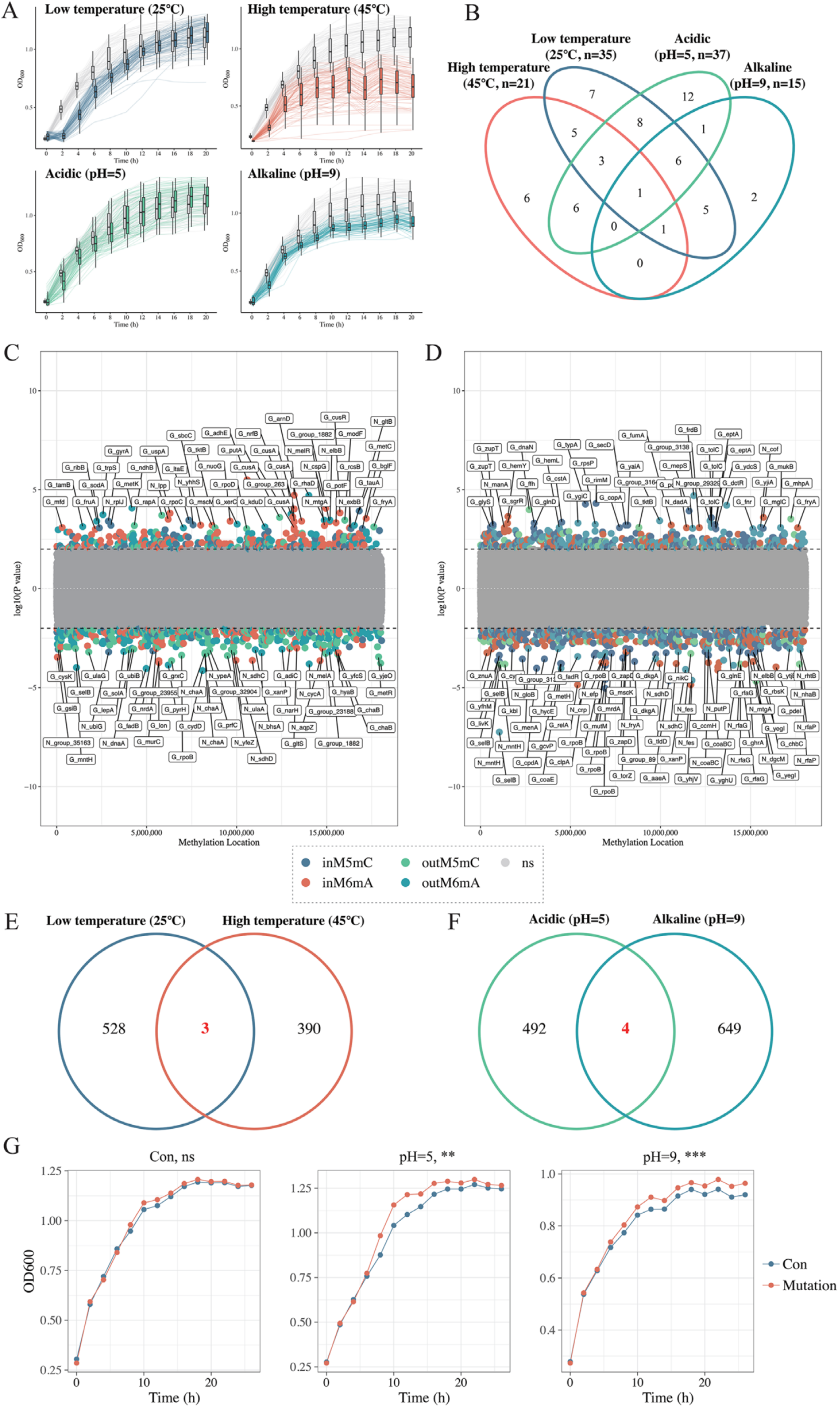
Epigenome‐wide association analysis (EWAS) of the *E. coli* environmental stress response. (A) Growth curves of 84 *E. coli* strains under normal conditions (gray) and under stress conditions including low (25°C) and high temperature (45°C), and acidic (pH = 5), and alkaline (pH = 9) conditions. (B) Stress‐tolerant strains under each condition were identified using a two‐sided paired t‐test. A Venn diagram illustrates the overlap and distribution of tolerant strains across the different environmental conditions. (C–D) DNA methylation sites associated with environmental stress responses in 84 *E. coli* strains. (C) Methylation sites linked to the temperature response. The upper panel shows sites associated with high‐temperature conditions, and the lower panel shows sites related to low‐temperature response. (D) Methylation sites associated with the pH response. The upper panel displays sites linked to acidic stress, while the lower panel shows those related to alkaline conditions. In both panels, methylation sites are annotated with the corresponding genes: labels beginning with “*G*” represent gene regions, and those beginning with “N” indicate non‐coding regions. (E) Venn diagram of significantly associated methylation sites under low‐ and high‐temperature environments, identifying temperature‐responsive methylation sites. (F) Venn diagram of significantly associated methylation sites under acidic and alkaline environments, identifying pH‐responsive methylation sites. (G) Growth curve comparison before and after synonymous mutation that eliminates a pH‐associated methylation site. A degenerate codon substitution was introduced to disrupt the methylation motif without altering the encoded amino acid. Growth was measured under control (pH = 7), acidic (pH = 5), and alkaline (pH = 9) conditions. Statistical significance was assessed using paired *t*‐tests. *P* < 0.05 is denoted by *, *p* < 0.01 by **, and *p* < 10^−4^ by ***.

Growth profiling of 84 *E. coli* strains under thermal and pH stress identified one broadly tolerant strain, with 10 temperature‐insensitive and 8 pH‐insensitive strains (Figure 6A,B). EWAS comparing tolerant and intolerant strains under four stress conditions identified 1202 Dam and 864 Dcm significant methylation sites linked to environmental adaptation. Specifically, 393 (170 Dam methylation, 95 Dam demethylation, 82 Dcm methylation, 46 Dcm demethylation) sites were associated with high temperature; 531 (127, 178, 40, 186) with low temperature; 496 (101, 206, 42, 147) with acidic stress; and 653 (174, 151, 68, 260) with alkaline stress (Figure 6C,D).

We identified three methylation sites linked to both high and low temperature adaptation and four sites associated with acidic and alkaline stress, all located at Dcm demethylation positions (Figure 6E,F). Targeted mutagenesis of a pH‐associated Dcm site, abolishing methylation without altering the protein sequence, significantly enhanced strain adaptability to pH stress (*p* < 10^−4^) (Figure 6G). These results highlight methylation as a key regulator of environmental stress responses and a potential target for engineering microbial resilience.

## 3 Discussion

In this study, we establish a population‐scale pan‐methylome framework that enables systematic, comparative analysis of bacterial DNA methylation beyond single genomes and site‐level descriptions. By integrating long‐read methylation profiling with pan‐genomic architecture, we provide a unified representation of methylation variation across strains, genomic contexts, and evolutionary lineages. This framework enables bacterial methylomes to be interrogated as structured, population‐level entities by actively adapting and re‐engineering epigenomic analytical concepts established in eukaryotes, rather than directly transferring them, to accommodate the distinct data structures and regulatory logic of bacterial methylation systems. Whereas epigenomic phylogenetic analyses in eukaryotes are typically applied at the species or higher taxonomic levels, our approach extends phyloepigenetic resolution in bacteria to the level of closely related phylogroup within a species. Together, our approach lays a methodological foundation for quantitative, comparative, and phylogenetically informed analysis of bacterial epigenomes.

To construct a population‐scale methylation atlas suitable for comparative analysis, we implemented a series of methodological constraints to ensure robustness and cross‐strain comparability. First, bacterial cultures were collected at the logarithmic growth phase, a condition under which methylation heterogeneity between strains can be reliably captured and is minimally confounded by growth‐state–dependent variability [14]. For methylation calling, only sites consistently detected as methylated across all sequencing reads within a strain were retained, thereby minimizing stochastic noise and emphasizing reproducible, strain‐level methylation patterns.

A central requirement for population‐scale analysis is the definition of a stable and comparable methylation substrate across strains. We therefore restricted downstream analyses to DNA methylation motifs that are both broadly conserved and phylogenetically informative. All motifs considered in this study were required to have corresponding genome‐encoded DNA methyltransferases (MTases), ensuring enzymatic determinism and excluding non‐enzymatic or context‐dependent modifications [12]. Conserved, MTase‐linked motifs provide stable epigenetic landmarks that support consistent cross‐genome alignment and quantitative comparison across divergent lineages [15, 16]. Moreover, motifs exhibiting lineage‐associated enrichment are expected to encode phylogenetic signal, reflecting selective pressures acting on epigenetic regulation and capturing functionally relevant divergence among strains [17].

Applying these criteria, we systematically screened motif occurrence, MTase distribution, and site‐specific methylation patterns across 84 *E. coli* strains (Figure 2A–D). Although many REBASE‐annotated motifs were detectable at the genomic level, only three methylation systems—Dam, Dcm, and EcoKII—were widely encoded across the population (Figure 2A). Among these, Dam‐ and Dcm‐mediated methylation exhibited robust phylogenetic structure, whereas EcoKII showed limited conservation at the methylation‐site level (Figure 2D and Figure S3). Importantly, Dam and Dcm are catalyzed by orphan MTases, rather than restriction–modification–associated enzymes, conferring heritable and regulatory methylation patterns that are well suited for comparative and evolutionary analysis [18, 19, 20, 21, 22, 23, 24, 25, 26]. On the basis of these analytically defined criteria, we selected Dam‐ and Dcm‐targeted methylation sites as the core substrate for pan‐methylome profile construction, prioritizing cross‐strain comparability and phylogenetic interpretability.

Although this filtering strategy enabled robust and comparable population‐scale analysis, it necessarily restricted the methylome fraction examined in the present study. Therefore, our conclusions should be interpreted as applying primarily to a conserved Dam/Dcm‐centered methylome layer, rather than to the complete *E. coli* methylome. Other methylation systems, including accessory MTases, phase‐variable MTases, restriction–modification‐associated MTases, and MTases recognizing bipartite or complex motifs, were excluded or underrepresented in our study [27]. This filtering likely reduces the detectable contribution of strain‐specific, mobile element‐associated, and condition‐dependent methylation programs to *E. coli* methylome diversity.

This limitation has several implications for the interpretation of our results. First, the present analysis may not fully capture rapidly switching epigenetic states generated by phase‐variable MTases [27]. Second, because the EWAS and co‐methylation analyses were based on Dam‐ and Dcm‐mediated methylation profiles, the resulting associations should be interpreted as reflecting this conserved methylation layer rather than the regulatory contributions of accessory, phase‐variable, or bipartite‐motif MTase systems. Third, because Type I restriction–modification systems often recognize bipartite motifs and can generate lineage‐ or strain‐specific methylation patterns, their exclusion may lead to an underestimation of the contribution of R‐M systems to epigenetic diversification, horizontal gene transfer barriers, and niche adaptation [28]. Thus, the present framework should be viewed as a conservative and comparable representation of the stable *E. coli* methylome, rather than as an exhaustive catalog of all methylation‐mediated regulatory variation. Future extensions incorporating de novo motif discovery, phase‐state resolution, transcriptional profiling, and improved modeling of bipartite or complex motifs will be required to integrate accessory and dynamic MTases into a more comprehensive bacterial pan‐epigenomic framework.

In eukaryotic systems, epigenomic phylogenetic reconstruction has emerged as a powerful analytical strategy for resolving sample relationships beyond DNA sequence variation [29]. In this study, we extend this analytical paradigm to bacteria and establish a methylation‐based framework for phyloepigenetic reconstruction using pan‐methylome profiles. Our analysis classified 84 *E. coli* strains into three epi‐phylogroups (Epi‐I, Epi‐II, and Epi‐III), revealing an epigenetic structure that partially recapitulates but also refines traditional genome‐based phylogeny (Figure 3). Notably, comparative analyses of GC content and environmental stress phenotypes support the notion that epi‐phylogrouping captures inter‐strain variation in adaptive capacity more effectively than genome‐based phylogeny (Figure 3E,F). Phylogroup E, traditionally clustered with A, B1, and C, instead aggregated with D, F, and G in Epi‐III, suggesting shared epigenetic and adaptive features across these lineages [11]. Furthermore, strains from phylogroups A and B1 were dispersed across multiple epigenetic subclusters, indicating substantial epigenetic heterogeneity that may reflect functional plasticity or niche‐specific adaptation (Figure S7). These findings highlight the potential of methylome‐informed phylogenetics to provide an additional layer of resolution in microbial classification and evolutionary studies, particularly at species and sub‐lineage levels.

To enable quantitative, gene‐level comparison of bacterial methylomes, we defined three complementary indicators—methylation per kilobase (MPK), methylation ratio (MR), and motif frequency ratio (MFR)—that convert site‐level methylation signals into standardized, population‐scale features. Together, these metrics normalize for gene length, motif availability, and pan‐genome presence–absence, providing a unified quantitative representation of strand‐specific methylation across genes and non‐coding regions. Using this framework, we observed that core genes exhibit consistently higher methylation levels than accessory genes and are strongly enriched for essentiality (Figure 4). Remarkably, highly methylated genes identified from as few as 30 strains partially recapitulated the core genome defined from over 2300 *E. coli* isolates, and incorporation of methylation‐derived features improved core gene recovery across sample sizes. These results demonstrate that quantitative pan‐methylome features capture functional constraints beyond sequence conservation alone and provide a complementary strategy for identifying core and essential genes in bacterial populations.

We found that all 19 high‐methylation core genes, identified through their epigenetic signatures, were experimentally validated as quasi‐essential (Figure 4N). While previous studies relied on transposon mutagenesis or CRISPRi screens to identify such genes, our methylation‐based approach offers a complementary strategy [30, 31, 32]. Notably, three of the nineteen quasi‐essential genes uncovered in this study, including *hfq*, *proV*, and *arcA*, are well‐documented regulators of bacterial stress responses [33, 34, 35]. The enrichment of such regulators among highly methylated quasi‐essential genes suggests that DNA methylation serves as a regulatory nexus linking core physiological processes to adaptive responses essential for environmental resilience and pathogenicity, and is further supported by our epigenome‐based phylogroup classification, which identified strong links between methylation patterns and ecological adaptation. In addition, we applied EWAS, originally developed for eukaryotic 5mC profiling, to identify Dam‐ and Dcm‐associated methylation sites that predict the tolerance of *E. coli* to temperature and pH extremes (Figure 6) [36, 37, 38, 39, 40]. Targeted disruption of these sites confirmed their contribution to stress adaptation, illustrating how population‐scale methylation analysis can be used to link epigenetic variation to phenotypic traits and to nominate candidate regulatory loci for functional interrogation.

In this study, we found that although the non‐coding regions of the genome contain a greater number of potential methylation motifs than gene regions, most of these sites remain unmethylated (Figure S5). Moreover, core methylation sites were preferentially enriched upstream of coding sequences compared with the total non‐coding region. This pattern is consistent with selective accessibility of methylation sites and suggests that non‐coding regions provide a heterogeneous background against which functionally relevant methylation can be resolved [25, 41, 42, 43]. Such selective protection may serve a regulatory function, allowing dynamic control of gene expression and enabling *E. coli* to better adapt to environmental fluctuations [44]. To further explore the functional significance of bacterial methylation, we assessed the biological relevance of co‐methylation patterns by comparing TOM values with STRING gene interaction scores, finding significantly higher TOM values for high‐confidence gene pairs across all seven *E. coli* datasets [45, 46] (Figure 5B and Figures S17B, S18B, and S19B). Interestingly, clustering based on TOM values found that HPI were grouped into a distinct subcluster, suggesting that this module may play a key role in the methylation‐mediated regulation of pathogenicity (Figure 5F). Accordingly, genes within the green‐yellow module may serve as promising targets for future studies aiming to develop epigenetic‐based strategies to mitigate *E. coli* virulence. Horizontal gene transfer (HGT) and epigenetic regulation coalesce to enable the assimilation of foreign DNA into host regulatory networks, but empirical evidence for this process has been lacking. Recent studies have shown that DNA acquired through horizontal gene transfer is preferentially targeted for epigenetic silencing and exhibits a negative correlation with phylogenetic consistency within the same genomic region in fungi [47]. Here, we show that the *E. coli* HPI clusters within a conserved co‐methylation module, providing the first population‐scale demonstration that horizontally acquired loci are not merely genomically embedded but have undergone epigenetic integration into the host genome's regulatory circuitry.

Collectively, our work transforms bacterial DNA methylation from a descriptive catalogue of modified sites into a quantitative, evolvable, and population‐comparable epigenomic system. By defining principled criteria for methylation system selection, introducing pan‐methylome–aware quantitative features, and establishing the first phyloepigenetic, association, and network‐based analyses in bacteria, this study closes a long‐standing methodological gap between prokaryotic and eukaryotic epigenomics. Importantly, these advances do not rely on scaling up existing eukaryotic pipelines, but instead emerge from re‐engineering epigenomic concepts around the unique enzymatic and genomic logic of bacterial methylation. As long‐read sequencing continues to expand across microbial systems, this work provides a blueprint for interrogating epigenetic variation as an integral component of bacterial evolution, adaptation, and pathogenicity.

## 4 Materials and Methods

### 4.1 Strain Collection, Growth and Whole‐Genome Sequencing

A total of 84 *E. coli* strains (63 avian, 20 porcine, and one human) collected between 2018 and 2020 were used in this study. The avian strains were collected from 24 poultry farms across 10 provinces in China, the 20 porcine strains were collected from swine farms across 12 provinces, and the single human‐derived strain was isolated from a clinical sample [48]. The human‐derived clinical isolate used in this study was obtained from a patient enrolled in an approved clinical research project. The study protocol was reviewed and approved by the Medical Ethics Committee of Tongji Hospital, Tongji Medical College, Huazhong University of Science and Technology (Approval No. TJ‐IRB20230836). Written informed consent was obtained from the participant before sample collection. Detailed strain information is provided in Table S1. In addition, we analyzed 2,289 *E. coli* isolates from the NCBI database (https://www.ncbi.nlm.nih.gov), originating from 71 countries across six continents (excluding Antarctica), including China, the United States, Australia, Germany, Brazil, and South Africa. These isolates were derived from diverse hosts, such as humans, bovine, cats, dogs, swine, avian, and fish (Table S8). Map source: The map was generated using the amCharts Pixel Map Generator (https://pixelmap.amcharts.com) and is licensed under the Creative Commons Attribution‐NonCommercial 4.0 International License (Figure 1A).

This study was approved by the Animal Management and Ethics Committee of Huazhong Agricultural University (Approval No. HZAUCH‐2025‐0011), as bacterial isolates were obtained from postmortem animal tissues. All procedures related to the handling of such samples were performed in full compliance with the guidelines outlined in the “Technical Specification for the Harmless Treatment of Dead Animals” issued by the Ministry of Agriculture of the People's Republic of China (https://www.gov.cn/gongbao/content/2013/content_2547154.htm).

All strains were initially cultured on Luria Bertani (LB) agar plates, and single colonies were subsequently inoculated into LB broth and incubated at 37 °C with shaking at 180 rpm upon OD_600_ ≈ 0.6. Genomic DNA from all *E. coli* strains was extracted using a commercial kit (TIAN‐GEN, Beijing, China). DNA quality and concentration were evaluated via 1% agarose gel electrophoresis, NanoDrop 2000 spectrophotometry (Thermo Scientific, Waltham, MA, USA), and Qubit 4 fluorometry (Thermo Scientific, Waltham, USA).

For long‐read sequencing, Oxford Nanopore libraries were constructed using the SQK‐LSK109 kit and sequenced to ∼1 Gbp of data per sample. Next‐generation sequencing (NGS) comprised paired‐end sequencing, and at least 3 µg of genomic DNA was used for library construction. Paired‐end libraries with an insert size of approximately 400 bp were prepared following the standard genomic DNA library preparation protocol. Genomic DNA was sheared into smaller fragments of the desired size using a Covaris S220 (Woburn, MA USA), and blunt ends were generated with T4 DNA polymerase. After adding an ‘A’ base to the 3' end of the blunt phosphorylated DNA fragments, adapters were ligated to the ends. The desired fragments were purified by gel electrophoresis, followed by selective enrichment and amplification via PCR. The index tag was introduced into the adapter during the PCR amplification step. A library quality check was performed, and the libraries were subsequently sequenced using the MGI‐T7 platform (Shanghai BIOZERON Biotech Co. Ltd) in PE150 mode, according to standard protocols, generating ∼2 Gbp of data per sample.

### 4.2 Definition of a Modular Pan‐methylome Analysis Framework

To enable population‐scale comparison of bacterial methylomes, we established a modular pan‐methylome analysis framework that integrates long‐read methylation profiling with pan‐genomic architecture (Figure 1C). The framework is designed to address three key methodological challenges in bacterial epigenomics: defining a comparable methylation substrate across strains; representing methylation variation in a quantitative and standardized manner; and enabling population‐scale phyloepigenetic reconstruction, together with the adaptation of epigenome‐wide association and co‐methylation network analyses, to bacterial methylomes.

The framework consists of five interconnected analytical components. First, methylation systems are prioritized based on enzymatic encoding, population‐level conservation, and phylogenetic informativeness, defining a stable substrate for comparative analysis. Second, genome‐wide methylation signals are projected onto pan‐genome coordinates and partitioned by genomic context and strand orientation. Third, we adapt phyloepigenetic reconstruction to bacteria by building methylation‐based phylogenetic relationships directly from the pan‐methylome profile, thereby extending a concept previously established in eukaryotes that typically relies on array‐ or sequencing‐derived methylation levels to a bacterial setting with a distinct data structure and analytical framework. Fourth, quantitative methylation features are derived to enable standardized comparison across genes, non‐coding regions, and strains. Finally, the framework supports downstream population‐scale analyses, including epigenome‐wide association testing and co‐methylation association.

This modular design allows each component to be evaluated independently while maintaining a coherent analytical pipeline, providing a generalizable foundation for quantitative and comparative bacterial methylome analysis. Scripts for pan‐methylome construction, phyloepigenetic analysis, and EWAS are available on GitHub.

### 4.3 Genome Assembly, Pan‐genome Construction, and Phylogenetic Analysis

Raw Illumina short reads were quality‐checked using FastQC v0.11.9 (https://www.bioinformatics.babraham.ac.uk/projects/fastqc/) and trimmed with Trimmomatic v0.38.1 to remove adapters and low‐quality sequences [49]. Nanopore sequencing data were base‐called and demultiplexed by barcode using Guppy v1.1.

*E. coli* genomes were assembled using NECAT v0.0.1 and polished with NextPolish v1.4.1, incorporating Illumina short reads for error correction [50, 51]. Genome annotation was performed with Prokka v1.12, and the pan‐genome construction was carried out using Panaroo v1.3.4 [52, 53]. Here, we constructed two pan‐genomes: one based on the 84 *E. coli* strains sequenced in this study, and another based on 2,373 strains, incorporating an additional 2289 complete *E. coli* genomes from the NCBI database. Gene sequences of the 84 *E. coli* strains collected in this study were extracted using SeqKit v2.4.0, based on GFF annotation files generated by Prokka [52, 54]. To identify essential genes, we performed BLASTn v2.2.31 (identity > 90% and coverage > 80%) searches of previously reported essential genes, necessary for the growth of 18 *E. coli* strains in three environmental conditions [31], against the core genes of strain 2373, and matched genes were defined as essential. To identify *E. coli* virulence genes, the complete gene sequences of each strain were compared against the VFDB database using BLASTn (identity > 90% and coverage > 80%) [55]. A Maximum Likelihood (ML) phylogenetic tree was constructed using IQ‐TREE2 v1.6.1 with 1000 bootstrap replicates [56].

### 4.4 Criteria‐Based Selection of Methylation Systems for Population‐Scale Analysis

These steps collectively define a criteria‐based filtering strategy to identify methylation systems suitable for population‐scale comparative analysis. A total of 3636 MTase protein sequences were retrieved from the REBASE database and aligned to the genomes of the 84 *E. coli* strains using tBLASTn (identity > 90%, coverage > 80%). MTases were grouped according to their corresponding recognition motifs. Simulated genomes were generated using IQ‐TREE based on the topology of the core‐gene SNP phylogenetic tree and the corresponding optimized substitution model [57]. The occurrences of each recognition motif were quantified in both real and simulated genomes to assess phylogenetic signal. To evaluate the spatial heterogeneity of motif distribution, genomes were divided into non‐overlapping windows of 500, 1000, and 1500 bp, and the coefficient of variation (CV) of motif counts across windows was calculated.

Nanopore sequencing data were processed for methylation detection. Raw Fast5 files were demultiplexed using the “demux_fast5” function from the ONT ont_fast5_api toolkit, and methylation events were identified using Tombo v1.5.1 (https://github.com/nanoporetech/tombo). To minimize false positives and ensure robust downstream pan‐methylome analyses, only motifs identified by Tombo with a 100% estimated methylation fraction were retained. Methylation sites were classified based on the flanking motif sequence and corresponding MTase type. Sites detected in >80% of the strains were defined as core methylation sites, while the remaining were designated as accessory sites. To infer functional relevance, genes containing core methylation sites were annotated with Gene Ontology (GO) biological processes using the ClusterProfiler2 R package [58].

### 4.5 Markov Model‐Based Correction for k‐mer Frequency

To correct for sequence‐composition bias, we calculated observed‐to‐expected (O/E) ratios for Dam and Dcm methylation motifs in gene and non‐coding regions using zero‐order and first‐order Markov models [59]. Analyses were performed separately by strain, methyltransferase system, genomic region type, and strand orientation.

For each region category, the observed count was defined as the number of detected Dam or Dcm target motifs. Expected motif counts were estimated from the nucleotide composition of the corresponding sequences. In the zero‐order Markov model, the expected probability of a motif was calculated as the product of individual nucleotide frequencies:

$$P_{o}\;\left(b_{1}b_{2}\text{…}b_{k}\right)=\prod_{i}^{k}P\left(b_{i}\right)$$

In the first‐order Markov model, expected motif probabilities were calculated using nucleotide transition probabilities:

$$P_{o}\;\left(b_{1}b_{2}\text{…}b_{k}\right)=P\left(b_{i}\right)\prod_{i=2}^{k}P\left(b_{i}|b_{i-1}\right)$$

Expected motif counts were obtained by multiplying motif probabilities by the number of possible k‐mer positions in the analyzed sequences. The O/E ratio was then calculated as:

$$O/E=\frac{O}{E}$$

where O represents the observed motif count and E represents the expected motif count estimated from the corresponding Markov model.

### 4.6 Pan‐methylome Profile Construction

Genes present in at least 10 of the 84 *E. coli* strains were selected for downstream analysis. For each strain, corresponding gene sequences were retrieved by BLAST alignment against a reference gene set and compiled into FASTA files, and non‐coding regions were extracted based on genomic coordinates. Gene sequences were aligned using MAFFT v7.505 codon alignment implemented in PhyloSuite v1.2.3, whereas non‐coding regions were aligned using MAFFT with the “–globalpair” option [60, 61]. Methylation information was then mapped onto the aligned sequences and classified by transcriptional strand as sense or antisense methylation (Figure S21). Methylation detected on both strands of a given motif was defined as complete methylation, whereas methylation restricted to a single strand was classified as sense or antisense hemi‐methylation. The resulting pan‐methylome profile records gene identity, methylation position, strain coverage, motif frequency, methylation frequency, and strain distribution.

### 4.7 Phyloepigenetic Tree Construction

To construct the phyloepigenetic tree, we first created a correlation‐based dissimilarity matrix using the core genes of the 84 *E. coli* strains. The dissimilarity matrix was defined as 1 – ρ, where ρ is the Pearson correlation between strains [3]. This approach is theoretically justified by previous research, which shows that in a phylogeny of continuous traits, the trait variance in a species equals the sum of the branch lengths from the species to the root, while the covariance between two species corresponds to the sum of their shared ancestral branch lengths [62]. This approach avoids confounding by sequence divergence and enables direct comparison of continuous methylation traits across strains.

We constructed eight correlation‐based dissimilarity matrices, including gene sense and antisense methylation for both Dam and Dcm in gene and non‐coding regions. These matrices were then combined into a single *E. coli* methylation dissimilarity matrix, following mean‐based standardization. Finally, the phyloepigenetic tree was constructed using hierarchical clustering with average linkage.

### 4.8 Quantitative Representation of Pan‐methylome Features

For the quantitative analysis of *E. coli* methylation, we introduced three indicators to systematically compare methylation levels between gene and non‐coding regions: methylation per kilobase (MPK), methylation ratio (MR), and motif frequency ratio (MFR). The detailed calculations are provided below:

$$MPK=\frac{Methylation}{\sqrt{Gene\;Length}}\;\times 10^{3}$$

$$MR=\frac{Methylation}{Motif}\;\times 10^{2}$$

$$MFR=\frac{Motif}{Sequence\;Number}\;\times 10^{2}$$

Methylation refers to the number of methylation sites within a gene or non‐coding region. Gene length refers to the length of the gene or non‐coding region in base pairs. Motif refers to the number of motifs present in the gene or non‐coding region. The sequence number represents the number of strains that contain the gene or non‐coding region. Furthermore, to mitigate the influence of gene length on the final result, the gene length of MPK was square‐root transformed. Here, MPK denotes methylation per unit length, MR denotes methylation per motif, and MFR denotes motif stability across multiple strains.

### 4.9 Identification of Gene Essentiality by CRISPRi Knockdown

To assess gene essentiality using CRISPRi, we first prepared electrocompetent *E. coli* MG1655 [63]. A single colony was inoculated into 50 mL LB broth and incubated at 37°C with shaking at 180 rpm for approximately 2 h, until it became slightly turbid (OD_600_ = 0.4). Cells were collected by centrifugation at 4°C, 4000 g for 10 min, and the pellet was washed once with 5 mL ice‐cold sterile ddH_2_O, following incubation on ice for 30 min and another centrifugation step. The final pellet was resuspended in 2 mL of pre‐chilled 10% glycerol, aliquoted (150 µL/tube), and stored at −80°C.

For construction of recombinant CRISPRi plasmids, sgRNA sequences targeting specific genes were designed and amplified using primers listed in Table S9. The pdCas9‐gRNA‐Cm plasmid was linearized by double digestion with SacII and SalI, followed by insertion of the sgRNA fragment through homologous recombination. Recombinant plasmids were transformed into *E. coli* DH5α competent cells, plated on chloramphenicol‐containing LB agar (25 µg/mL), and verified by colony PCR and Sanger sequencing. Correct constructs were retained for downstream use.

For gene knockdown, the validated pdCas9‐gRNA plasmids were electroporated into *E. coli* MG1655 with an empty pdCas9 vector used as a negative control. Transformants were cultured overnight in LB medium containing chloramphenicol at 37°C with shaking. The next day, the cultures were diluted 1:100 into fresh medium and grown to OD_600_ ≈ 0.6, 100 µL of culture was serially diluted, and 5 µL of each dilution was spotted onto LA agar plates supplemented with 1 µm anhydrotetracycline (aTc). Plates were incubated at 37°C overnight, and bacterial growth was monitored to assess gene repression efficiency.

### 4.10 Consensus Co‐methylation Network Construction and Analysis

Four consensus co‐methylation networks: Dam gene, Dam non‐coding, Dcm gene, and Dcm non‐coding, were constructed. Each network was built based on three topological overlap matrices (complete methylation, sense hemi‐methylation, and antisense hemi‐methylation). To enhance the co‐methylation effect, an adjacency matrix was generated by averaging the MPK and MR indices across each dataset. The soft threshold power for the adjacency matrix was selected based on the mean connectivity of the network, with a power of 6 for the Dam/Dcm gene network and 12 for the Dam/Dcm non‐coding network. The result was converted into a topological overlap matrix (TOM), and its dissimilarity (1‐TOM) was used for hierarchical clustering. The trees were trimmed using the R function “cutreeDynamic()” to assign modules containing at least 20 genes. The module colors in both networks were determined using the “matchLabels()” function from the WGCNA package [64].

In the co‐methylation network analysis, we first compared the correlation between TOM values, representing the co‐methylation profile of genes, and gene associations. Data from all seven *E. coli* strains, including CFT073, EDL933, IAI1, IAI39, MG1655, Sakai, and UMN026, were retrieved from the STRING database [46]. Gene interaction pairs with scores > 700 were considered high‐confidence, while those ≤ 700 were categorized as low‐confidence. The genes of each strain were then matched to the co‐methylation network using BLASTn, with a 90% identity and 80% coverage threshold. Statistical comparisons of TOM values between high‐ and low‐confidence gene pairs were performed using two‐sided Student's *t*‐tests. For functional annotation, we used ClusterProfile2 to annotate the GO biological processes of the modular genes in each co‐methylation module [58]. Biological processes were then grouped under parent ontology terms using the “rrvgo” package [65]. Additionally, co‐methylation network modules were visualized in Cytoscape and clustered using the spring‐embedded layout, a force‐directed algorithm based on TOM values [66, 67].

### 4.11 Epigenome‐Wide Association Analysis (EWAS) of Environmental Tolerance

To assess environmental tolerance, this study evaluated the growth performance of 84 *E. coli* strains under five environmental conditions: control (37°C, pH = 7), high temperature (45°C, pH = 7), low temperature (25°C, pH = 7), acidic (37°C, pH = 5), and alkaline (37°C, pH = 9). Strains were initially cultured overnight in LB medium, then inoculated into the respective test media at a 1:100 dilution with an initial OD_600_ of 0.01. A total of 200 µL of bacterial suspension was transferred into honeycomb plates, with three technical replicates per strain. For temperature tolerance assays, cultures were incubated at 25°C, 37°C and 45°C, while for acid‐base tolerance tests, LB media adjusted to pH = 5, pH = 7, and pH = 9 were used at a constant temperature of 37°C. OD_600_ values were recorded automatically every 2 h, and growth curves were generated upon completion of the measurements.

For the EWAS analysis, environmentally tolerant and intolerant strains were first distinguished based on paired Student's *t*‐tests. Subsequently, the methylation information of each strain was transformed. Specifically, Dam complete methylation was encoded as AMMA, while Dam hemi‐methylation was represented as AMAA or AAMA. Similarly, Dcm methylation was denoted as CNNC, and Dcm hemimethylation as CNCC or CCNC. This encoding scheme represents methylation states as ordered categorical variables, enabling differentiation between complete and hemi‐methylation states and facilitating trend‐based association testing. Based on these encoding rules, we employed PLINK with the “TREND” model to perform the EWAS analysis [68]. Temperature tolerance‐related and acid‐base tolerance‐related methylation sites were identified by intersecting the methylation sites associated with different environmental tolerances. Methylation sites selected for experimental validation were chosen based on the lowest association *P*‐values.

### 4.12 Methylation Site Knockout via Synonymous Substitution

To knock out target methylation motifs while preserving the amino acid sequence of the target gene, synonymous substitutions were introduced. The target DNA fragment was synthesized, and the construction of the mutant strains followed previously described methods [69]. The synthetic fragment sequences and primers used are listed in Tables S10 and S11. The pRE112‐derived plasmids were transformed into *E. coli* χ7213 competent cells. These competent cells served as the donor strain for conjugation, and were mixed with the recipient strain at a 10:1 ratio. The mixture was spotted onto a sterile filter membrane (0.22 µm) and placed on LB agar plates containing 50 µg/mL diaminopimelic acid (DAP) (Aladdin, Shanghai, China). After 5 h of incubation at 37°C, the surface cells were washed off, and those remaining were plated onto LB agar plates containing chloramphenicol (25 µg/mL) for overnight growth at 37°C. Colonies were picked, and PCR used to identify single‐crossover strains. Double‐crossover mutants were selected using LB agar plates supplemented with 10% sucrose, PCR was used to verify the presence of the target gene.

### 4.13 Statistical Analysis

All statistical analyses were performed using R (version 4.3.1), Python (version 3.11). Data preprocessing, including quality filtering, normalization, and feature selection, was conducted as described in the corresponding methodological sections. For methylome analyses, only high‐confidence methylation sites that passed the predefined quality thresholds were retained for downstream analyses. The sample size (n) for each experiment or analysis is provided in the corresponding figure legends or methodological descriptions.

Comparisons between two groups were performed using two‐tailed Student's *t*‐tests when data met the assumptions of normality and homogeneity of variance. Comparisons among multiple groups were conducted using one‐way Welch's analysis of variance (ANOVA), unless otherwise stated. For non‐parametric data, appropriate Mann–Whitney U test were applied. Correlations were assessed using Pearson correlation coefficient unless otherwise specified. For genome‐wide and methylome‐wide analyses involving multiple comparisons, *p* values were adjusted using the Benjamini–Hochberg false discovery rate (FDR) correction where applicable. Statistical significance was defined as a two‐sided *p* value < 0.05 unless otherwise indicated.

The specific statistical methods used for individual analyses are described in the corresponding figure legends and methodological sections.

## Author Contributions

**Mingyang Wang**: data curation, visualization. **Ran Zhuo**: data curation, formal analysis. **Yanxi Wan**: methodology, software, formal analysis. **Bin Ma**: writing – original draft, writing – review and editing, software, methodology, conceptualization, data curation, visualization, investigation. **Xinrun Li**: data curation, validation. **Aohan Guan**: formal analysis. **Zahra Zeinalzadeh**: software, validation. **Huimin Gong**: writing – original draft, conceptualization, resources. **Wanting Chen**: formal analysis. **Qi Huang**: project administration, supervision. **Zaixiao Rao**: methodology, writing – original draft. **Sihua Zhang**: resources, project administration. **Yuncai Xiao**: supervision, resources. **Xiliang Wang**: project administration. **Yuan Gao**: software, formal analysis. **Rui Zhou**: funding acquisition, project administration, resources. **Hongbo Zhou**: writing – review and editing, funding acquisition. **Paul R. Langford**: writing – review and editing, funding acquisition. **Yun Wan**: resources. **Hui Jin**: writing – review and editing, resources, project administration, funding acquisition, conceptualization. **Xiangru Wang**: resources, project administration. **Chengliang Zhu**: writing – review and editing, funding acquisition, conceptualization, project administration, resources. **Rui Luo**: writing – review and editing, funding acquisition.

## Conflicts of Interest

The authors declare no conflicts of interest.

## Supporting information




**Supporting File 1**: advs76559‐sup‐0001‐SuppMatfiguresS1‐S21.zip

**Supporting File 2**: advs76559‐sup‐0002‐SuppMatfigurescaptions.docx.

**Supporting File 3**: advs76559‐sup‐0003‐SuppMatTablesS1‐S11.zip.

## Data Availability

Whole‐genome sequencing data for the *E. coli* isolates analyzed in this study are publicly available in the GenBank database under BioProject accession number PRJNA1295596. The simulated sequences analyzed in this study are publicly available on GitHub: https://github.com/Bin‐Ma/Pan‐Methylome/data. The primary FAST5 output files from nanopore sequencing are available upon request but have not been uploaded to a public repository due to their large size (>1.5 Tb). Requests for these materials should be directed to Hui Jin at jinhui@mail.hzau.edu.cn.
