# Supplementary material for: A Pan‐Methylome Framework for Population‐Scale Bacterial Epigenomics
Source: Adv Sci (Weinh). 2026 Jul 13;13(55):e76559. doi: 10.1002/advs.76559 (PMC13360123; doi:10.1002/advs.76559)
Supplement: Supplementary file 2 [file ADVS-13-e76559-s002.zip › TableS11.docx]

**Supplemental Table 11. Primers for targeted knockout of methylation sites.**

| Primer | Sequence |
| --- | --- |
| HAIA13-pRE112-F | ACTGCATGAATTCCCGGGAGAGCTCGAAGATGGCTAACCAGGCGAA |
| HAIA13-UP-R | ACTAAAGAACCAGCATTCTGCATATTCGGGATGTCCATAAAATG |
| HAIA13-F | ATGCAGAATGCTGGTTCT |
| HAIA13-R | ATTGTAGCCAAGCGTTAACGTTGACGGTCCA |
| HAIA13-spc-F | TCAACGTTAACGCTTGGCTACAATGAGGAGGATATATTTGAATA |
| spc-HAIA13-DOWN-R | CATCTTTCATTGTAGCCAAGCGTTATAATTTTTTTAATCTGTTATTTA |
| HAIA13-DOWN-F | ATTATAACGCTTGGCTACAATGAAAGAT |
| HAIA13-pRE112-R | GATCCCAAGCTTCTTCTAGAGGTACCATGTGATAACAGTTGGCGCAT |
| HAIA13-pRE112-F | ACTGCATGAATTCCCGGGAGAGCTCGAAGATGGCTAACCAGGCGAA |
| HAIA13-pRE112-R | GATCCCAAGCTTCTTCTAGAGGTACCATGTGATAACAGTTGGCGCAT |
| HAIA13-JCQS-F | AGCTTGGCTATAACCTGGT |
| HAIA13-upchange-R | TCGATATTGTCGGTAGCTAA |
| HAIA13-downchange-F | TGGTACCGTGGAATCATC |
| HAIA13-JCQS-R | CTGGTAAACGCCAACAG |
