# Supplementary material for: A Pan‐Methylome Framework for Population‐Scale Bacterial Epigenomics
Source: Adv Sci (Weinh). 2026 Jul 13;13(55):e76559. doi: 10.1002/advs.76559 (PMC13360123; doi:10.1002/advs.76559)
Supplement: Supplementary file 2 [file ADVS-13-e76559-s002.zip › TableS1.docx]

**Supplemental Table 1. Sampling information of 84 *Escherichia coli* isolates.**

| Strain | Geographic location | Collection date | Host | epi-phylogroup | |
| --- | --- | --- | --- | --- | --- |
| DL10 | Liaoning | 2019.8.31 | Avian | | A |
| FJA5 | Fujian | 2020.8.19 | Avian | | A |
| GDA1 | Guangdong | 2020.7.15 | Avian | | B |
| GDA10 | Guangdong | 2020.7.15 | Avian | | A |
| GDA13 | Guangdong | 2020.7.15 | Avian | | A |
| GDA17 | Guangdong | 2020.7.15 | Avian | | B |
| GDA23 | Guangdong | 2020.7.15 | Avian | | A |
| GDA25 | Guangdong | 2020.7.15 | Avian | | C |
| GDA4 | Guangdong | 2020.7.15 | Avian | | B |
| GDA9 | Guangdong | 2020.7.15 | Avian | | B |
| GDB10 | Guangdong | 2020.7.21 | Avian | | B |
| GDB12 | Guangdong | 2020.7.21 | Avian | | B |
| GDB15 | Guangdong | 2020.7.21 | Avian | | A |
| GDB16 | Guangdong | 2020.7.21 | Avian | | A |
| GDC19 | Guangdong | 2020.7.29 | Avian | | A |
| GDC2 | Guangdong | 2020.7.29 | Avian | | A |
| GDC22 | Guangdong | 2020.7.29 | Avian | | A |
| GDC3 | Guangdong | 2020.7.29 | Avian | | A |
| GDD2 | Guangdong | 2020.7.30 | Avian | | A |
| GDD3 | Guangdong | 2020.7.30 | Avian | | A |
| GDD4 | Guangdong | 2020.7.30 | Avian | | C |
| GDD5 | Guangdong | 2020.7.30 | Avian | | B |
| GDE22 | Guangdong | 2020.8.7 | Avian | | A |
| GDE3 | Guangdong | 2020.8.7 | Avian | | A |
| GDE4 | Guangdong | 2020.8.7 | Avian | | A |
| GDG10 | Guangdong | 2020.8.24 | Avian | | B |
| GDG15 | Guangdong | 2020.8.24 | Avian | | C |
| GDH3 | Guangdong | 2020.8.31 | Avian | | B |
| GDI10 | Guangdong | 2020.7.16 | Avian | | A |
| GDI22 | Guangdong | 2020.7.16 | Avian | | A |
| GDI28 | Guangdong | 2020.7.16 | Avian | | B |
| GDJ12 | Guangdong | 2020.9.25 | Avian | | A |
| GDJ13 | Guangdong | 2020.9.25 | Avian | | A |
| GDK5 | Guangdong | 2020.9.27 | Avian | | A |
| GDK6 | Guangdong | 2020.9.27 | Avian | | B |
| GDK7 | Guangdong | 2020.9.27 | Avian | | A |
| GDK8 | Guangdong | 2020.9.27 | Avian | | A |
| GXB10 | Guangxi | 2020.7.22 | Avian | | B |
| GXB4 | Guangxi | 2020.7.22 | Avian | | A |
| GXB5 | Guangxi | 2020.7.22 | Avian | | A |
| GXC2 | Guangxi | 2020.8.25 | Avian | | B |
| GXC3 | Guangxi | 2020.8.25 | Avian | | A |
| HAIA13 | Hainan | 2020.8.26 | Avian | | A |
| HAIA14 | Hainan | 2020.8.26 | Avian | | A |
| HAIB2 | Hainan | 2020.8.31 | Avian | | B |
| HAIC2 | Hainan | 2020.9.4 | Avian | | A |
| HAID2 | Hainan | 2020.9.25 | Avian | | A |
| HAID6 | Hainan | 2020.9.25 | Avian | | A |
| HAID7 | Hainan | 2020.9.25 | Avian | | A |
| HNA2 | Hunan | 2020.7.6 | Avian | | B |
| HNB7 | Hunan | 2020.7.16 | Avian | | B |
| HNC11 | Hunan | 2020.8.24 | Avian | | B |
| HND1 | Hunan | 2020.11.19 | Avian | | B |
| JS94 | Jiangsu | 2019.8.16 | Avian | | B |
| JSA12 | Jiangsu | 2020.7.20 | Avian | | B |
| JSA8 | Jiangsu | 2020.7.20 | Avian | | B |
| LN27 | Liaoning | 2019.8.31 | Avian | | B |
| SCA2 | Sichuan | 2020.9.17 | Avian | | A |
| SCA4 | Sichuan | 2020.9.17 | Avian | | C |
| SD28 | Shandong | 2019.8.12 | Avian | | A |
| SDA1 | Shandong | 2020.7.6 | Avian | | C |
| SDC2 | Shandong | 2020.11.24 | Avian | | B |
| YNB2 | Yunnan | 2020.10.26 | Avian | | B |
| XT95 | Hubei | 2018.10.1-2019.9.30 | Human | | C |
| BJ25 | Beijing | 2018.10.1-2019.9.30 | Porcine | | A |
| FY215 | Henan | 2018.10.1-2019.9.30 | Porcine | | C |
| FY240 | Henan | 2018.10.1-2019.9.30 | Porcine | | C |
| GD40 | Guangdong | 2018.10.1-2019.9.30 | Porcine | | C |
| GS207 | Gansu | 2018.10.1-2019.9.30 | Porcine | | C |
| GS208 | Gansu | 2018.10.1-2019.9.30 | Porcine | | C |
| GZ12 | Guizhou | 2018.10.1-2019.9.30 | Porcine | | A |
| HUBJ12 | Hubei | 2018.10.1-2019.9.30 | Porcine | | C |
| HUBS37 | Hubei | 2018.10.1-2019.9.30 | Porcine | | C |
| HUN9 | Hunan | 2018.10.1-2019.9.30 | Porcine | | C |
| LN43 | Liaoning | 2018.10.1-2019.9.30 | Porcine | | C |
| NMG10 | Inner Mongolia | 2018.10.1-2019.9.30 | Porcine | | C |
| S104 | Henan | 2018.10.1-2019.9.30 | Porcine | | C |
| S77 | Henan | 2018.10.1-2019.9.30 | Porcine | | C |
| SAX35 | Shanxi | 2018.10.1-2019.9.30 | Porcine | | C |
| SD5 | Henan | 2018.10.1-2019.9.30 | Porcine | | C |
| SH56 | Shanghai | 2018.10.1-2019.9.30 | Porcine | | C |
| XD1 | Henan | 2018.10.1-2019.9.30 | Porcine | | C |
| XJ17 | Xinjiang | 2018.10.1-2019.9.30 | Porcine | | A |
| XJ40 | Xinjiang | 2018.10.1-2019.9.30 | Porcine | | C |
