# Supplementary material for: A Pan‐Methylome Framework for Population‐Scale Bacterial Epigenomics
Source: Adv Sci (Weinh). 2026 Jul 13;13(55):e76559. doi: 10.1002/advs.76559 (PMC13360123; doi:10.1002/advs.76559)
Supplement: Supplementary file 2 [file ADVS-13-e76559-s002.zip › TableS10.docx]

**Supplemental Table 10. DNA fragments designed by synonymous codon shuffling.**

| Gene | Sequence |
| --- | --- |
| HAIA13 | ATGCAGAATGCTGGTTCTTTAGTCGTCTTAGGTTCTATCAACGCCGACCATATCTTAAACTTACAGTCCTTCCCCACCCCGGGTGAGACTGTTACTGGCAATCATTACCAAGTCGCTTTCGGTGGTAAGGGTGCTAACCAAGCCGTTGCCGCCGGTCGCTCTGGCGCTAACATTGCTTTCATCGCTTGCACTGGCGACGATTCTATCGGCGAATCTGTCCGTCAACAATTAGCTACCGACAATATCGACATCACCCCTGTTTCTGTGATCAAGGGTGAGTCTACTGGCGTTGCTTTAATCTTCGTCAACGGTGAGGGCGAAAACGTTATTGGCATCCACGCTGGTGCCAACGCCGCTTTATCTCCTGCTTTAGTTGAGGCTCAGCGCGAACGCATCGCTAATGCTTCTGCTTTGTTGATGCAATTAGAGTCTCCTTTAGAGTCTGTTATGGCTGCTGCTAAGATTGCTCACCAGAACAAAACCATTGTCGCTTTAAATCCTGCCCCTGCCCGTGAGTTACCCGATGAGTTATTAGCTTTAGTTGATATCATCACTCCTAATGAGACTGAGGCTGAGAAATTAACTGGCATCCGCGTCGAGAACGACGAGGACGCTGCTAAAGCTGCTCAAGTTTTACACGAGAAGGGCATTCGCACCGTTTTAATCACCTTGGGTTCTCGCGGCGTTTGGGCTAGTGTTAACGGCGAGGGCCAACGTGTCCCTGGTTTTCGTGTTCAAGCCGTTGACACTATCGCCGCTGGTGACACTTTCAATGGTGCTTTGATTACTGCTTTATTAGAGGAGAAGCCTTTACCTGAAGCTATCCGTTTCGCTCACGCCGCTGCCGCTATCGCTGTTACTCGCAAGGGTGCTCAGCCCTCTGTTCCTTGGCGCGAGGAGATCGATGCTTTCTTGGACCGTCAACGTTAA |
