# Supplementary material for: A Pan‐Methylome Framework for Population‐Scale Bacterial Epigenomics
Source: Adv Sci (Weinh). 2026 Jul 13:e76559. Online ahead of print. doi: 10.1002/advs.76559 (PMC13360123; doi:10.1002/advs.76559)
Supplement: Supplementary file 1 — Supporting File 1: advs76559‐sup‐0001‐SuppMatfiguresS1‐S21.zip [file ADVS-9999-e76559-s003.zip › S2.pdf]

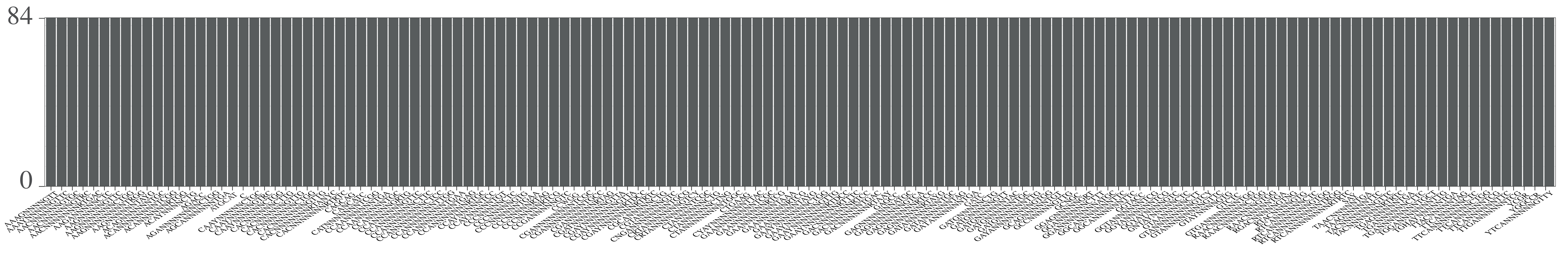
84
0
