# Supplementary figures and images for: A Pan‐Methylome Framework for Population‐Scale Bacterial Epigenomics
Source: Adv Sci (Weinh). 2026 Jul 13:e76559. Online ahead of print. doi: 10.1002/advs.76559 (PMC13360123; doi:10.1002/advs.76559)

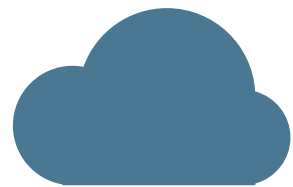

= RNA polymerases

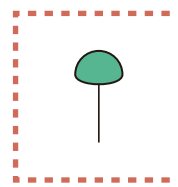

= Sense methylation

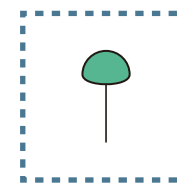

= Antisense methylation

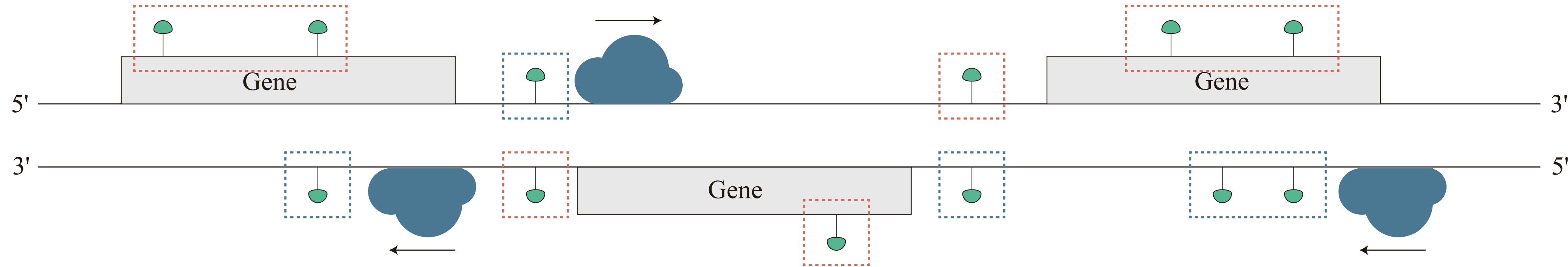

Supplement: Supplementary file 1 — Supporting File 1: advs76559‐sup‐0001‐SuppMatfiguresS1‐S21.zip [file ADVS-9999-e76559-s003.zip › S21.pdf]

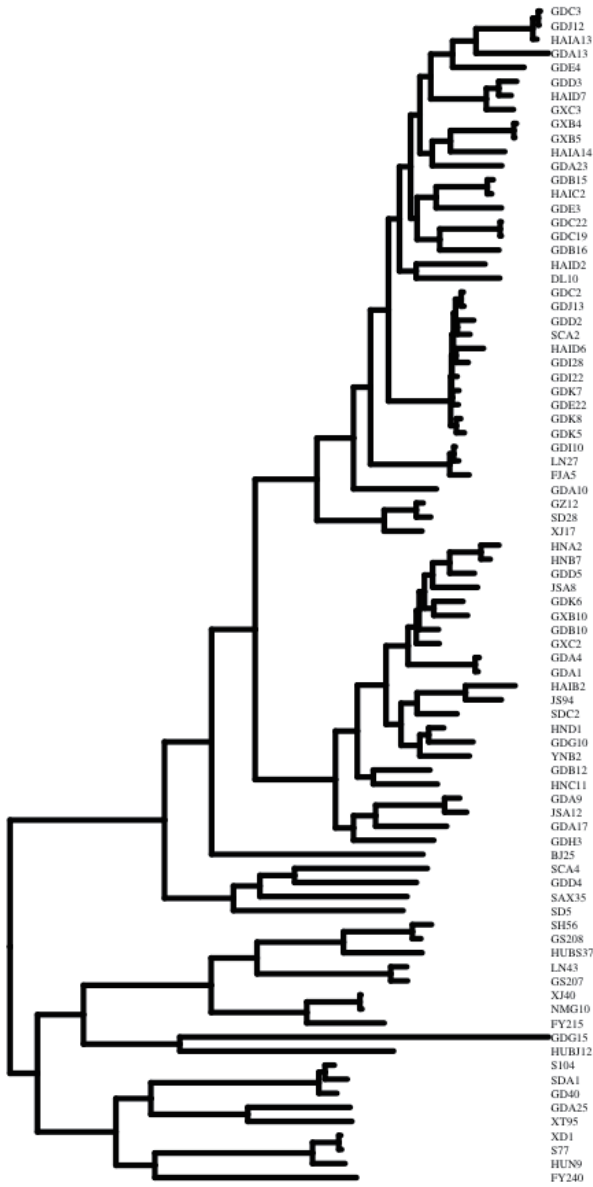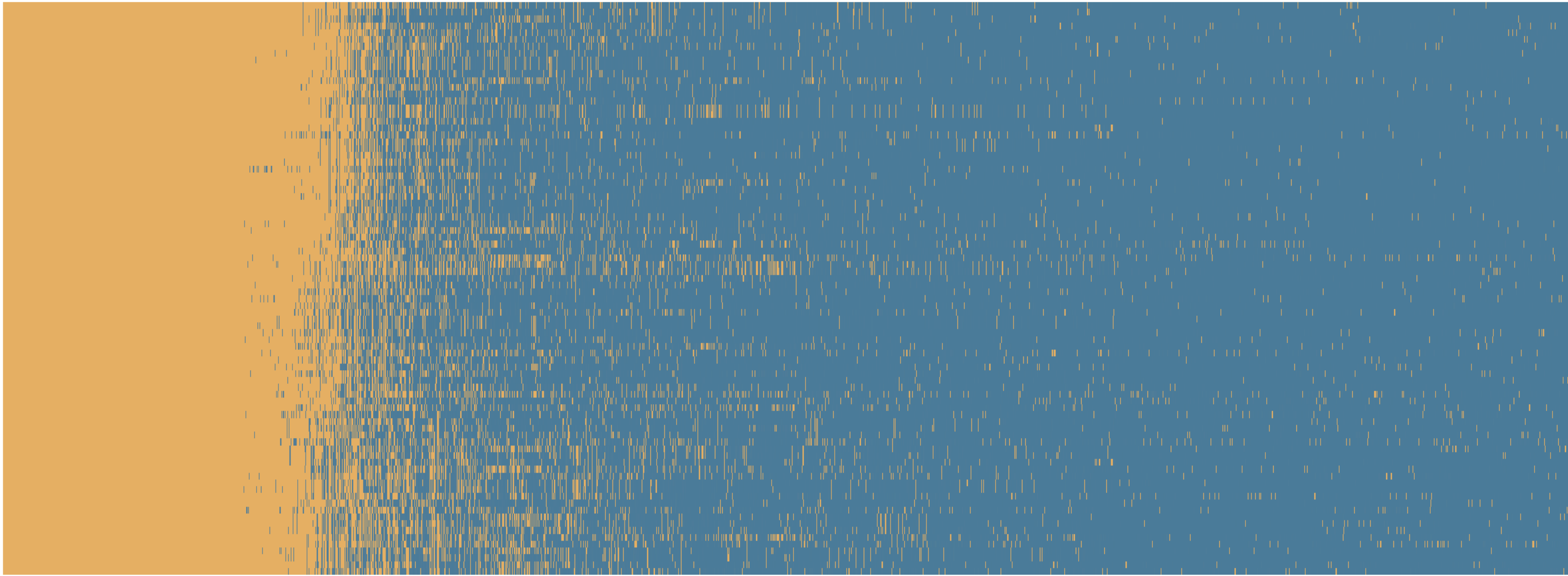

Supplement: Supplementary file 1 — Supporting File 1: advs76559‐sup‐0001‐SuppMatfiguresS1‐S21.zip [file ADVS-9999-e76559-s003.zip › S1.pdf]

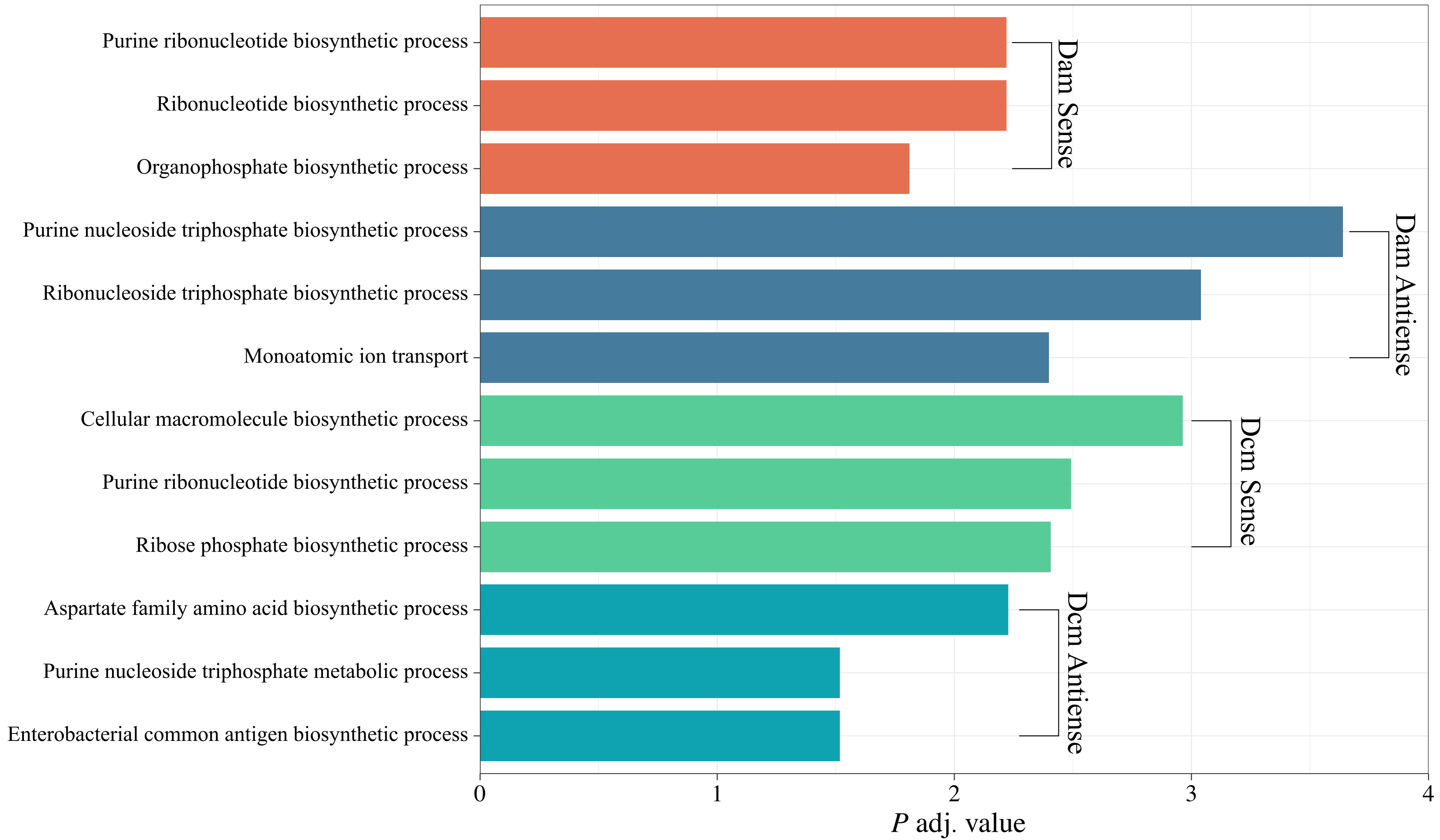

Supplement: Supplementary file 1 — Supporting File 1: advs76559‐sup‐0001‐SuppMatfiguresS1‐S21.zip [file ADVS-9999-e76559-s003.zip › S3.pdf]

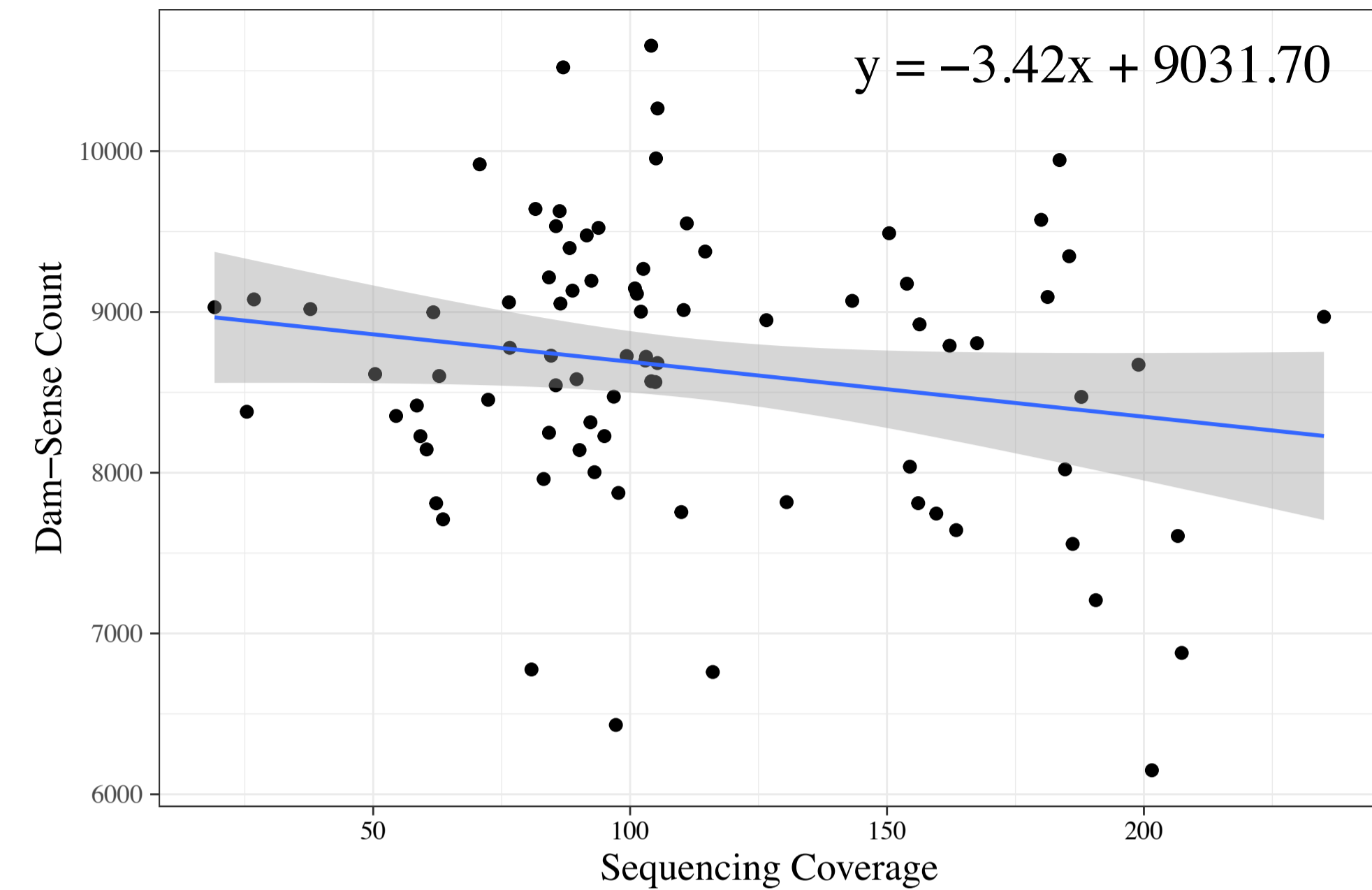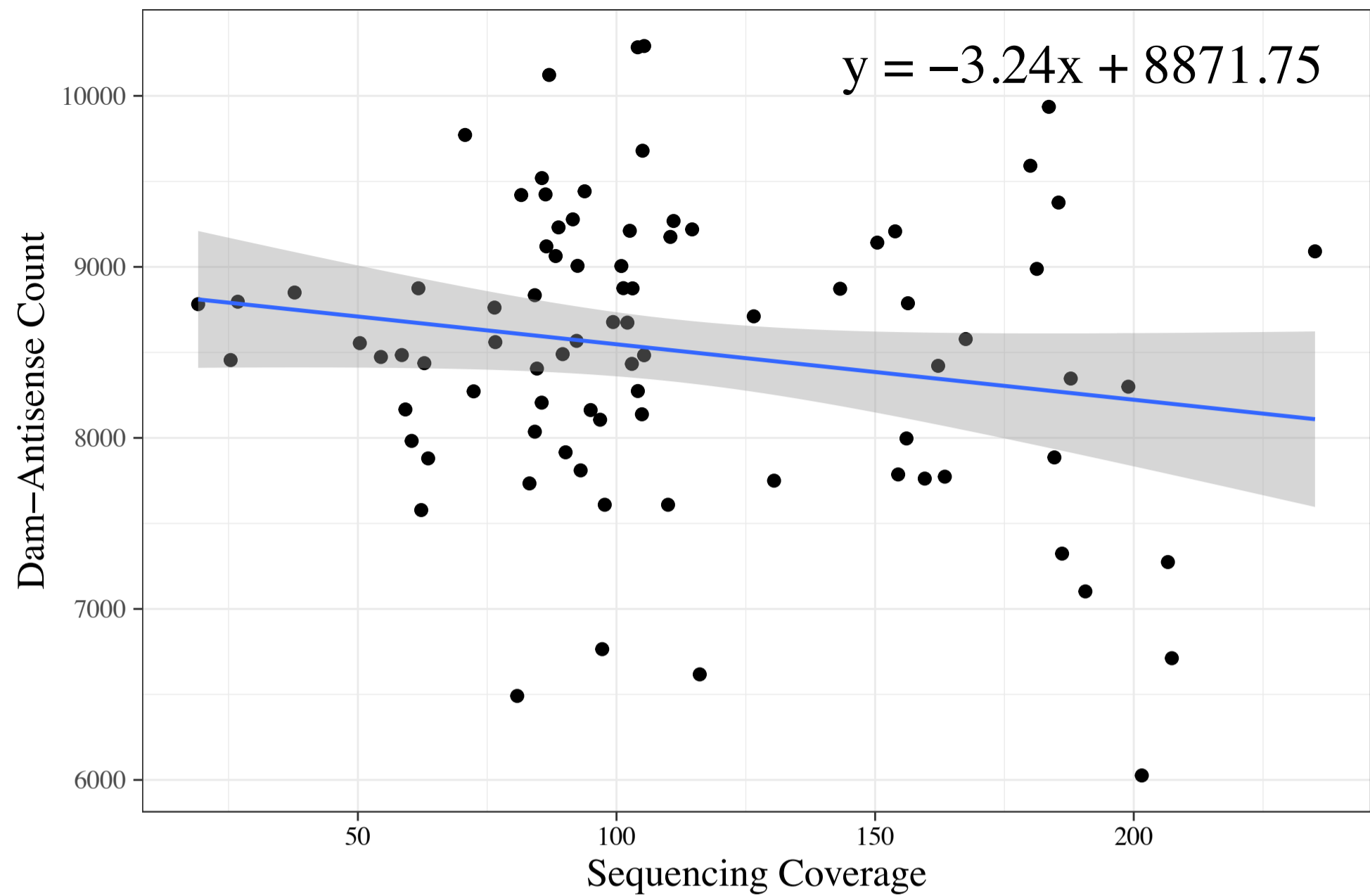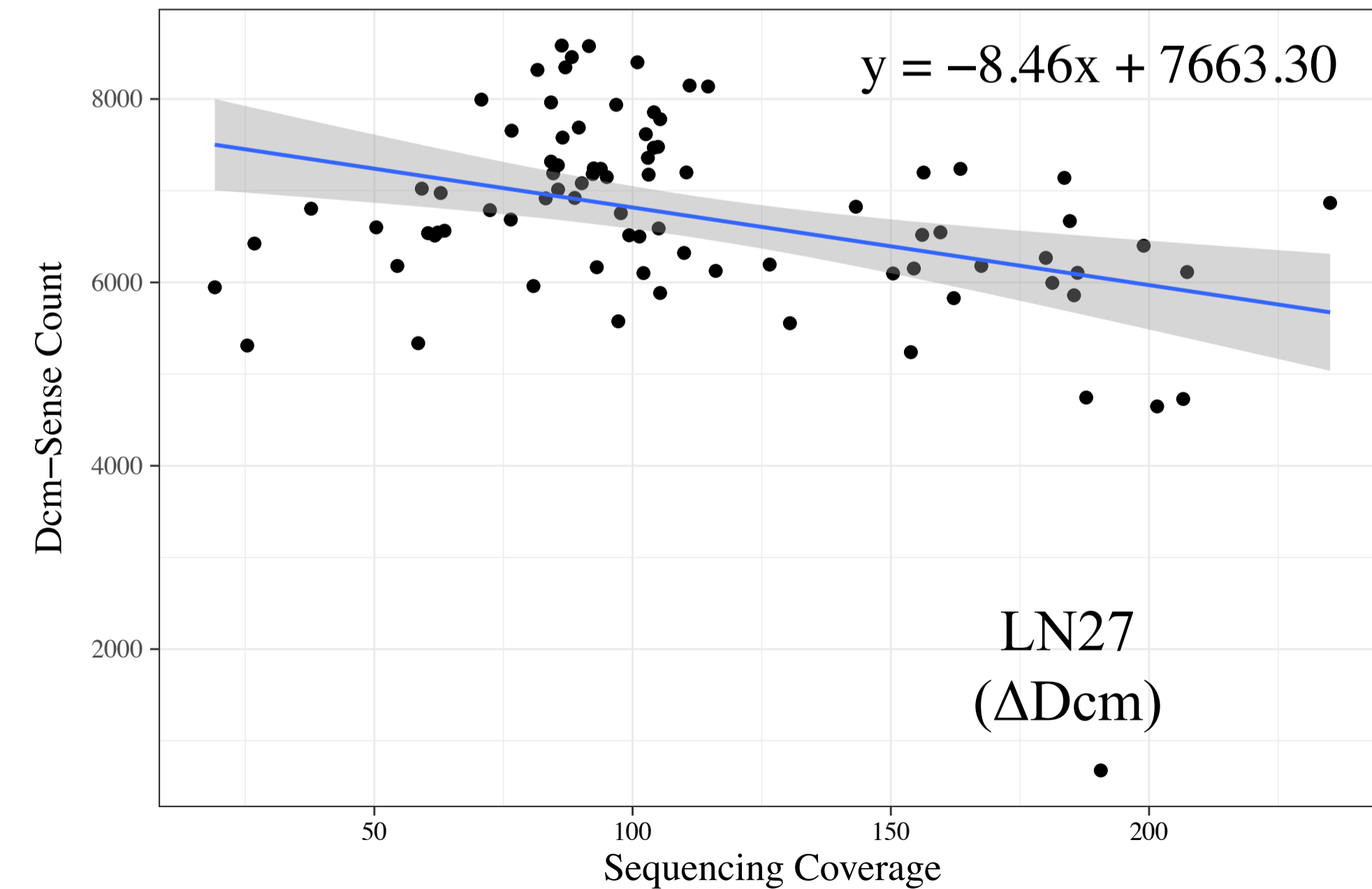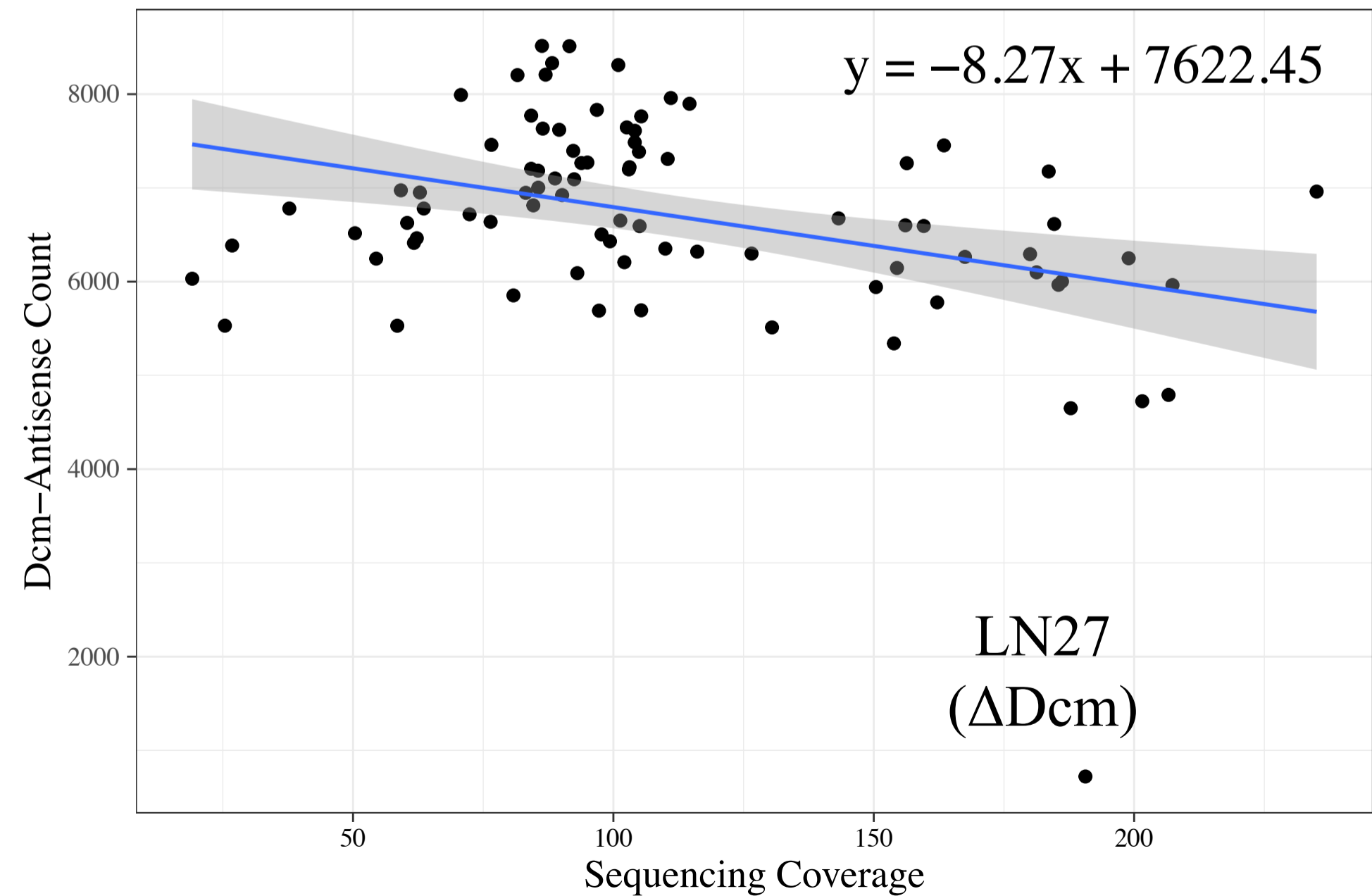

Supplement: Supplementary file 1 — Supporting File 1: advs76559‐sup‐0001‐SuppMatfiguresS1‐S21.zip [file ADVS-9999-e76559-s003.zip › S4.pdf]

A

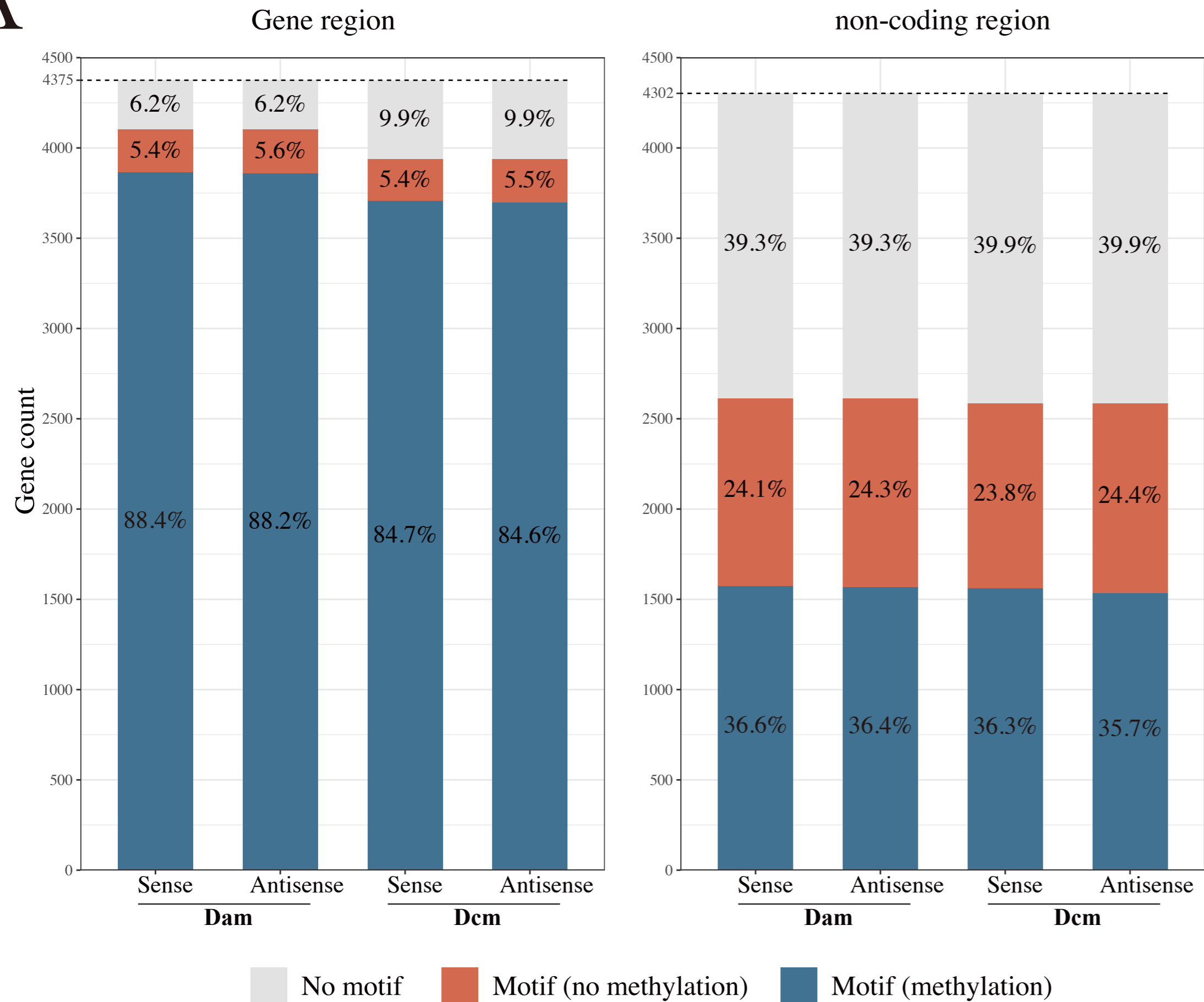

B

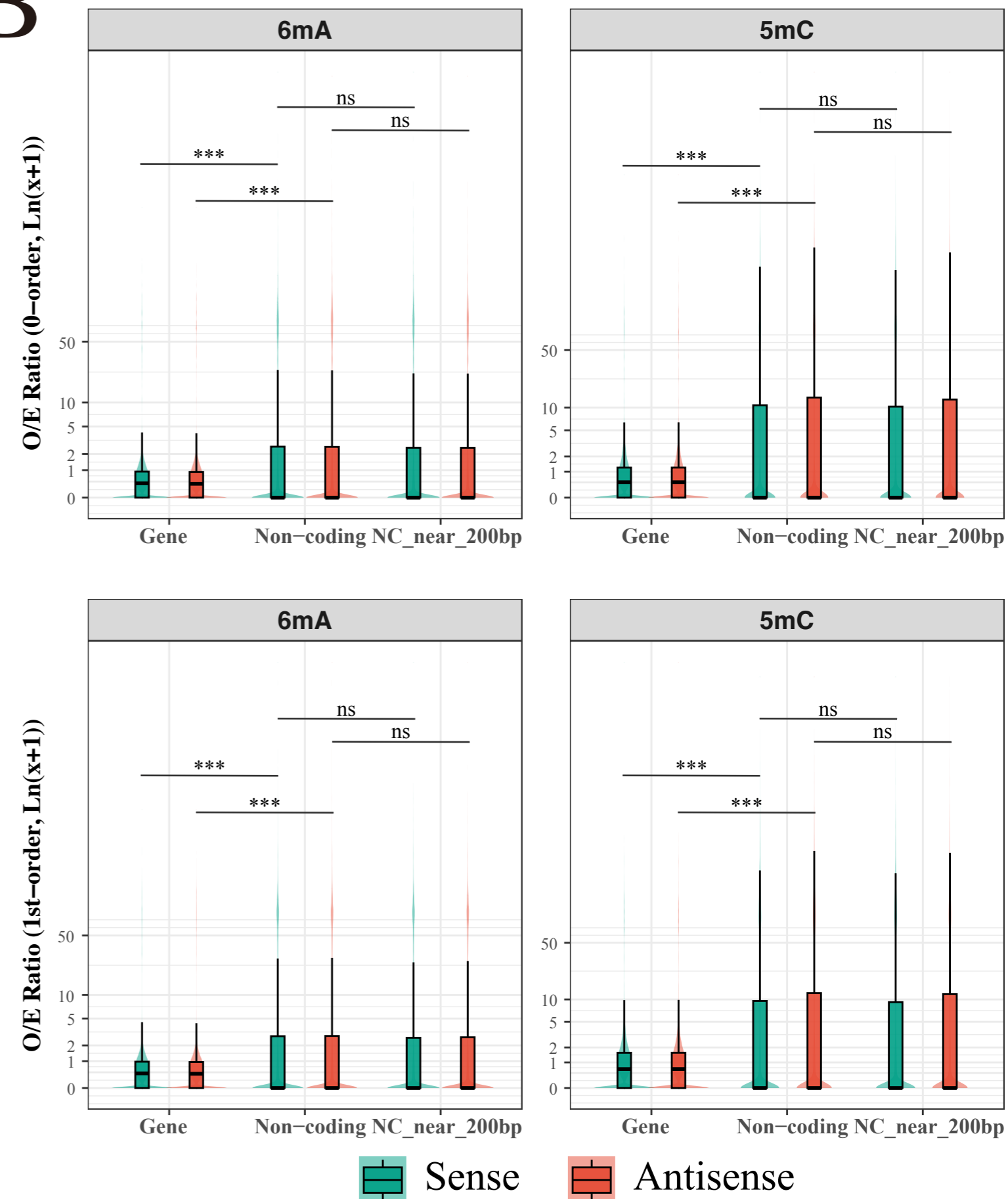

Supplement: Supplementary file 1 — Supporting File 1: advs76559‐sup‐0001‐SuppMatfiguresS1‐S21.zip [file ADVS-9999-e76559-s003.zip › S5.pdf]

A

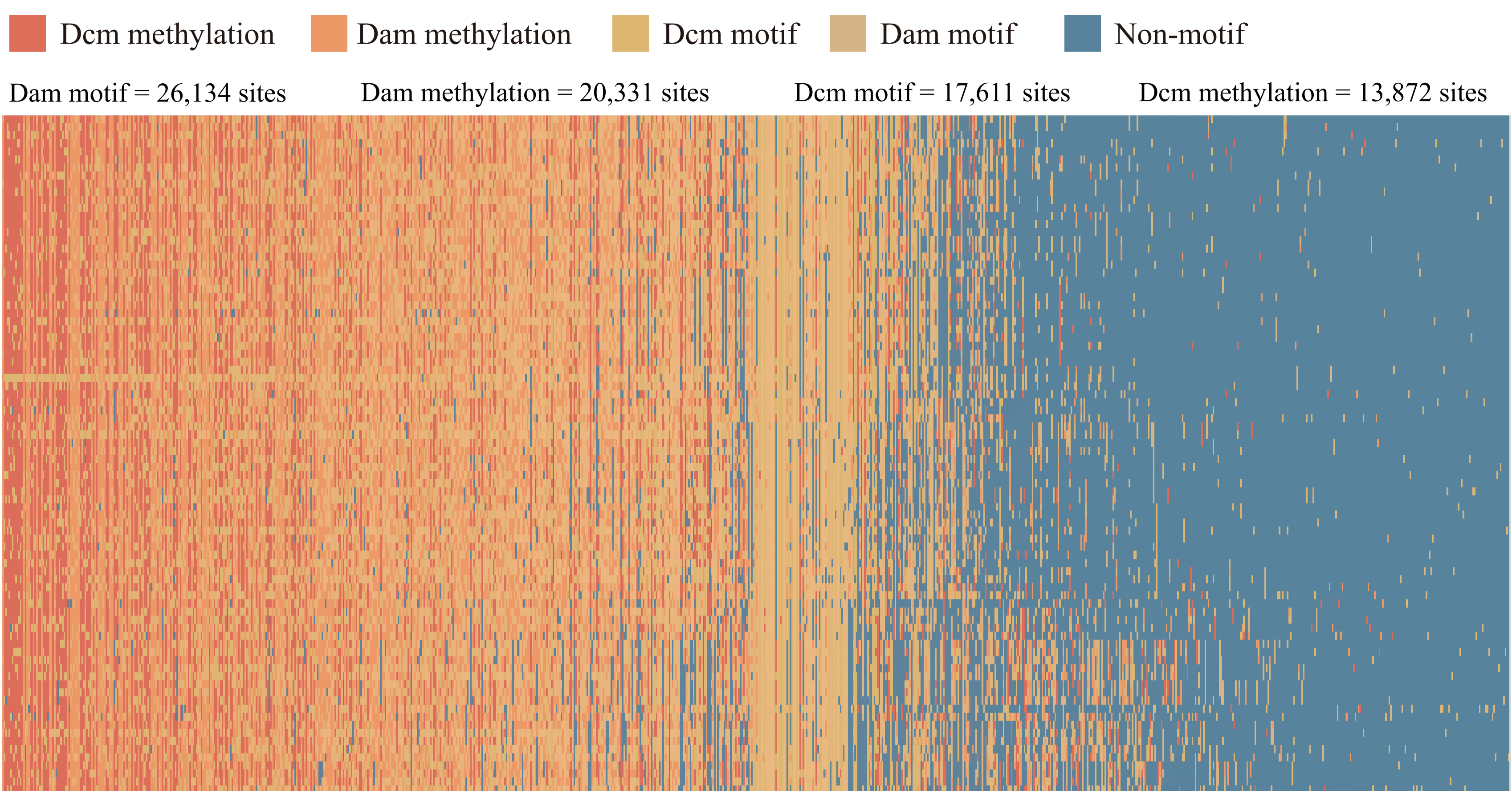

B

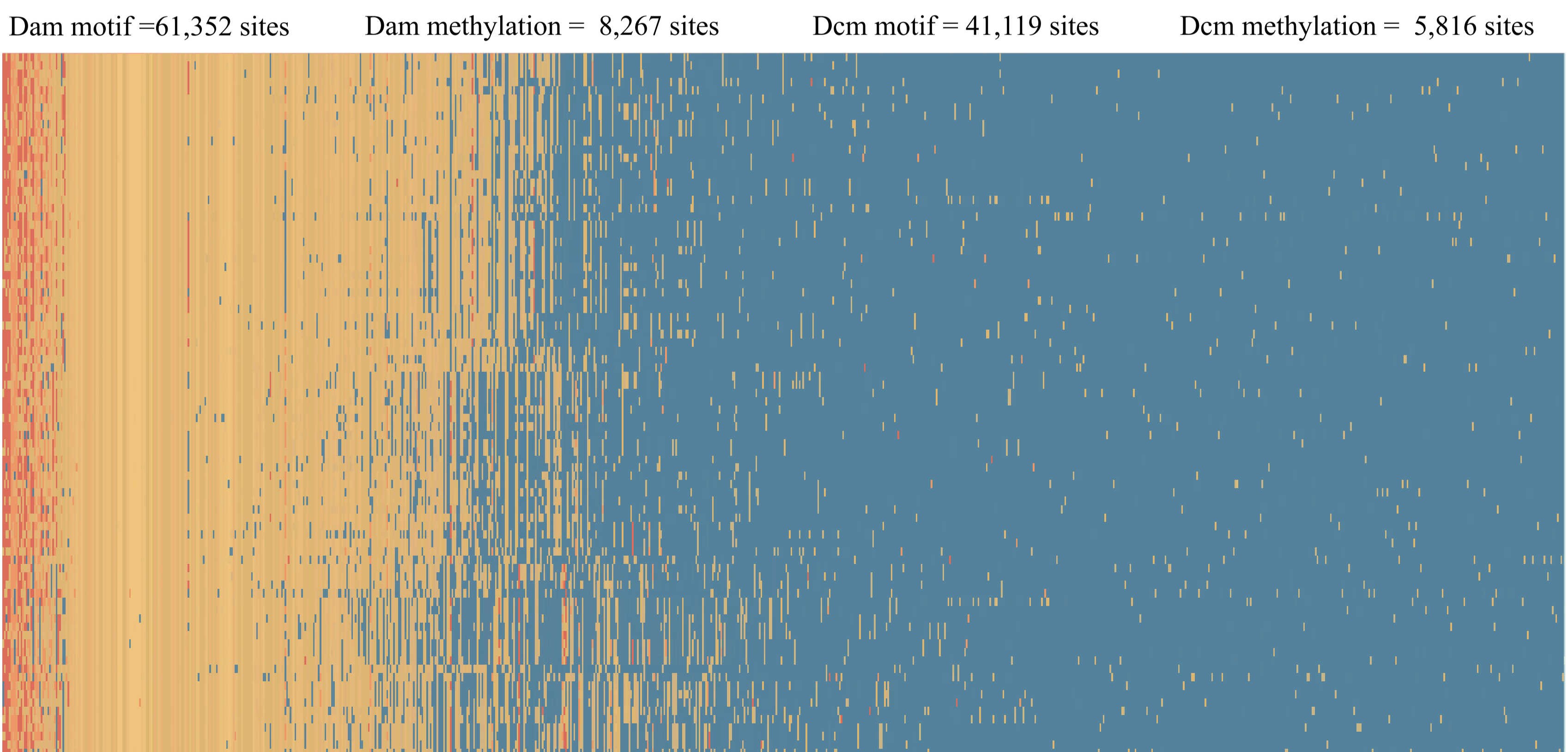

Supplement: Supplementary file 1 — Supporting File 1: advs76559‐sup‐0001‐SuppMatfiguresS1‐S21.zip [file ADVS-9999-e76559-s003.zip › S6.pdf]

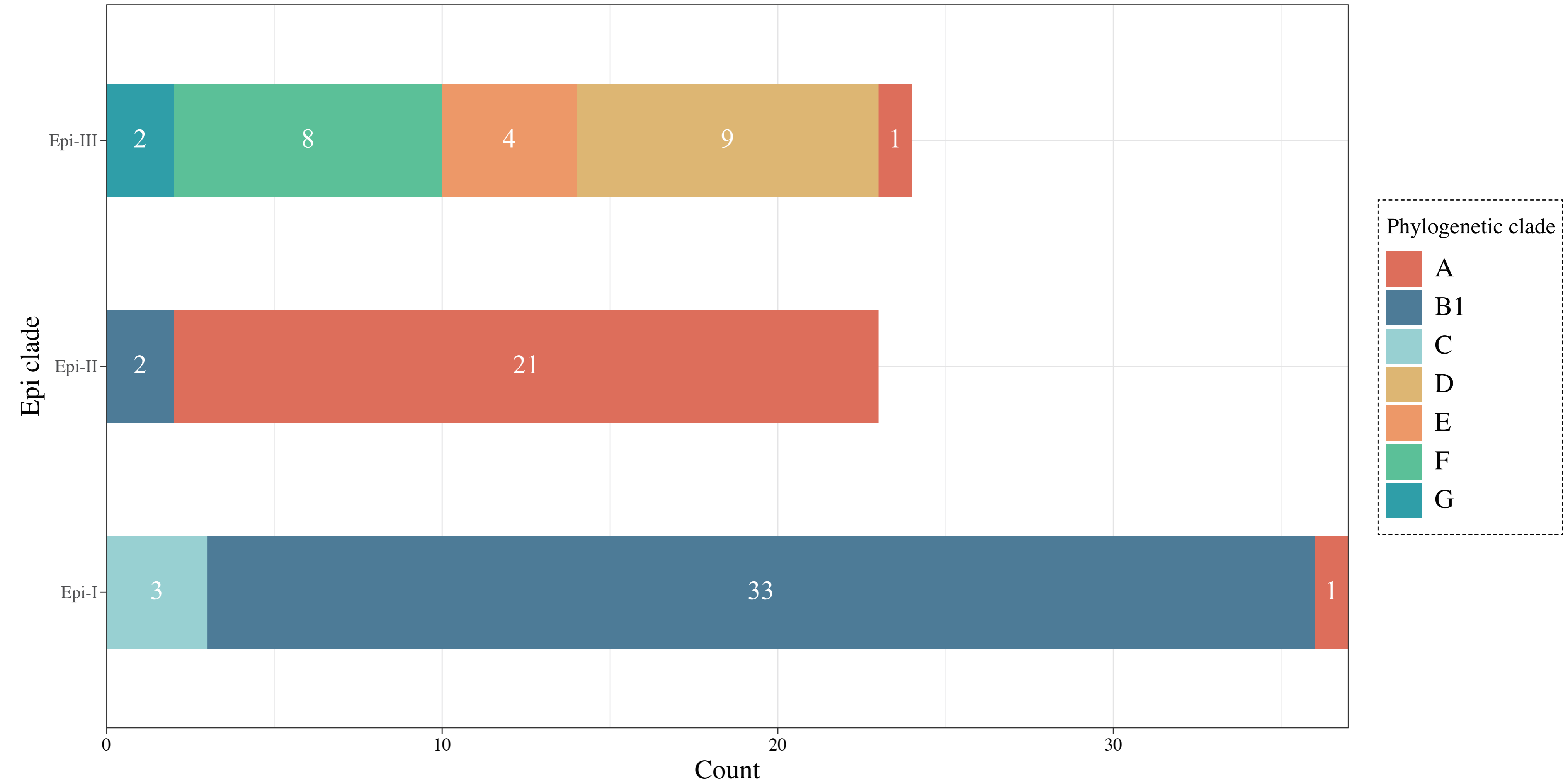

Supplement: Supplementary file 1 — Supporting File 1: advs76559‐sup‐0001‐SuppMatfiguresS1‐S21.zip [file ADVS-9999-e76559-s003.zip › S7.pdf]

### Low temperature (25°C)

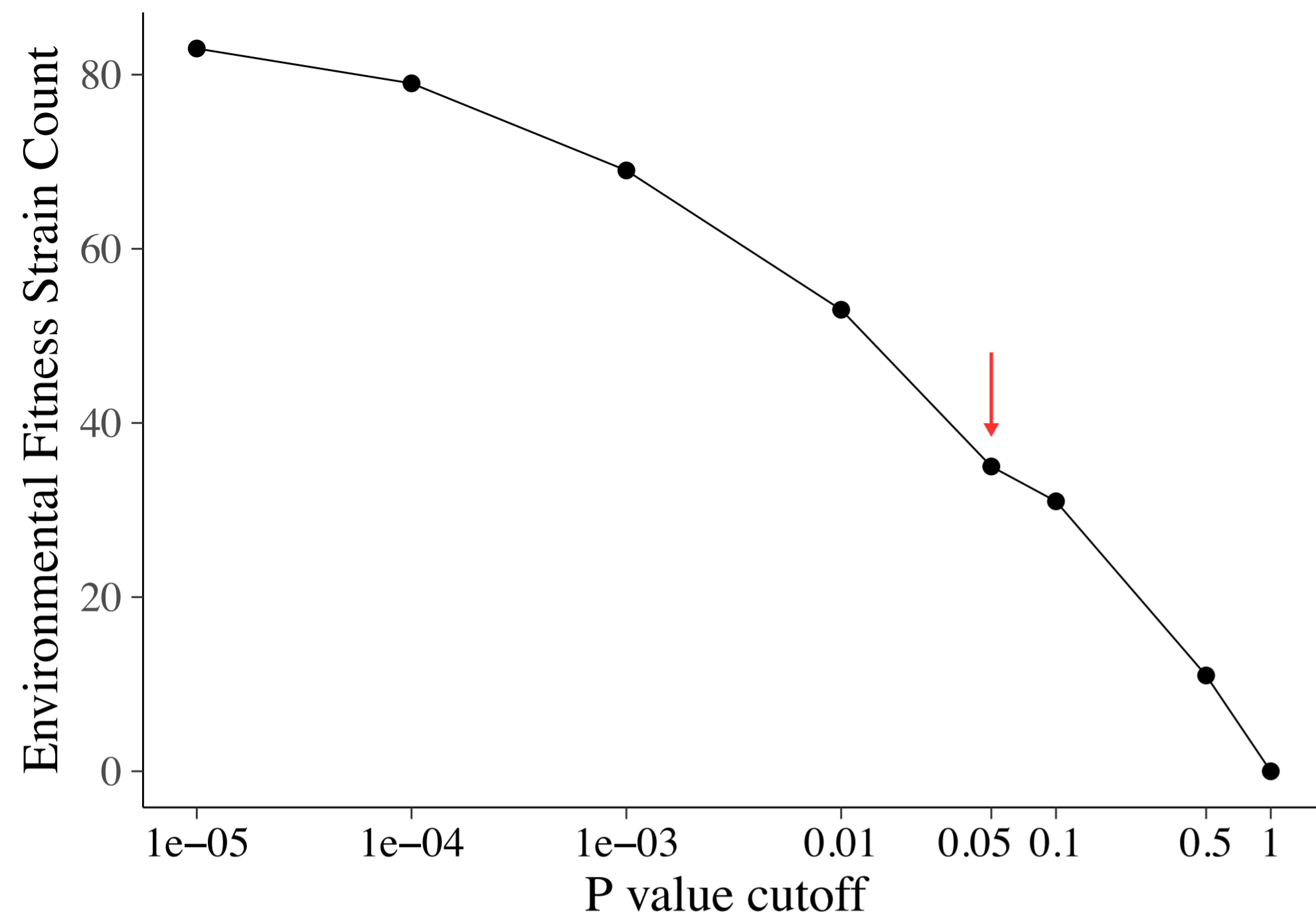

### High temperature (45°C)

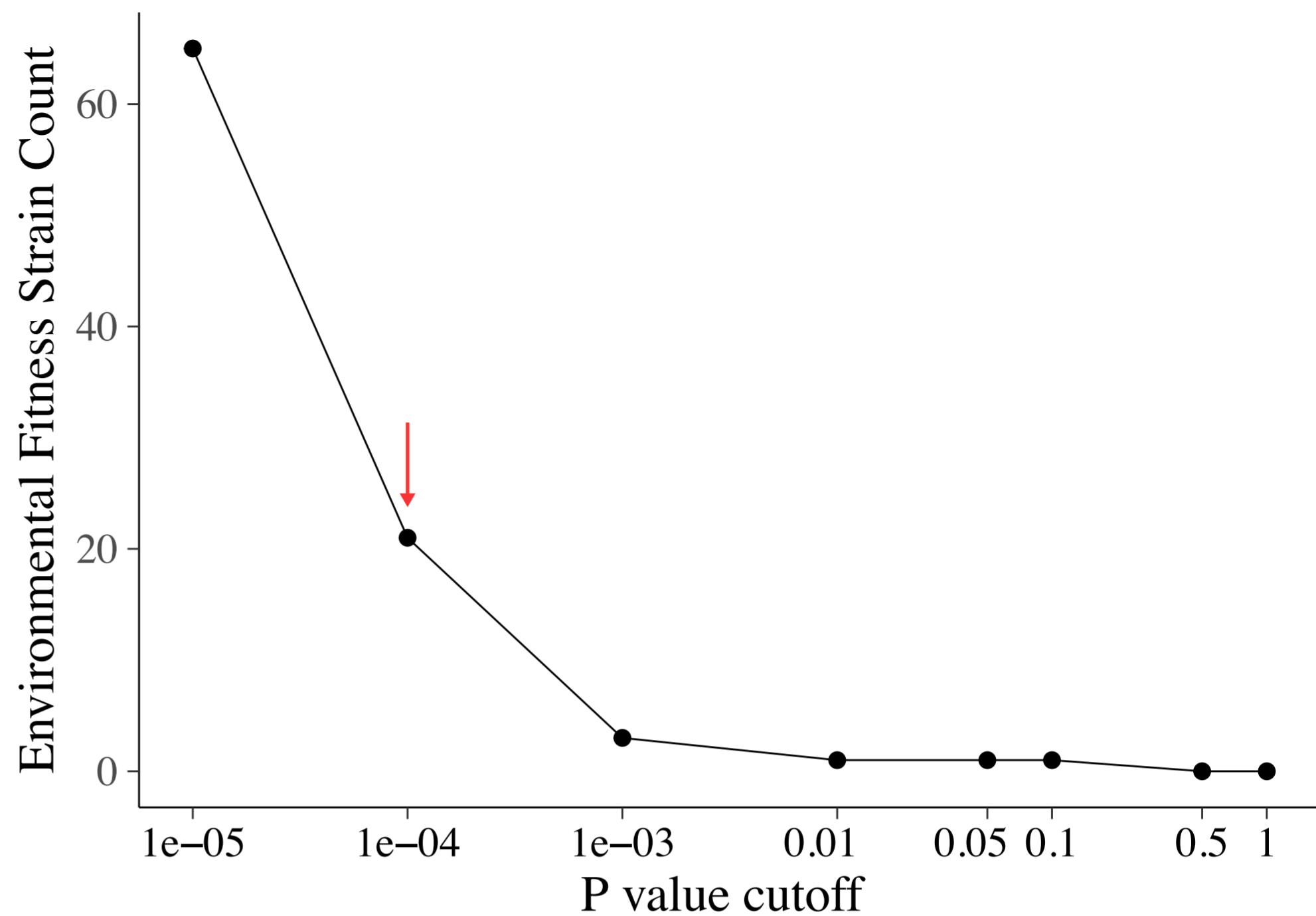

### Acidic (PH=5)

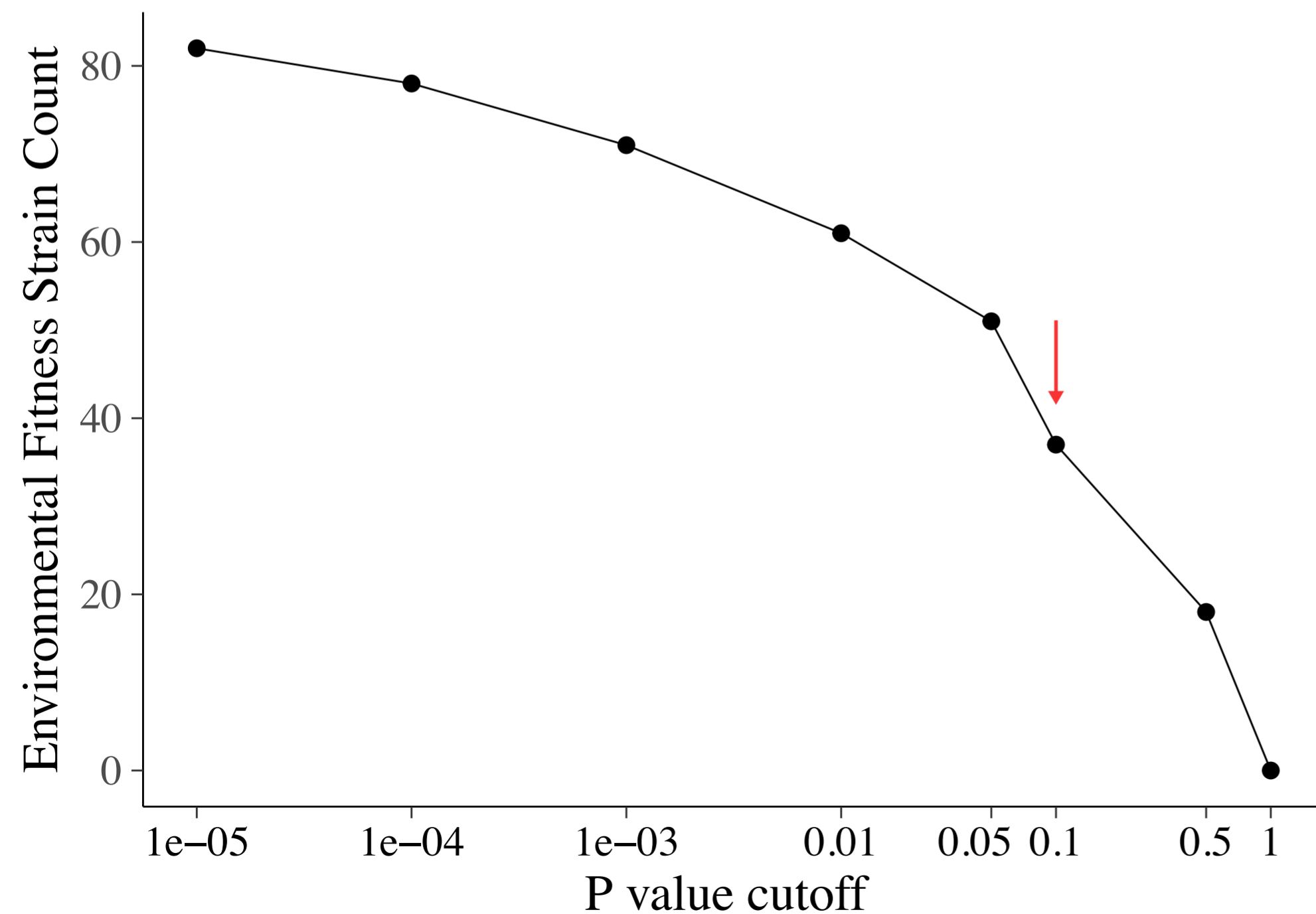

### Alkaline (PH=9)

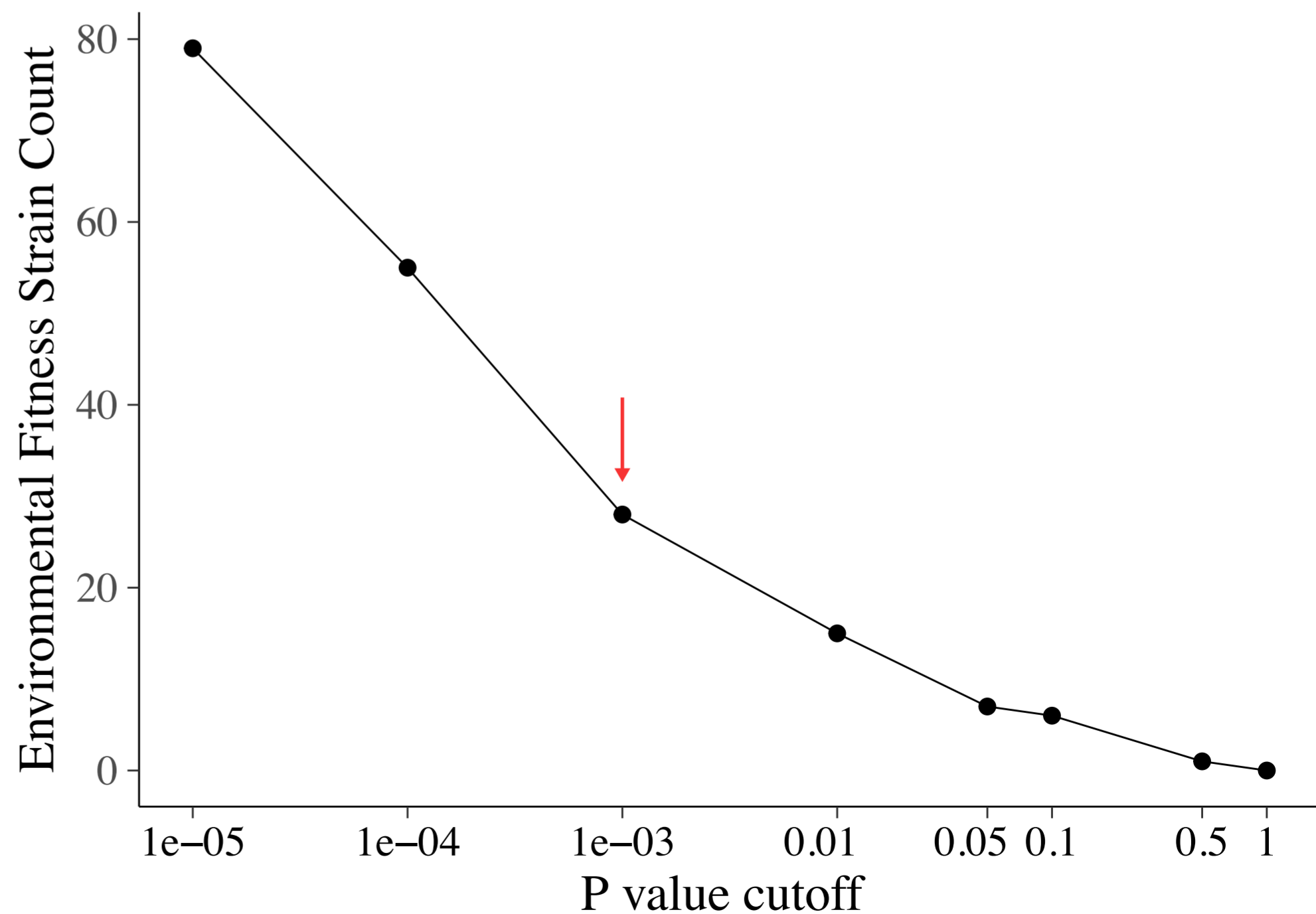

Supplement: Supplementary file 1 — Supporting File 1: advs76559‐sup‐0001‐SuppMatfiguresS1‐S21.zip [file ADVS-9999-e76559-s003.zip › S8.pdf]

A

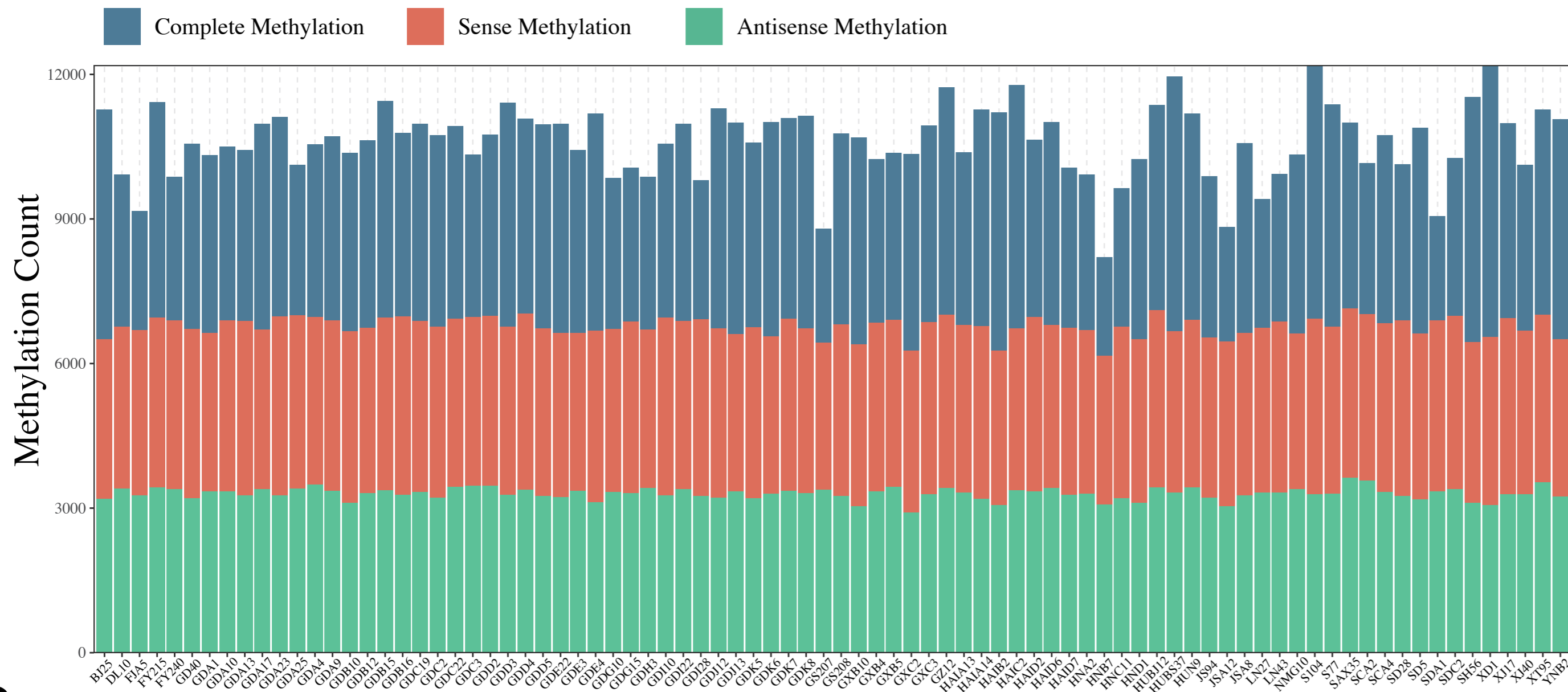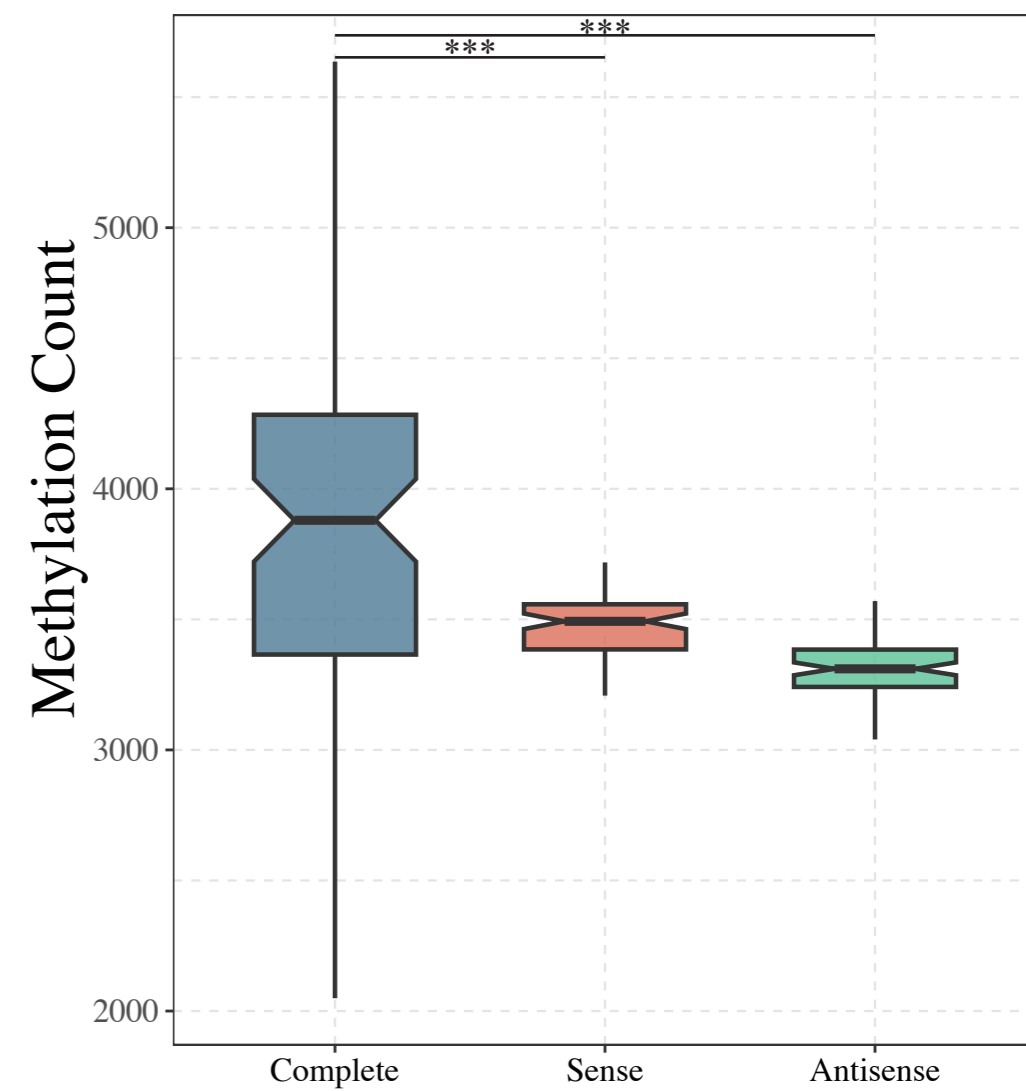

B

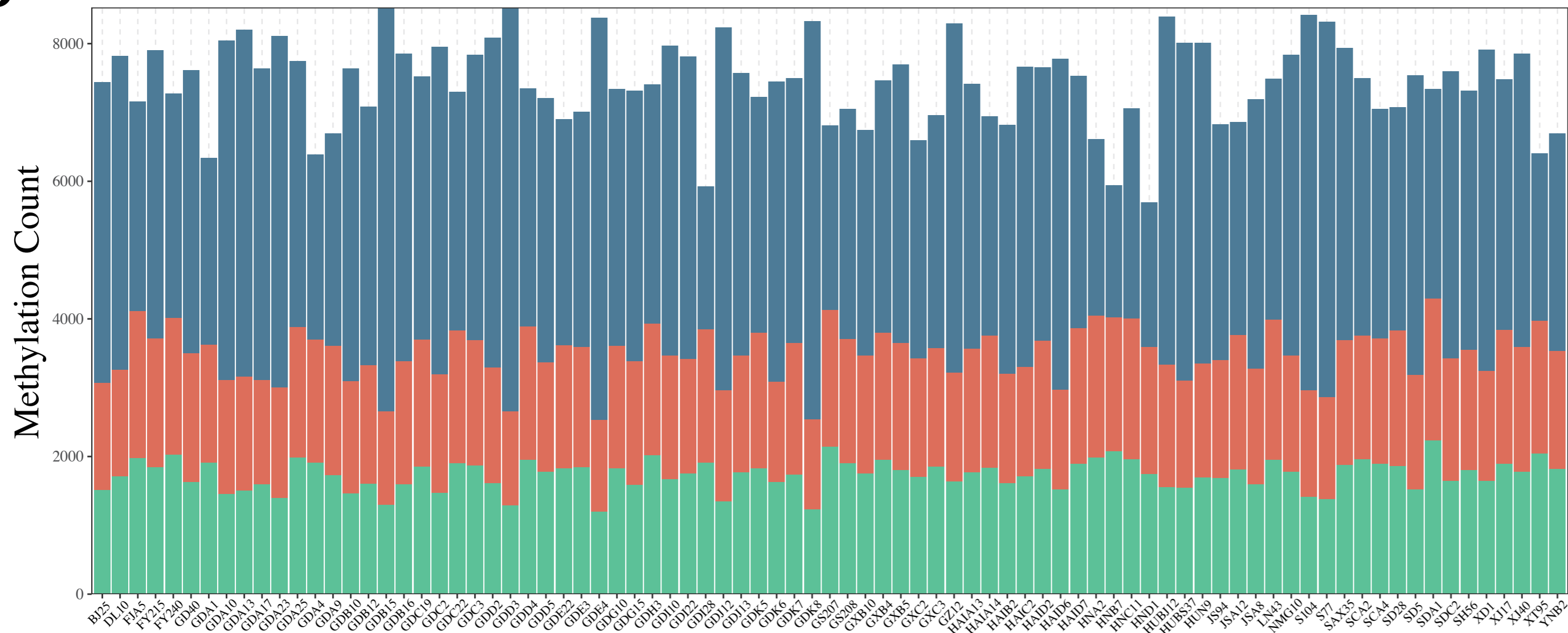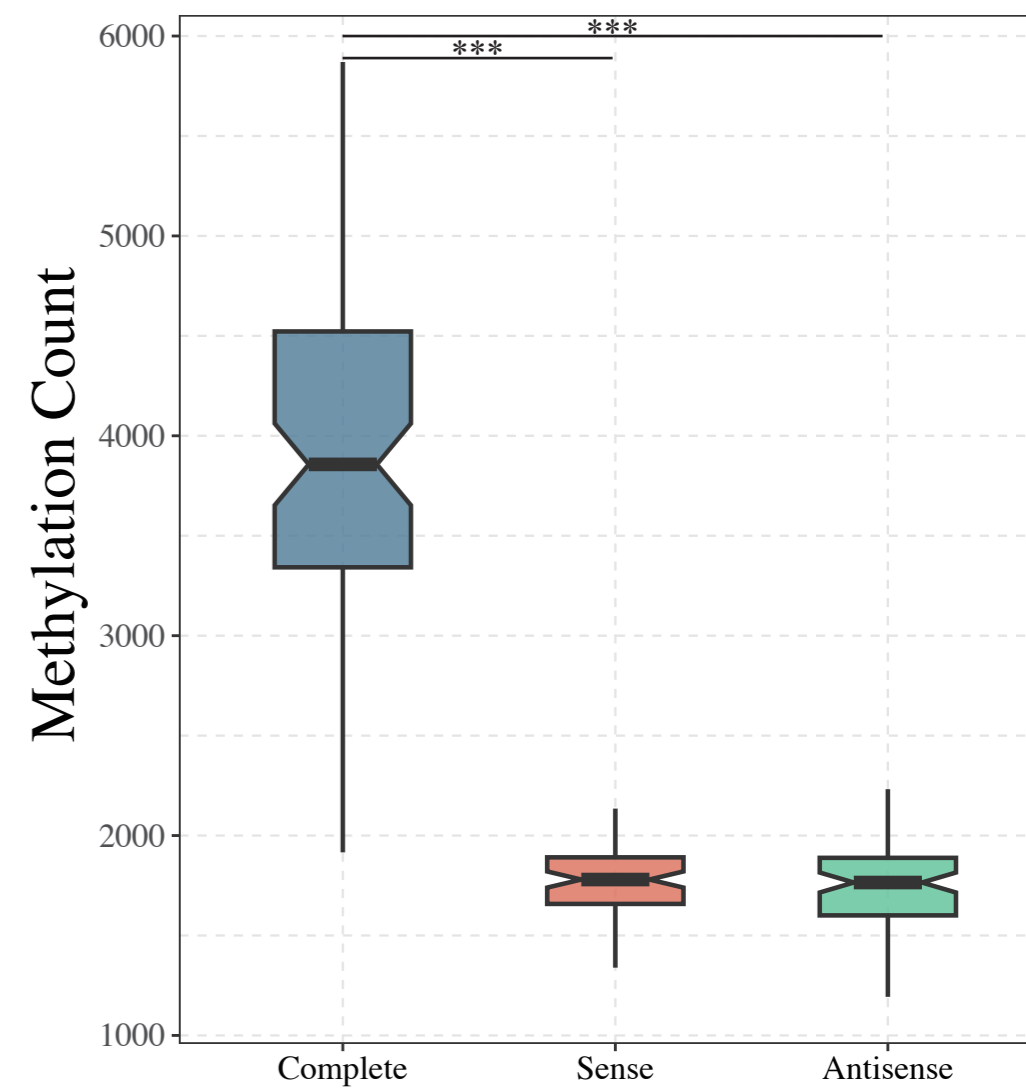

Supplement: Supplementary file 1 — Supporting File 1: advs76559‐sup‐0001‐SuppMatfiguresS1‐S21.zip [file ADVS-9999-e76559-s003.zip › S9.pdf]

## Antisense Methylation

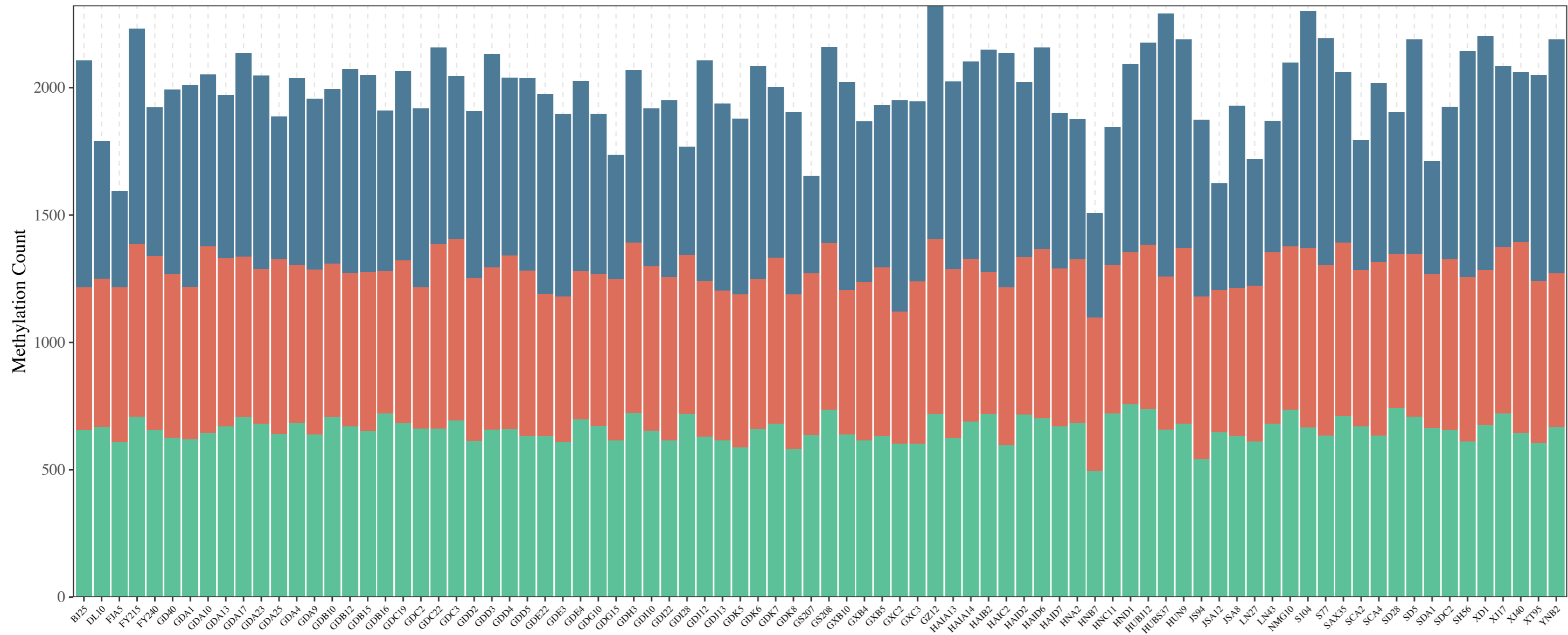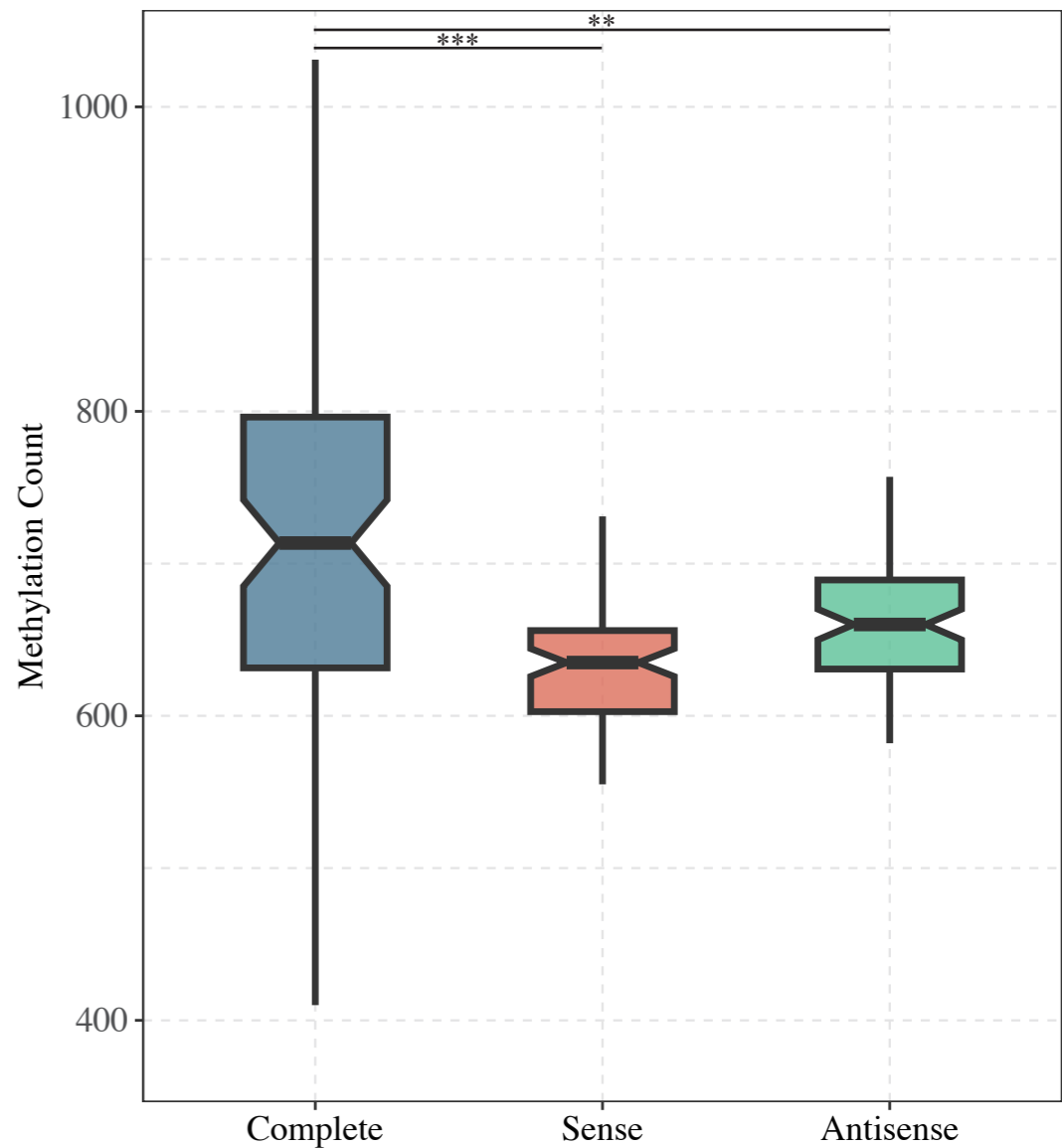

B

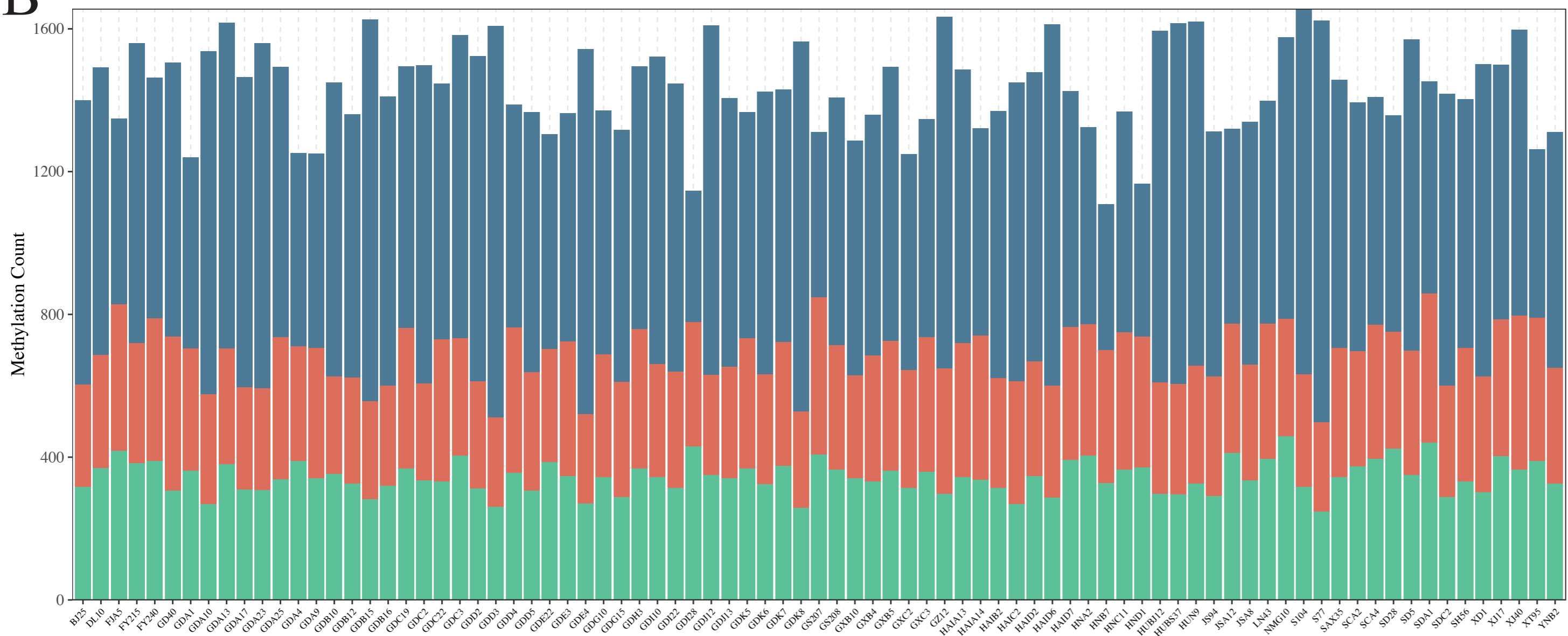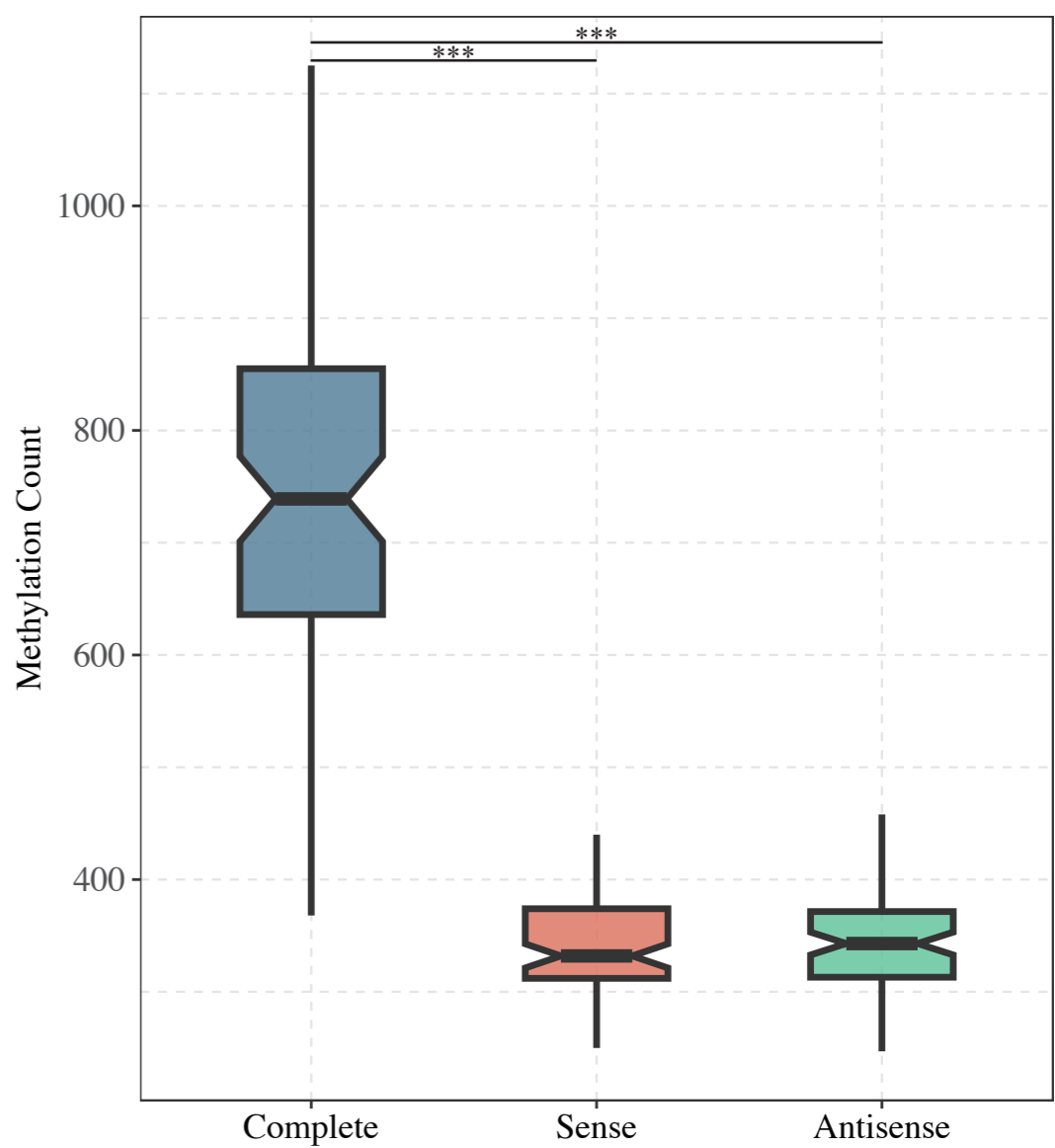

Supplement: Supplementary file 1 — Supporting File 1: advs76559‐sup‐0001‐SuppMatfiguresS1‐S21.zip [file ADVS-9999-e76559-s003.zip › S10.pdf]

A

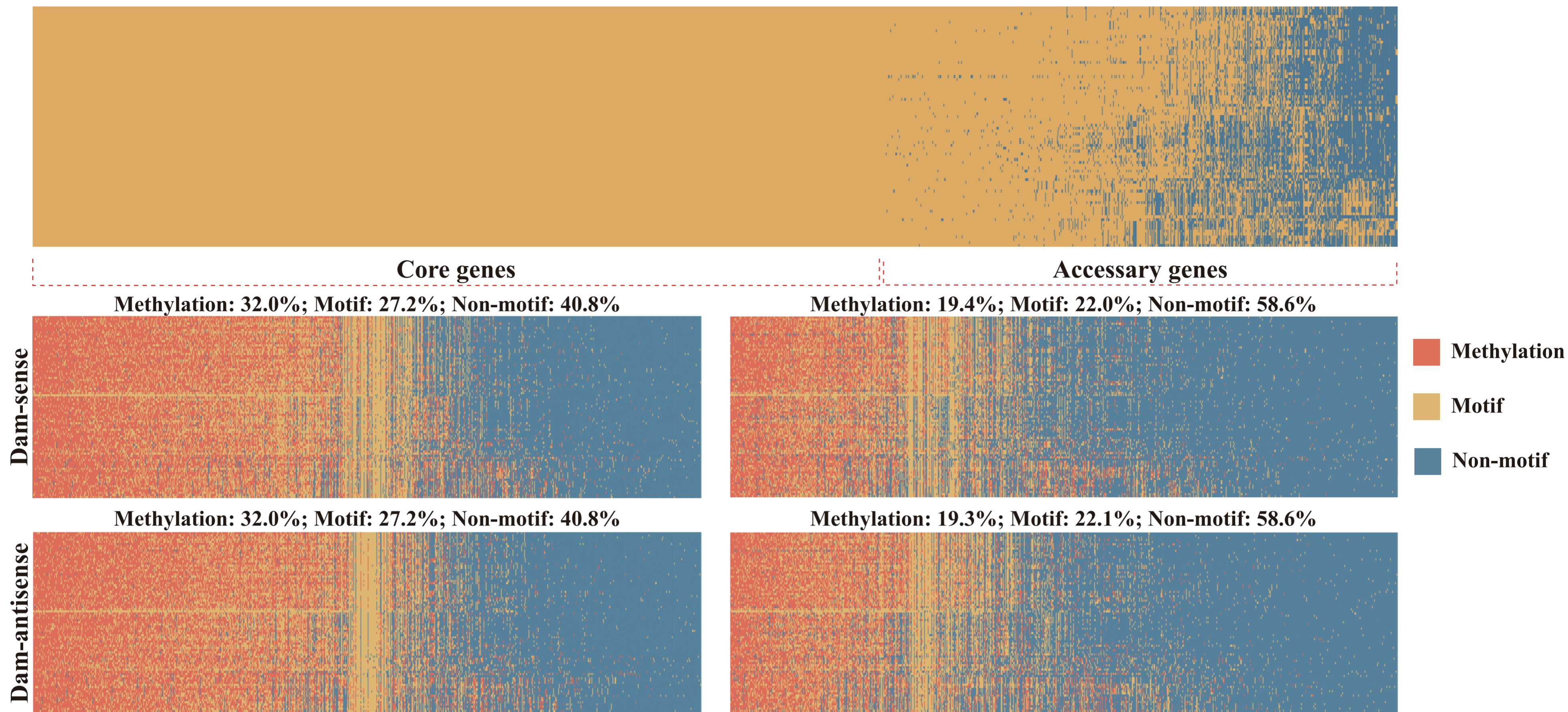

B

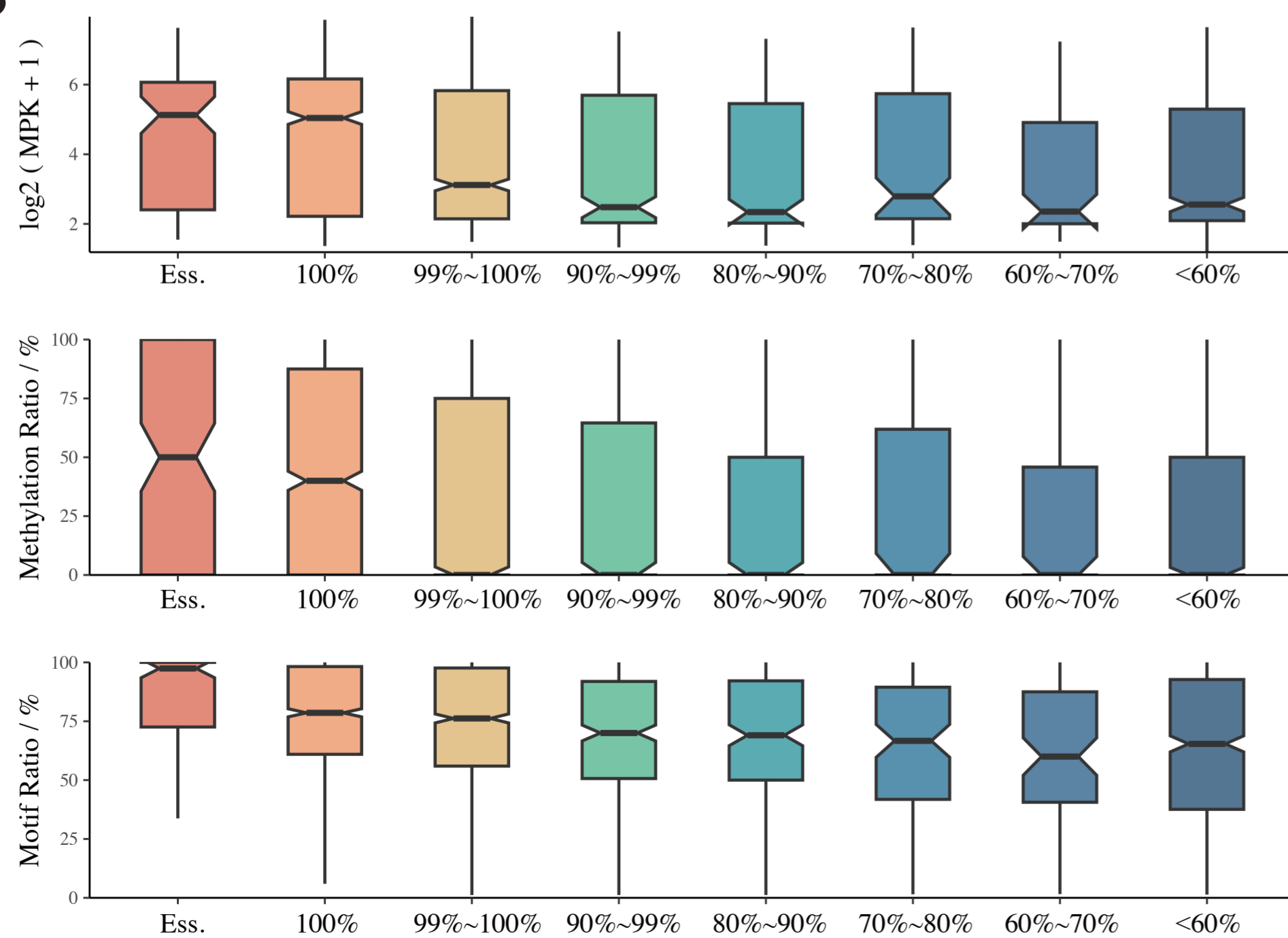

C

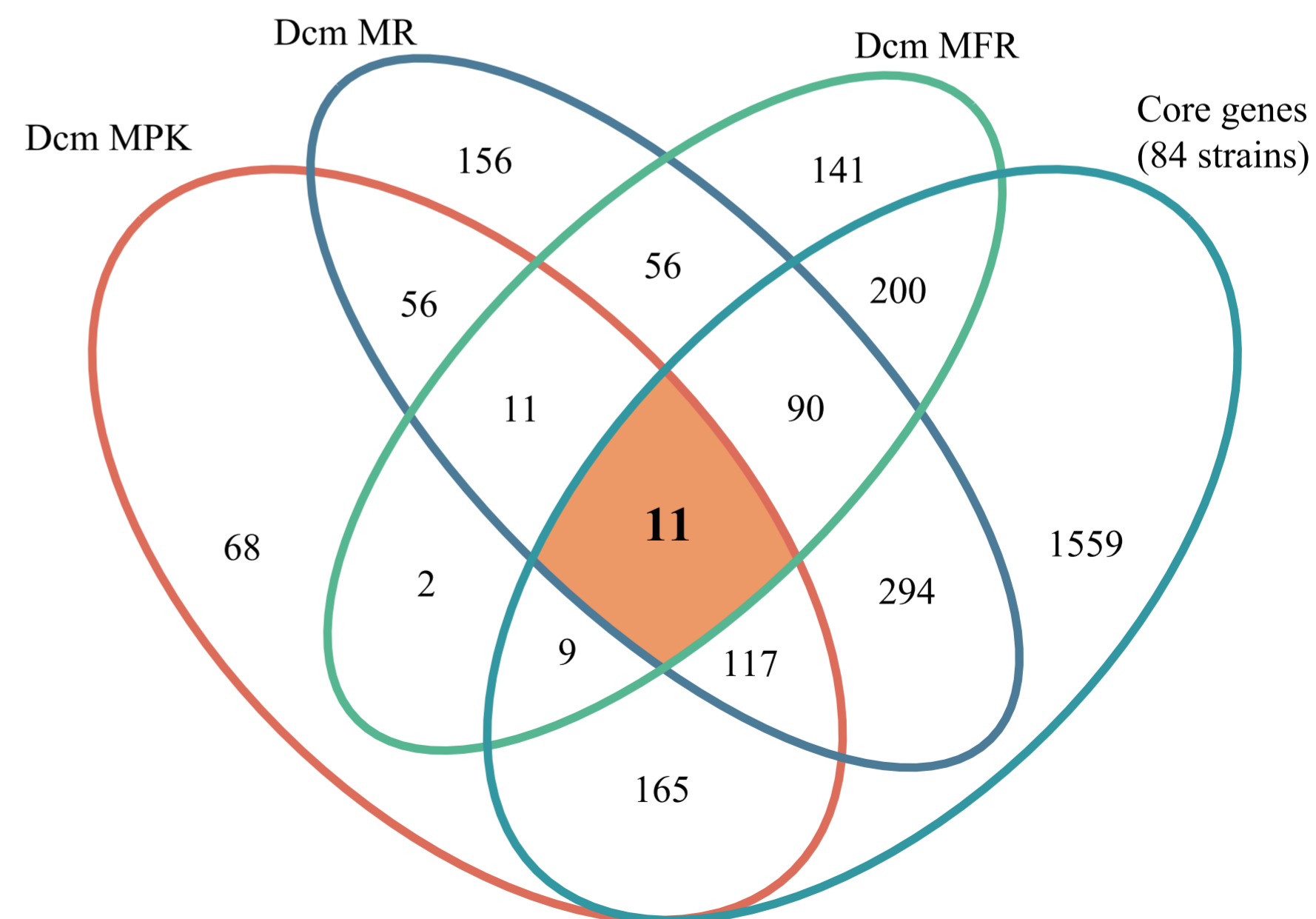

Supplement: Supplementary file 1 — Supporting File 1: advs76559‐sup‐0001‐SuppMatfiguresS1‐S21.zip [file ADVS-9999-e76559-s003.zip › S11.pdf]

A

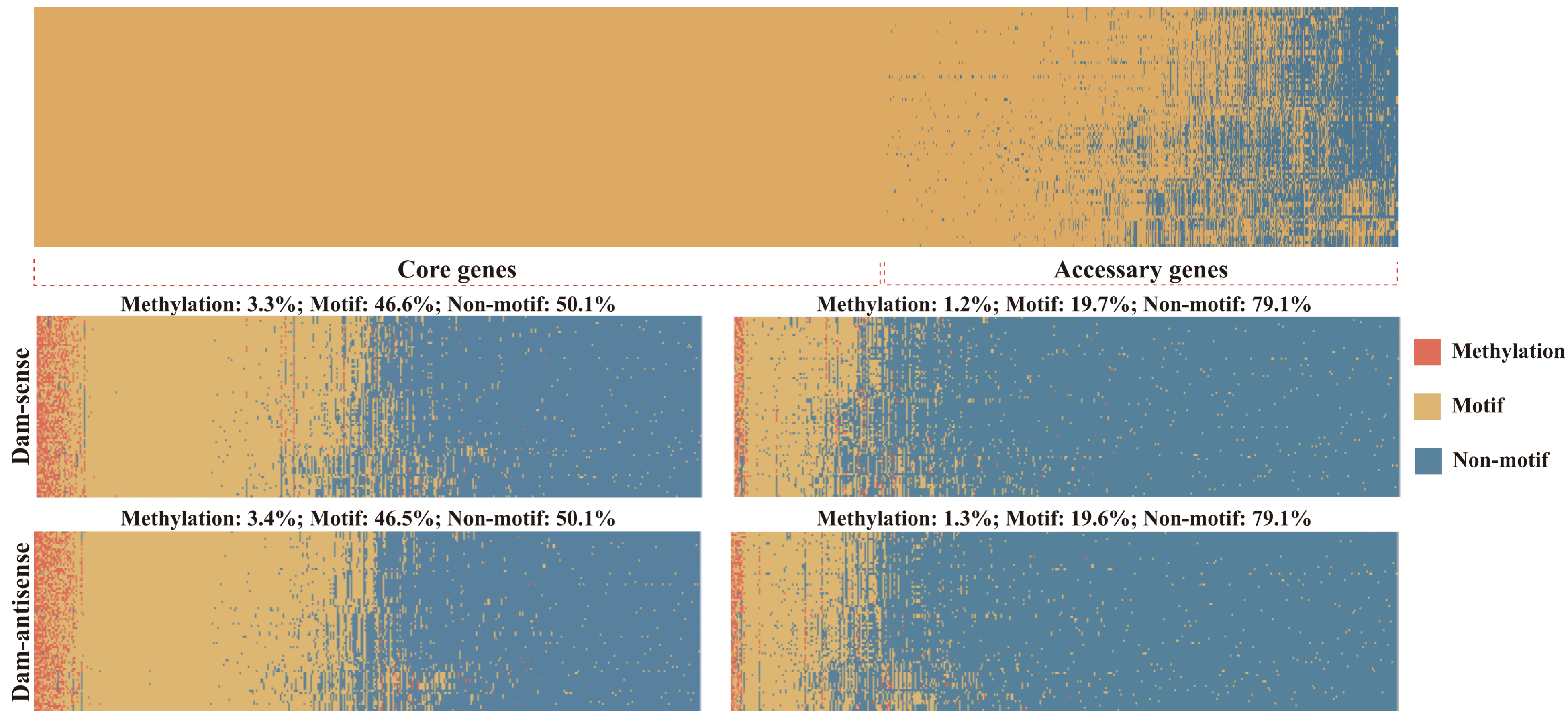

B

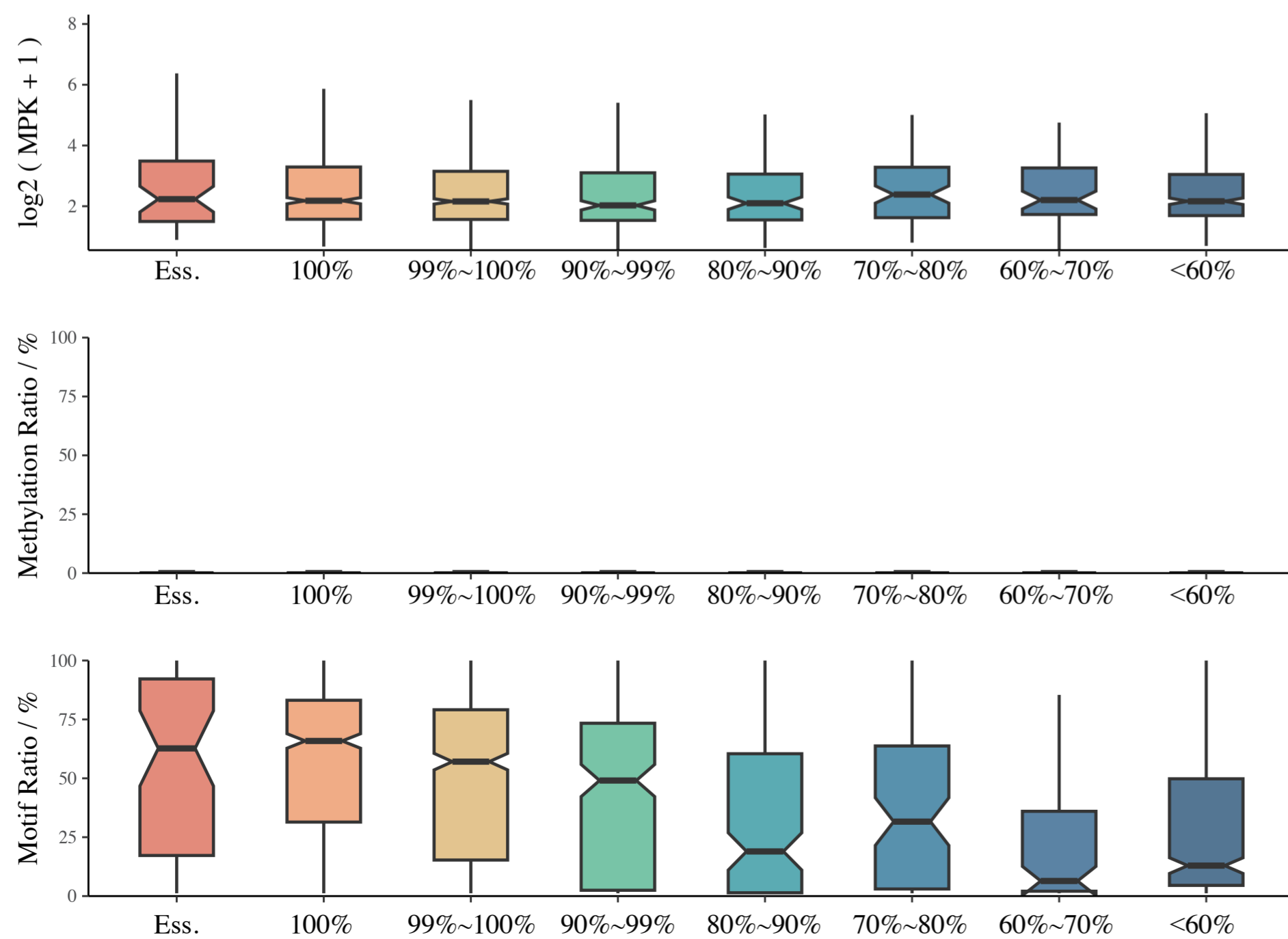

C

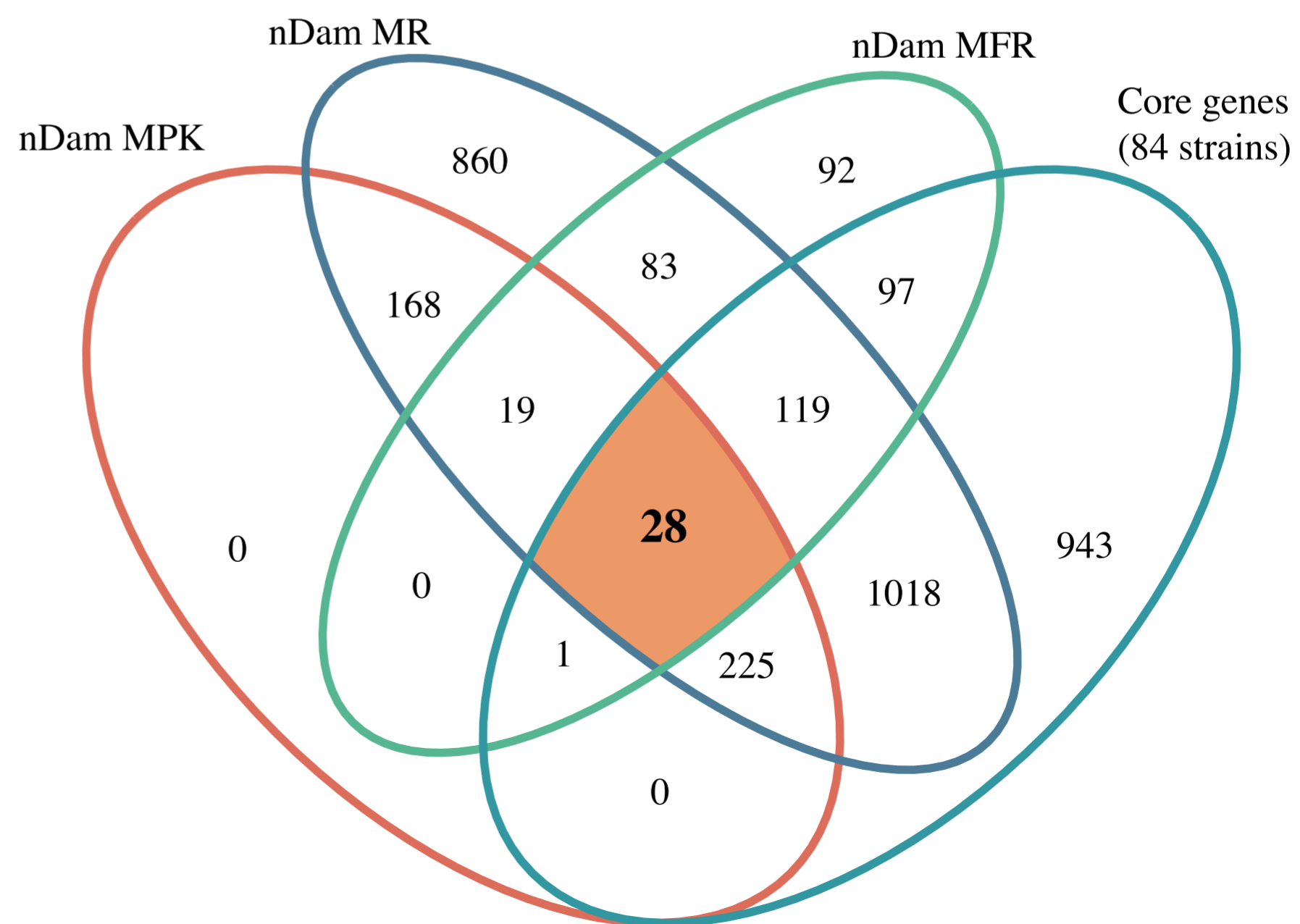

Supplement: Supplementary file 1 — Supporting File 1: advs76559‐sup‐0001‐SuppMatfiguresS1‐S21.zip [file ADVS-9999-e76559-s003.zip › S12.pdf]

A

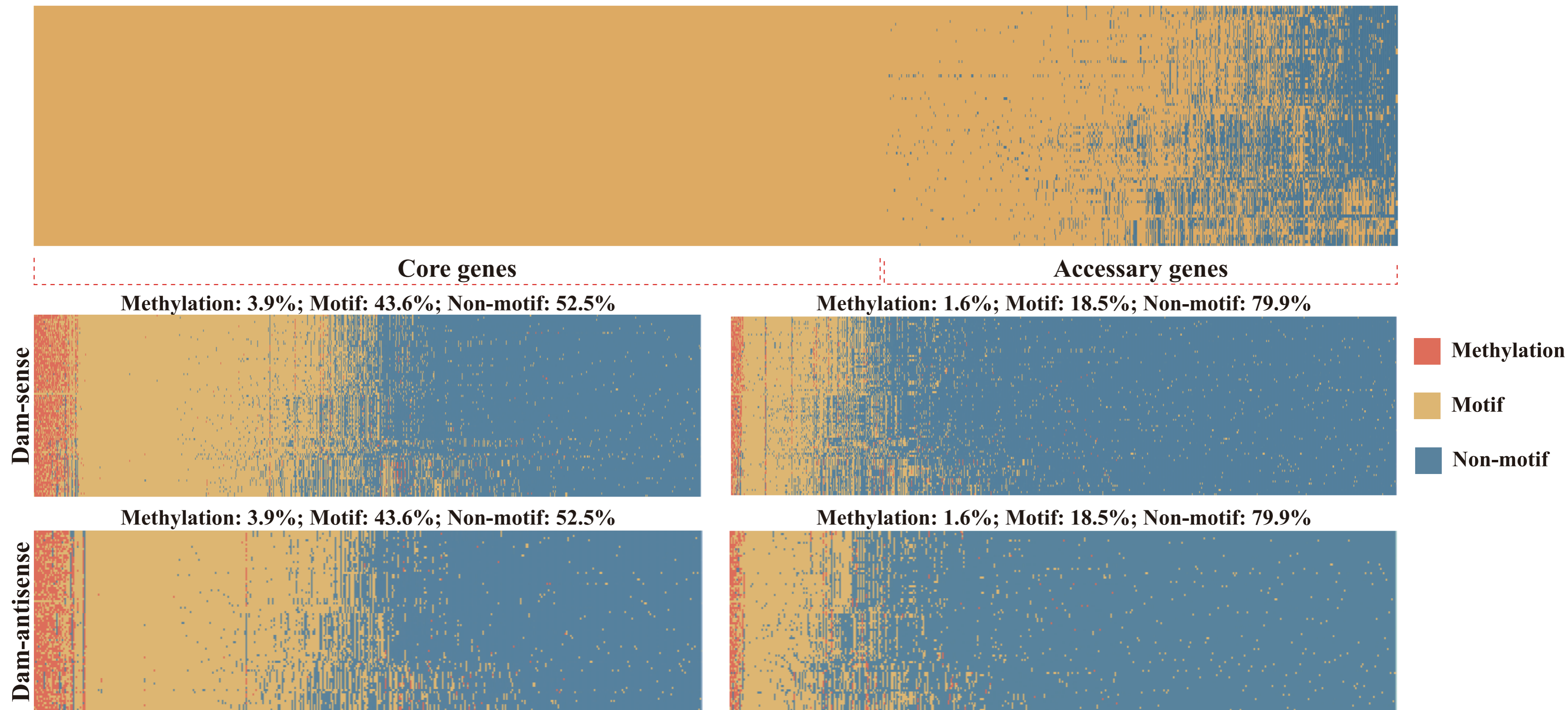

B

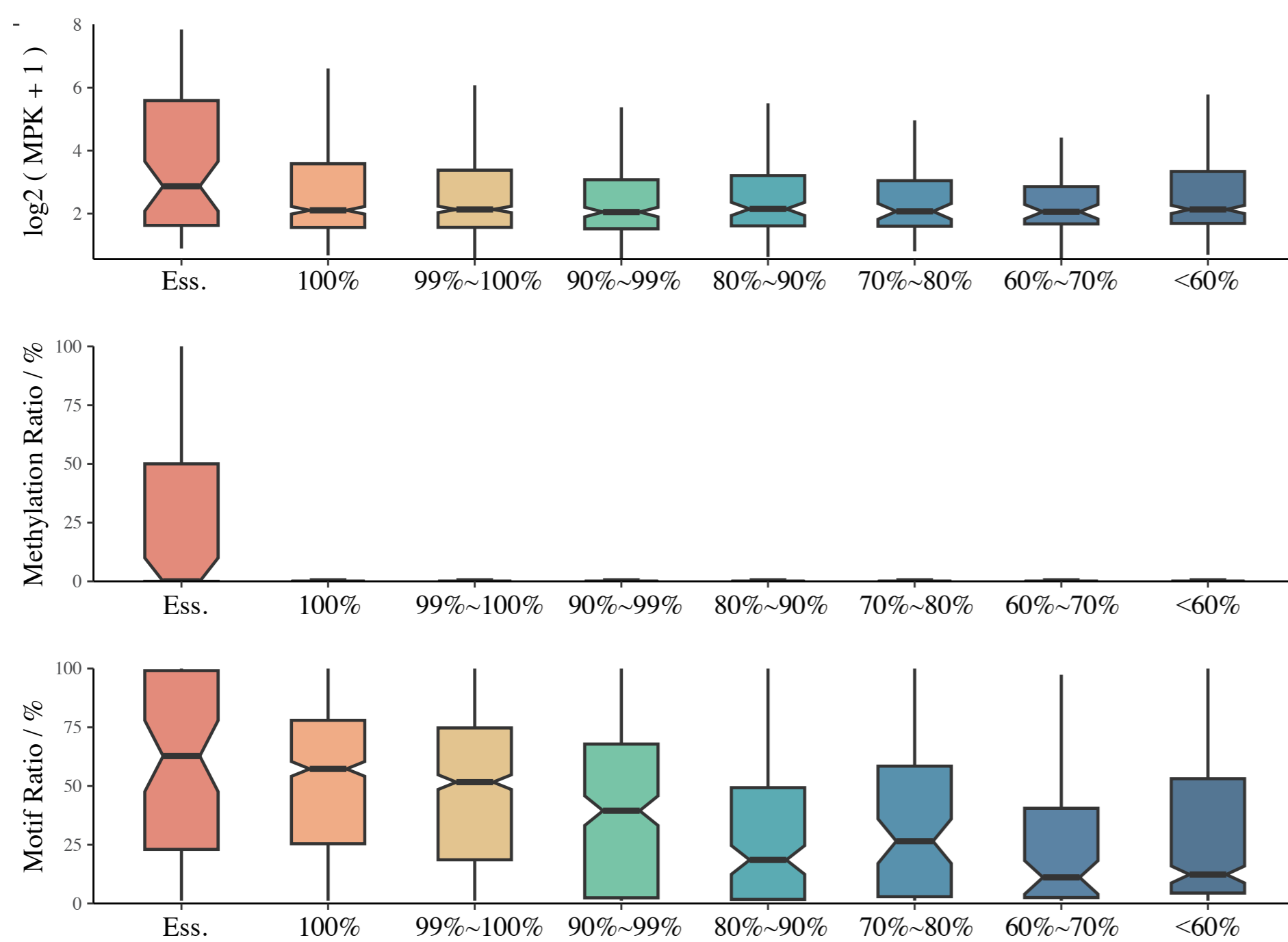

C

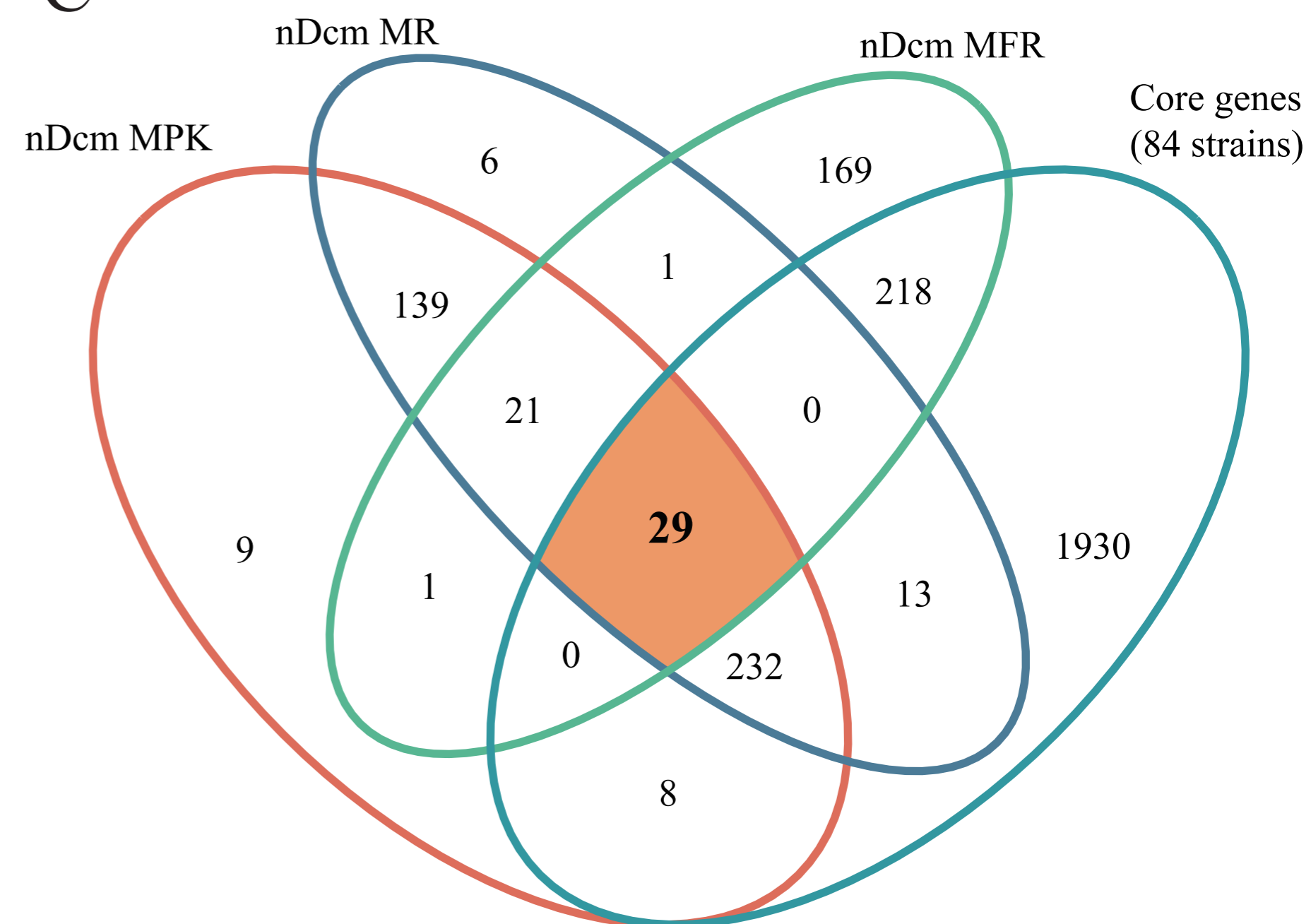

Supplement: Supplementary file 1 — Supporting File 1: advs76559‐sup‐0001‐SuppMatfiguresS1‐S21.zip [file ADVS-9999-e76559-s003.zip › S13.pdf]

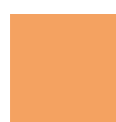 Significant 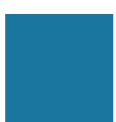 Non-significant

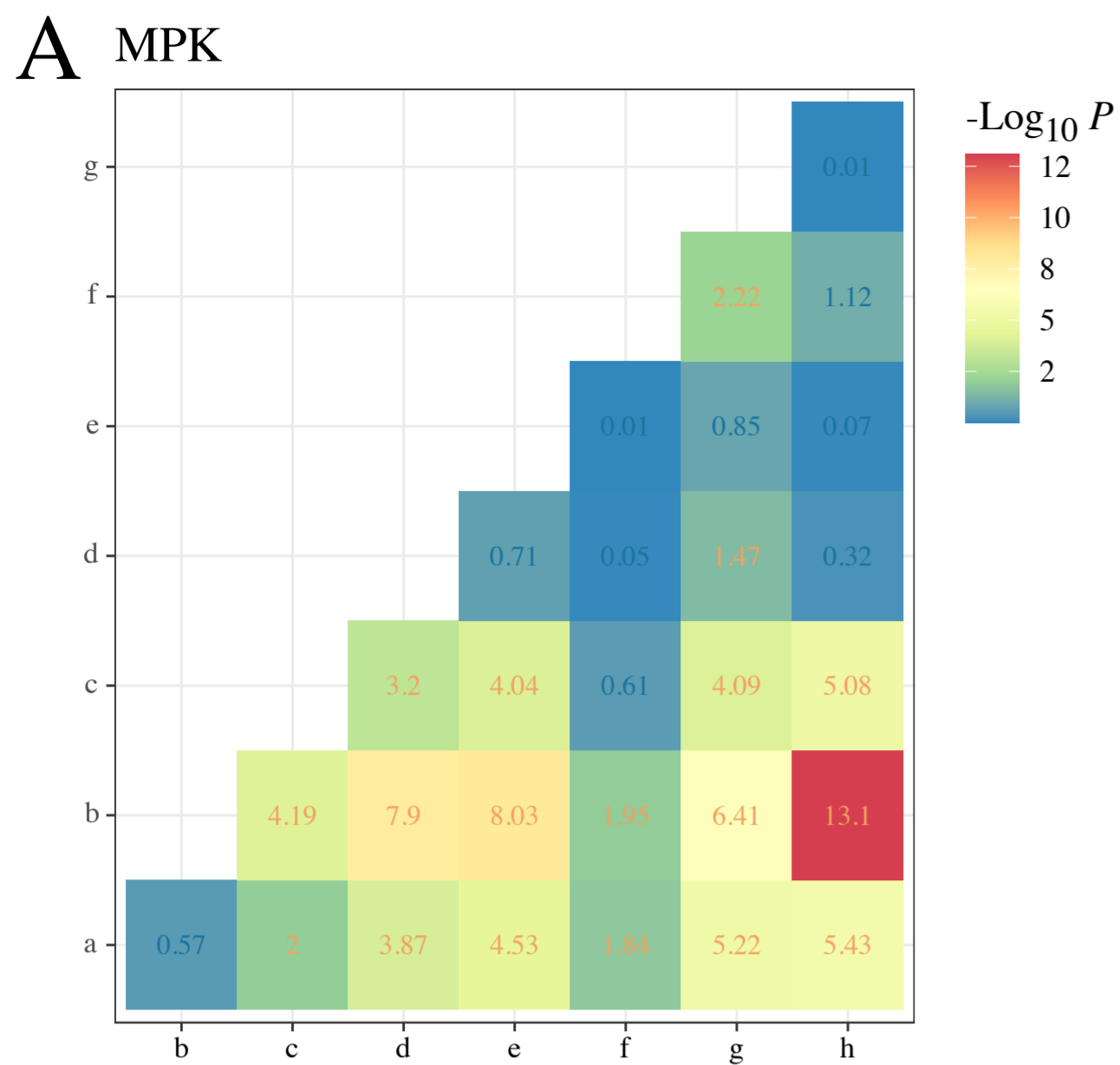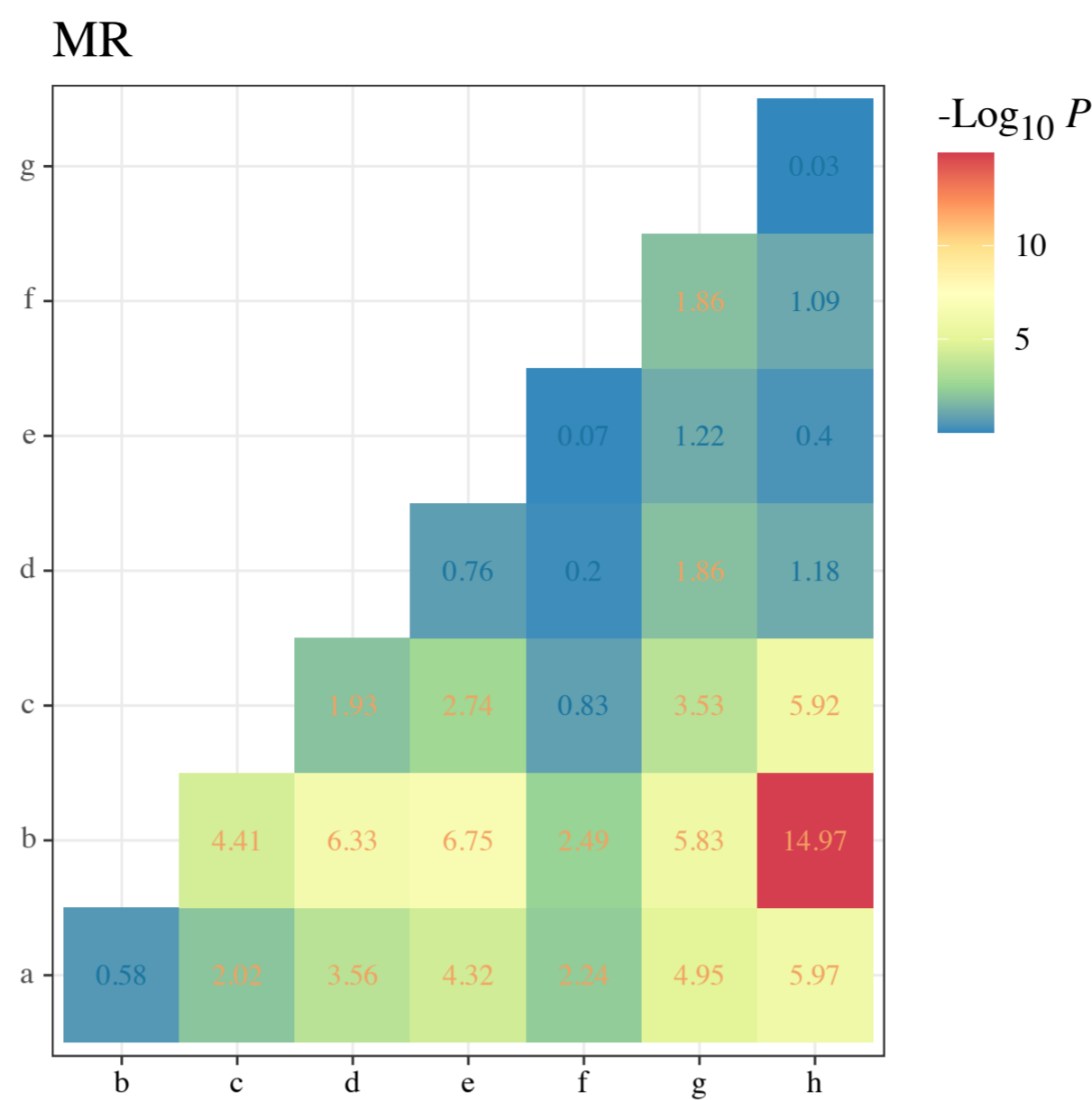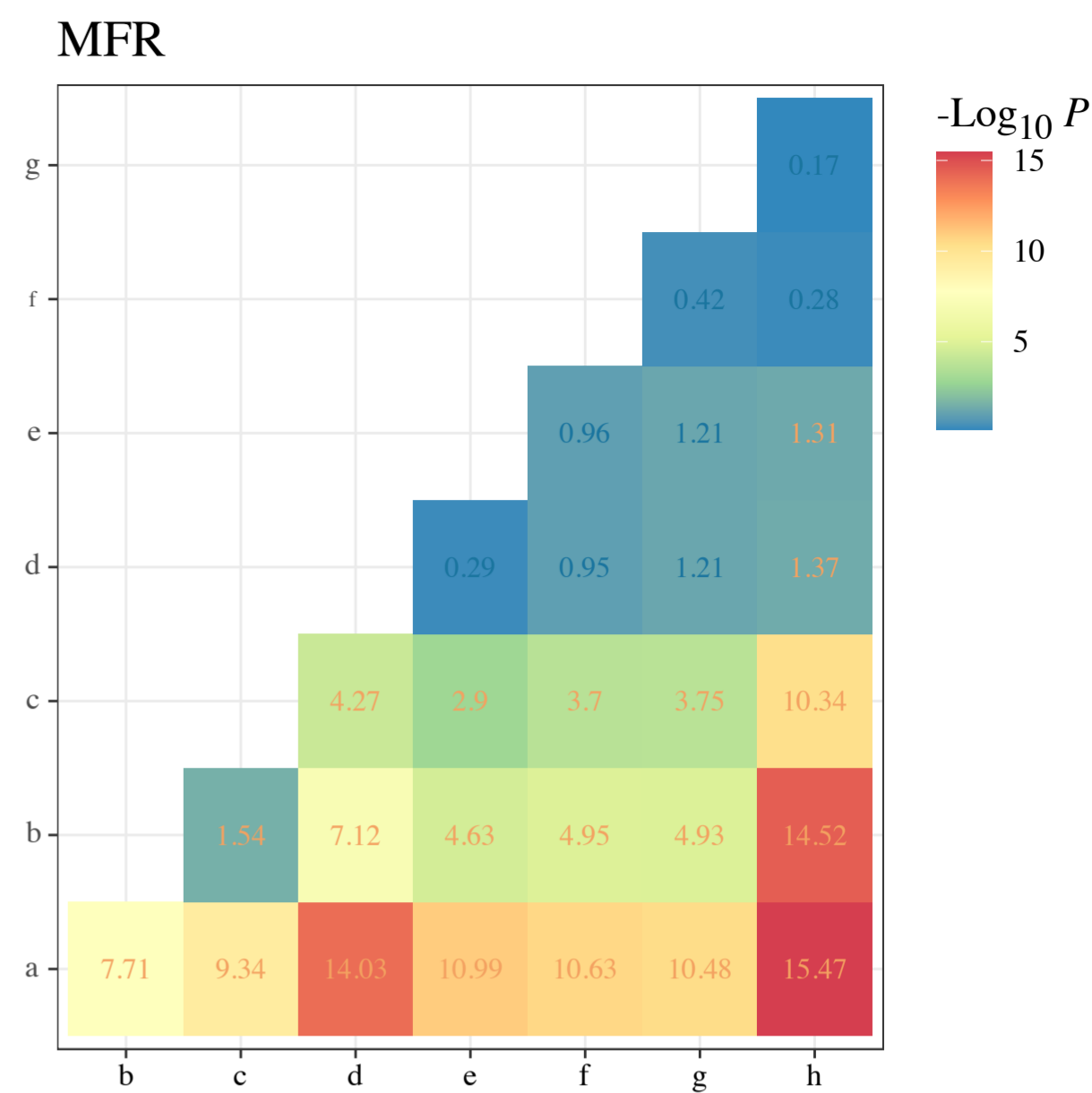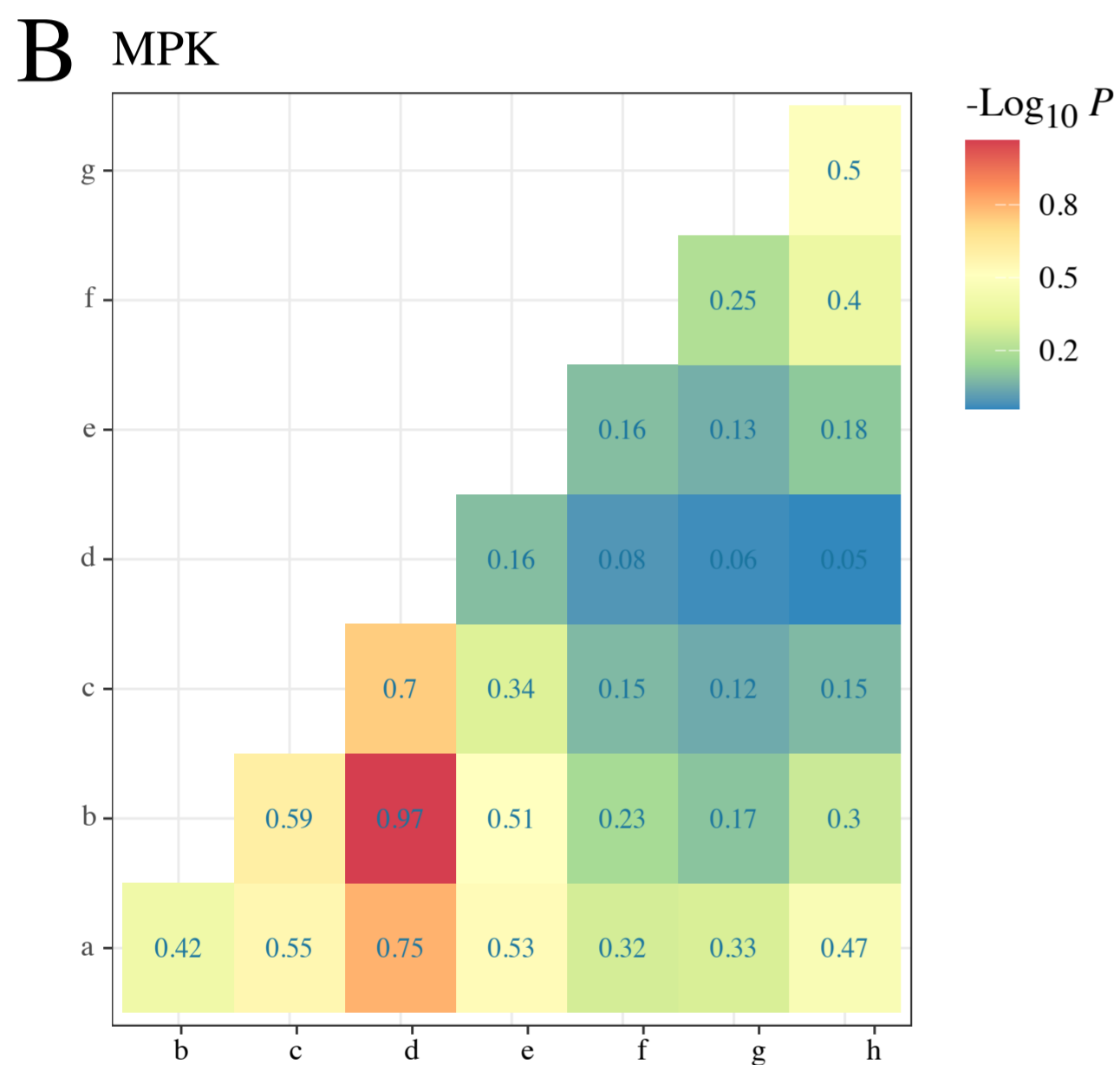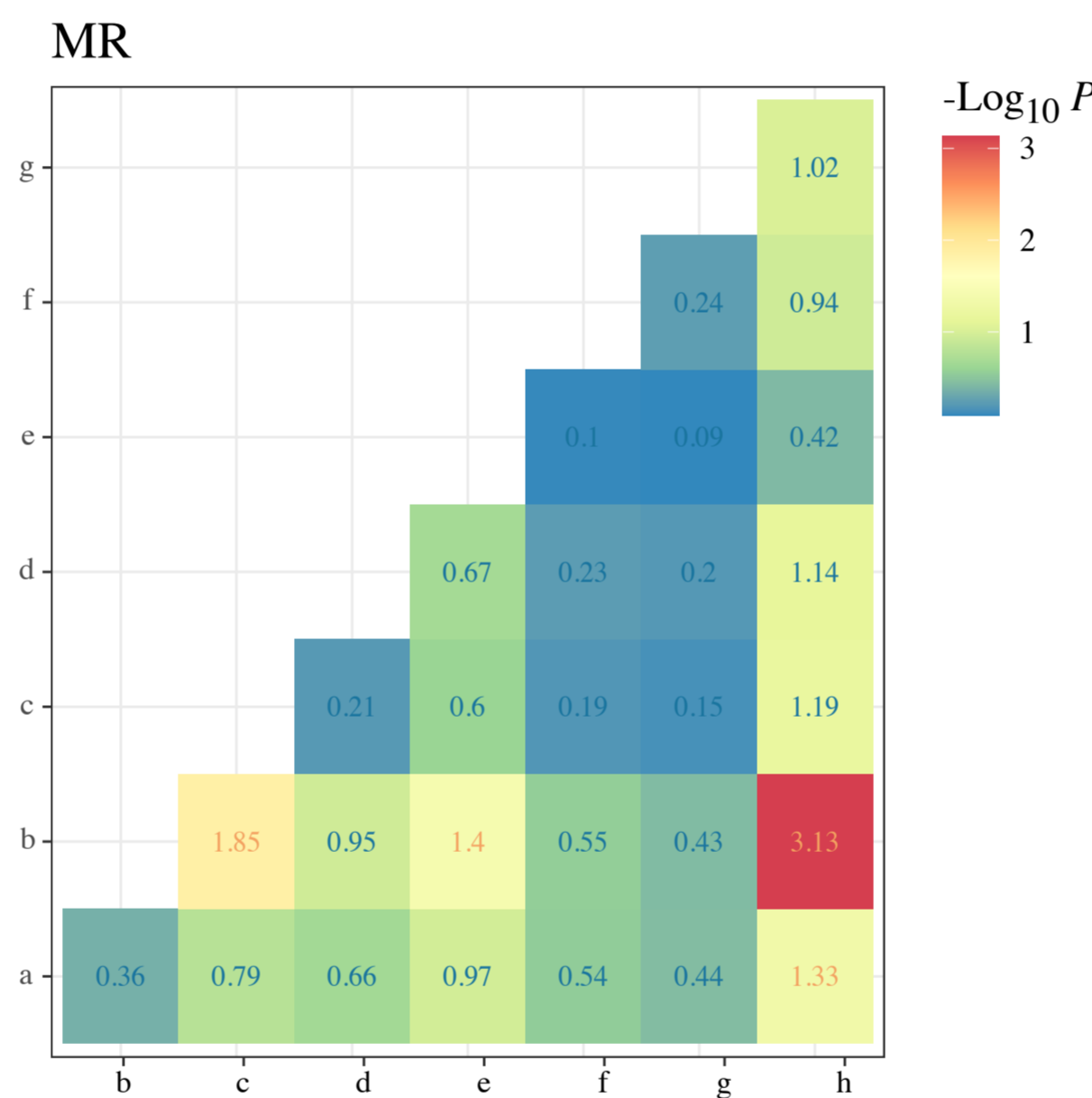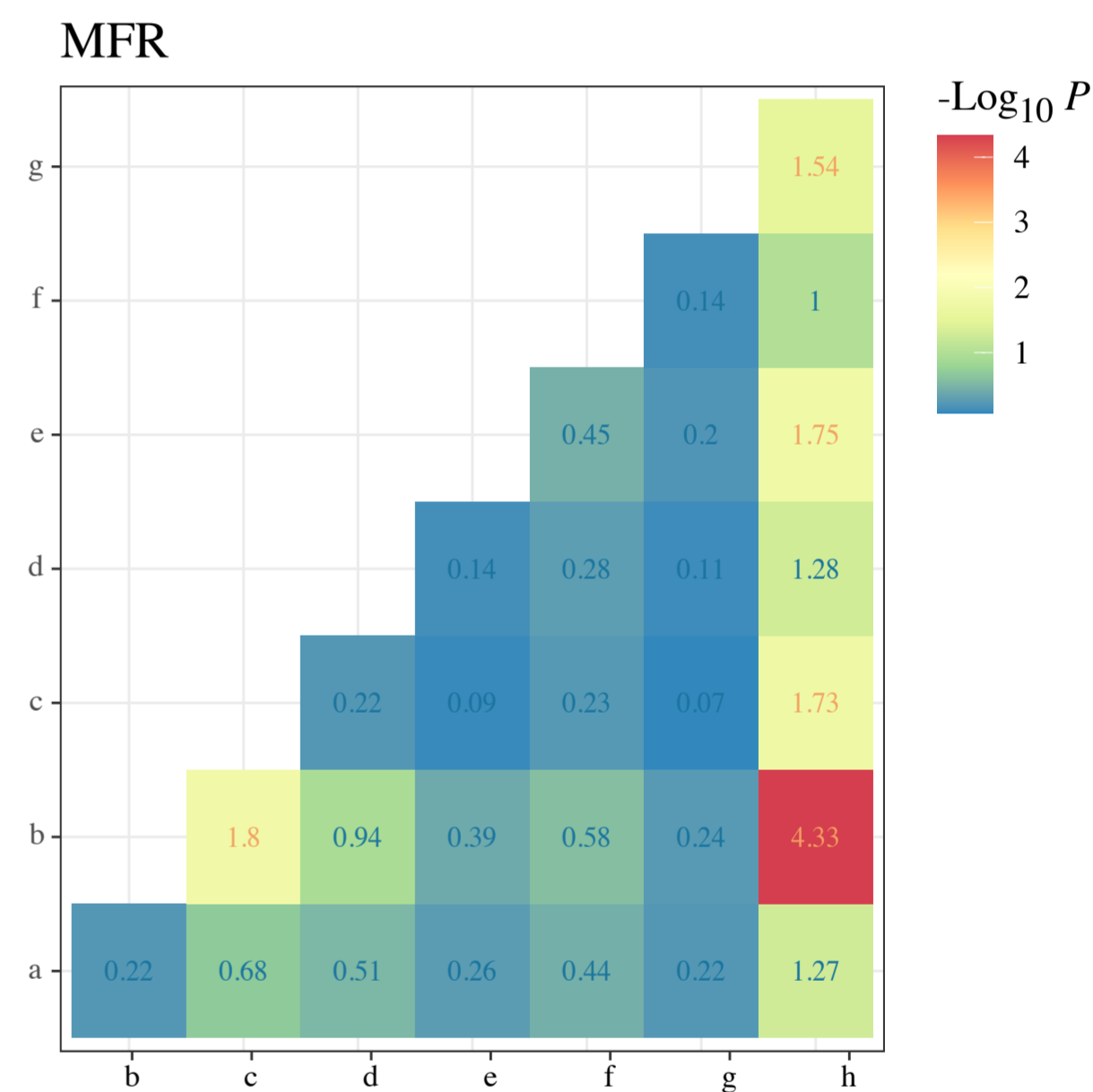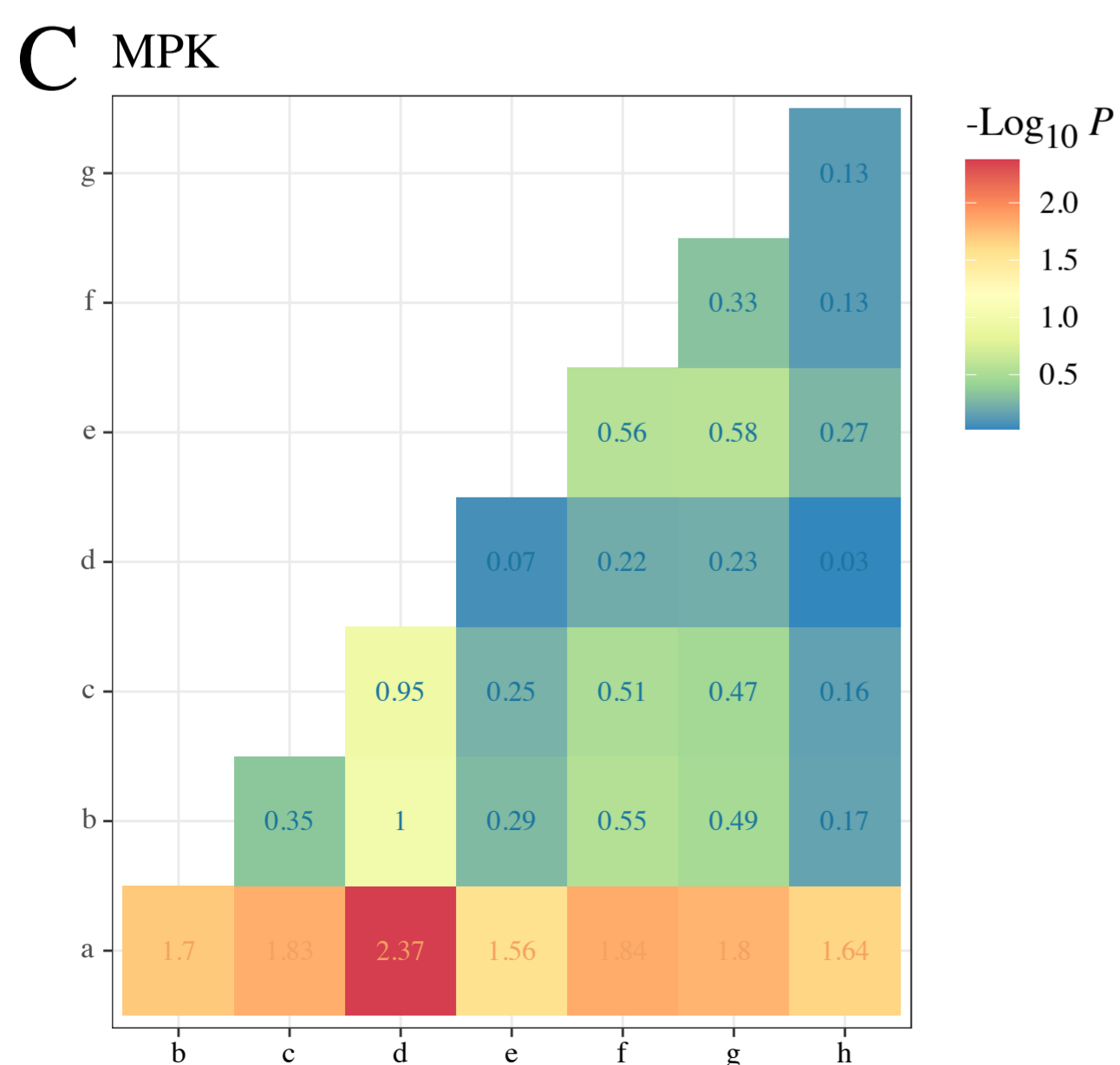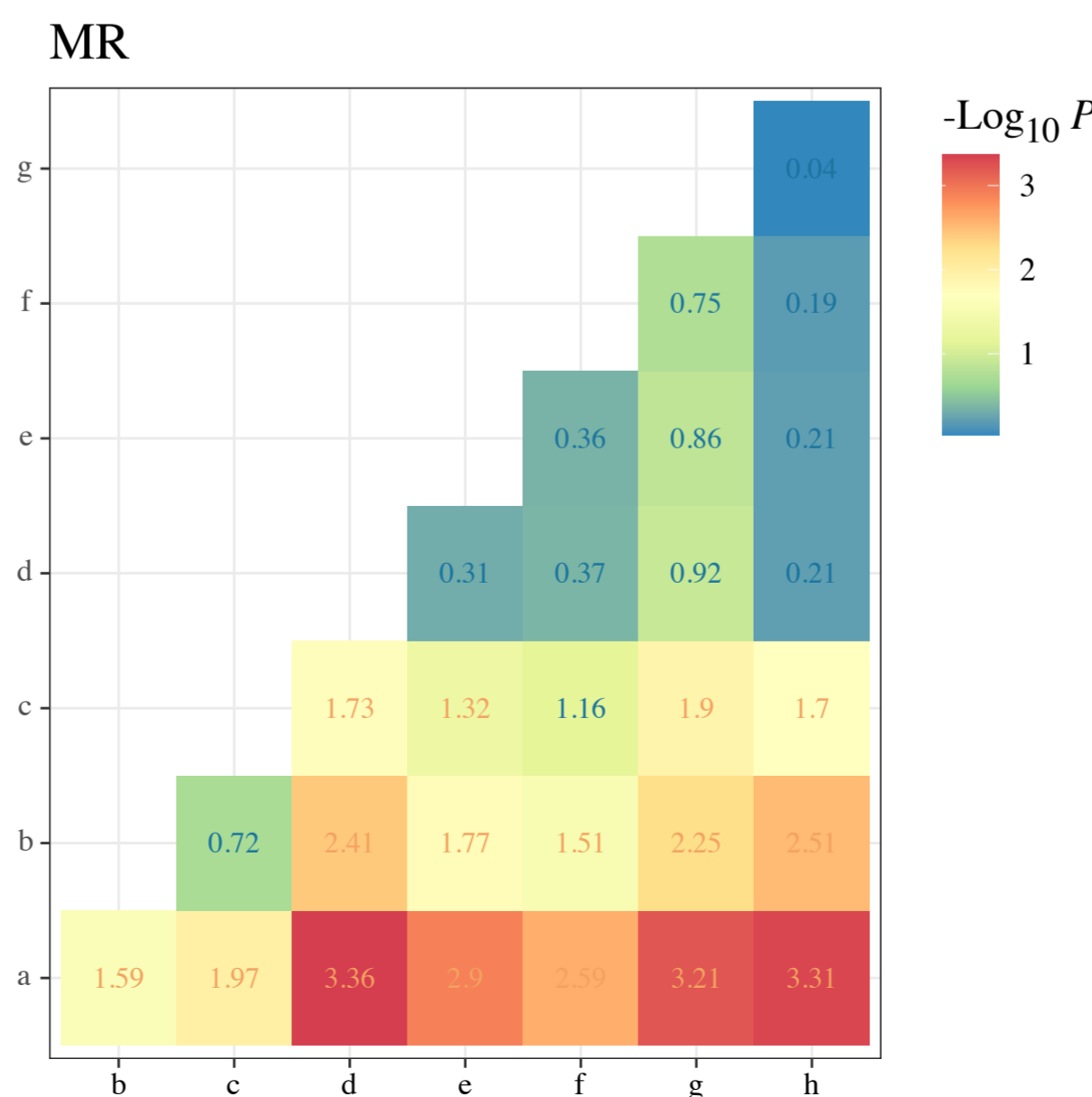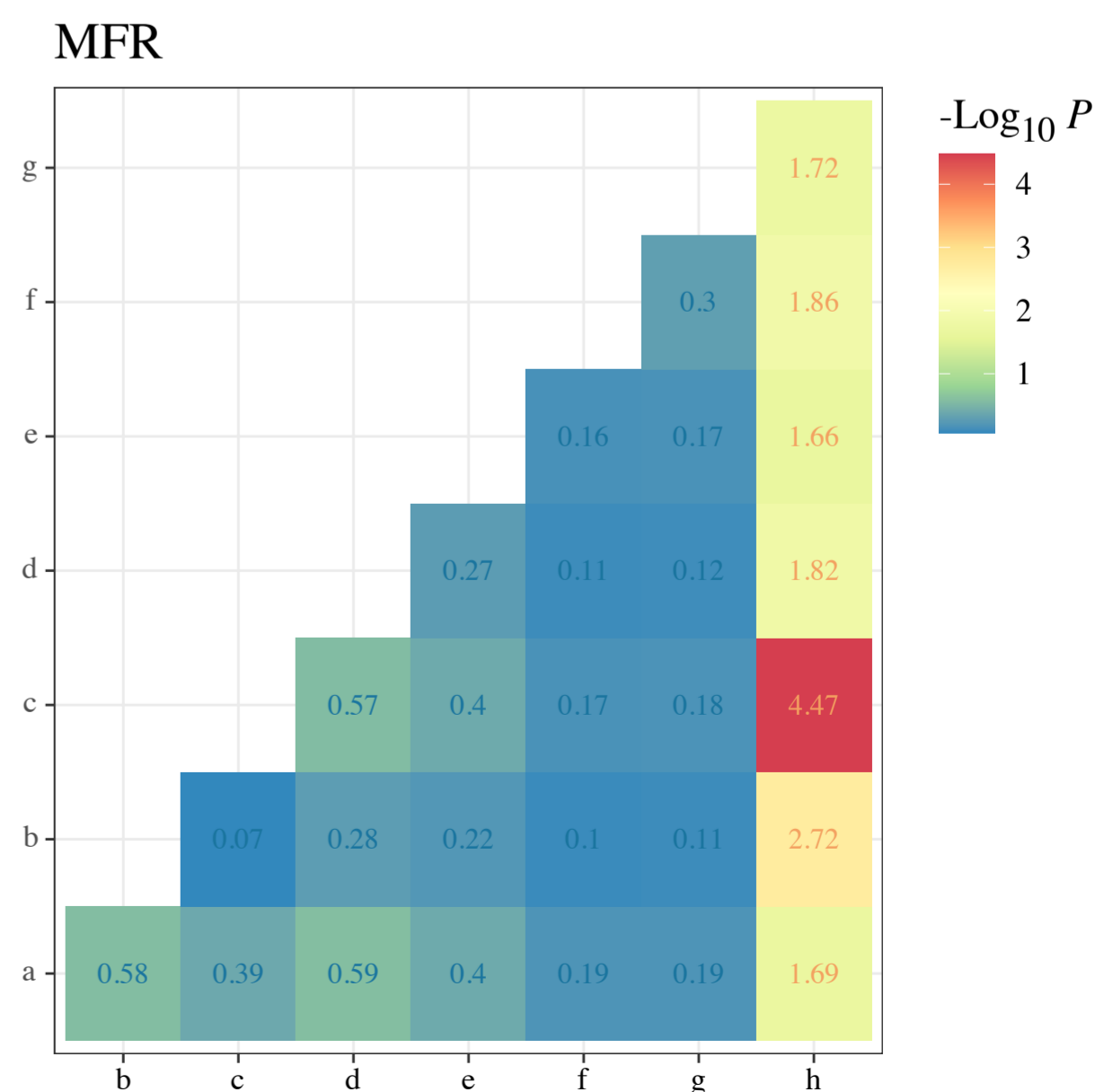

Supplement: Supplementary file 1 — Supporting File 1: advs76559‐sup‐0001‐SuppMatfiguresS1‐S21.zip [file ADVS-9999-e76559-s003.zip › S14.pdf]

A

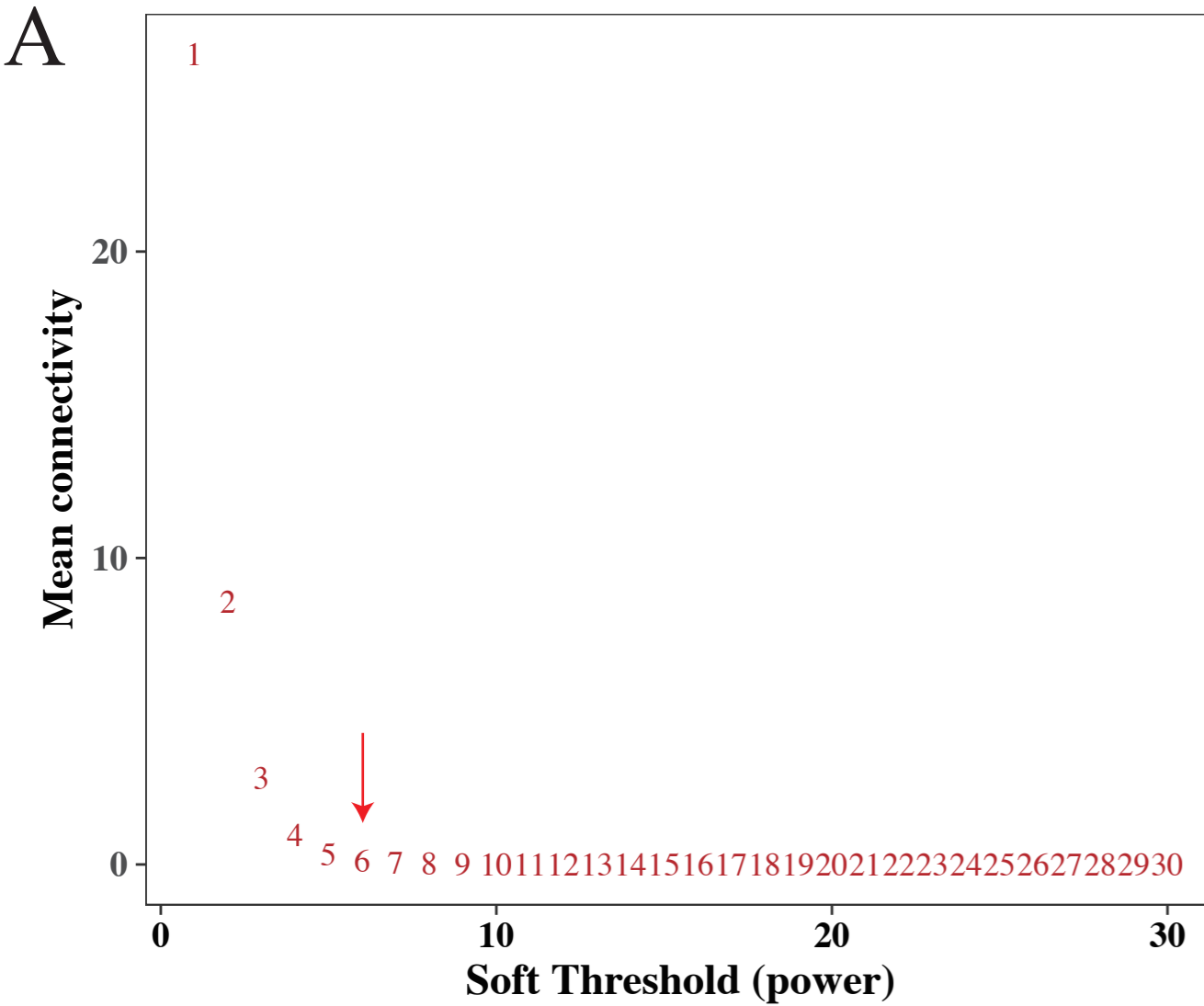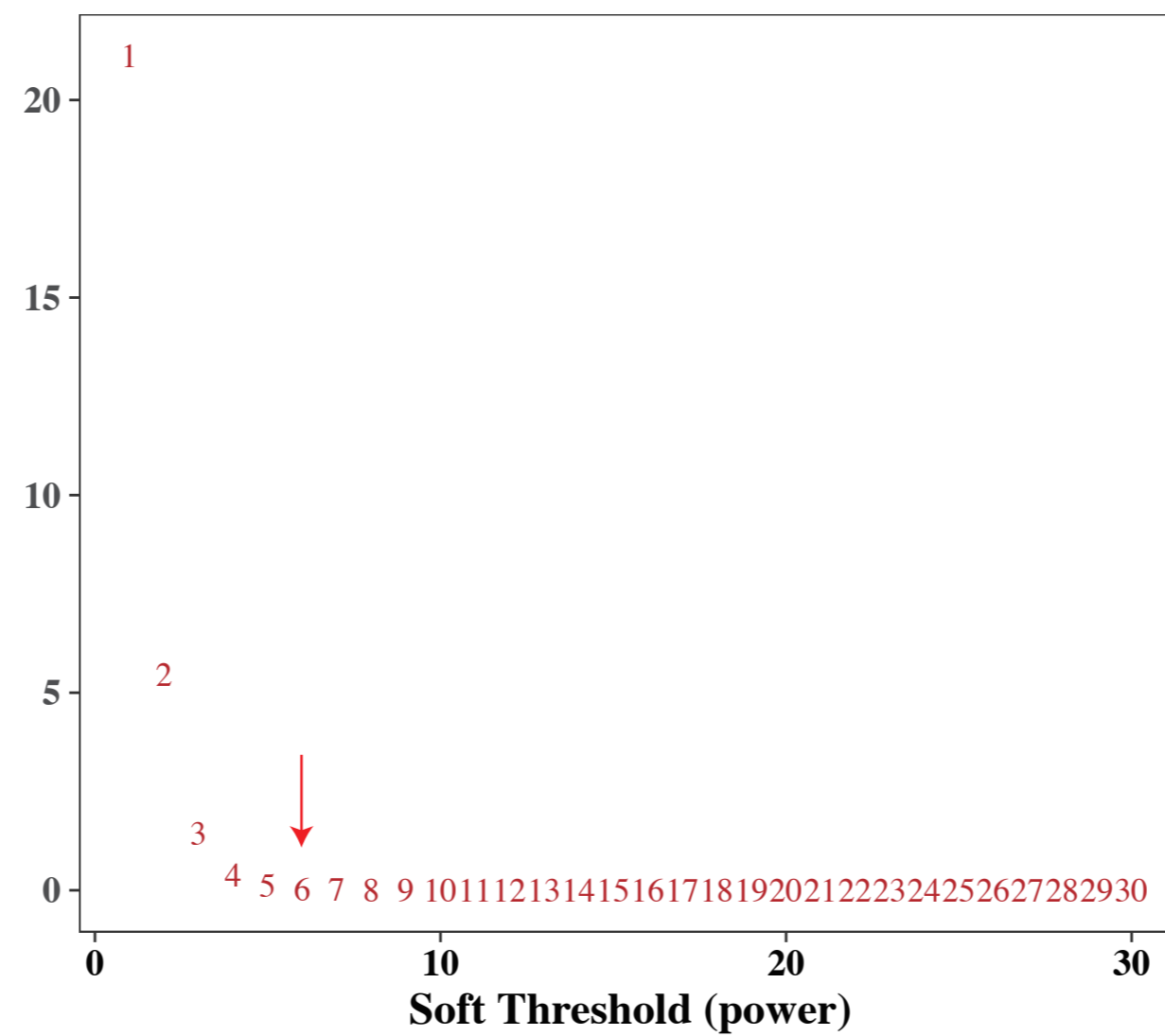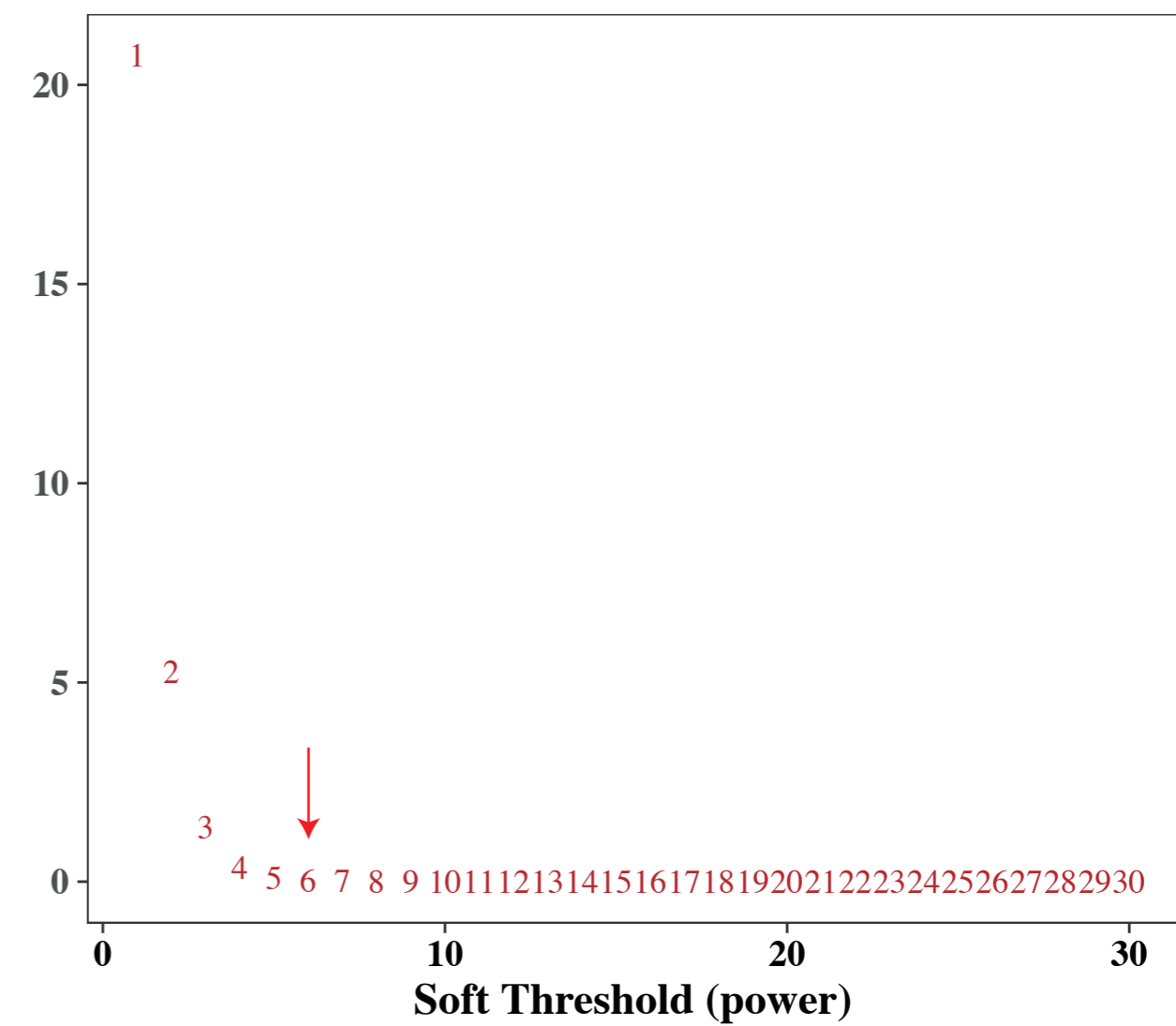

B

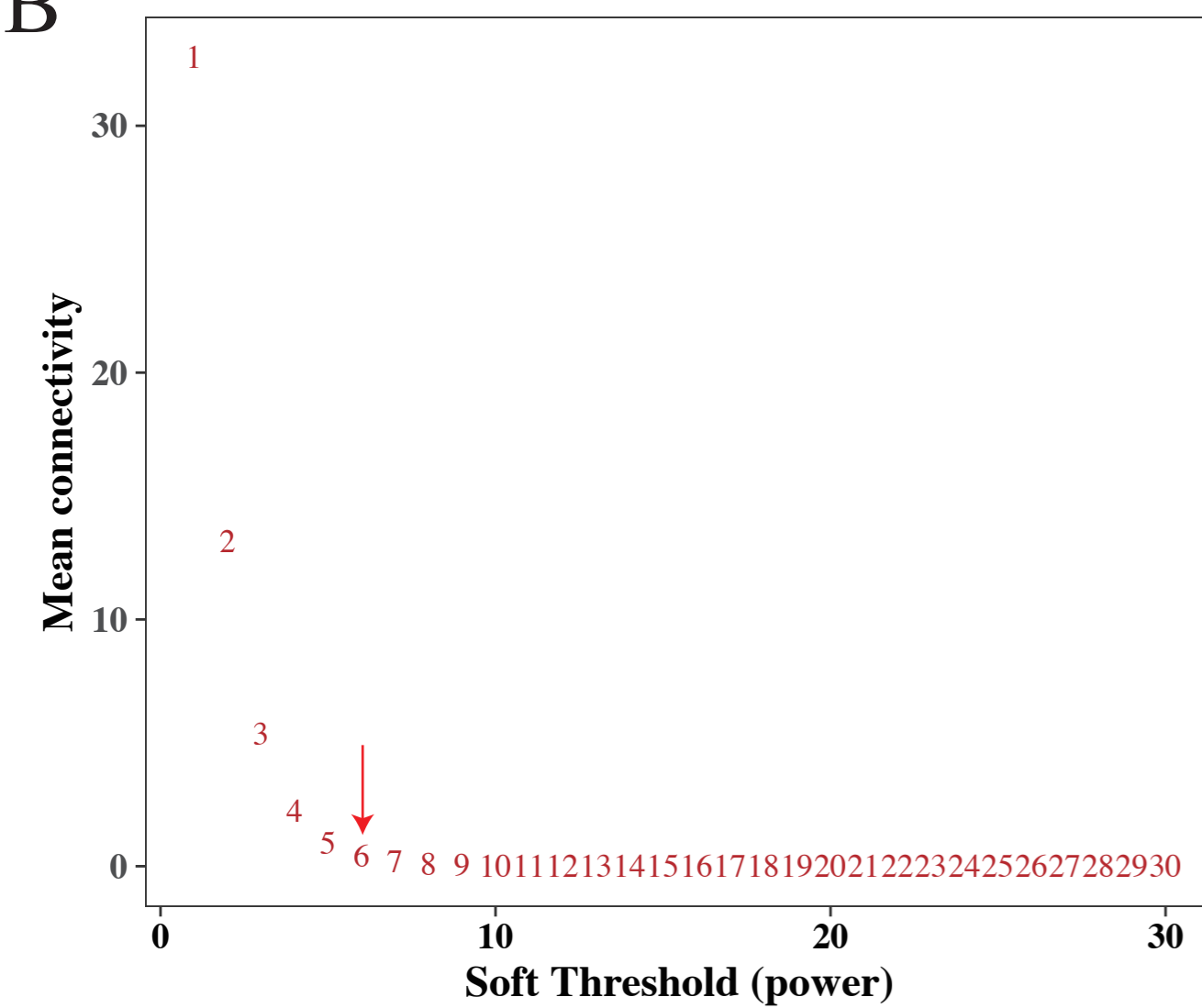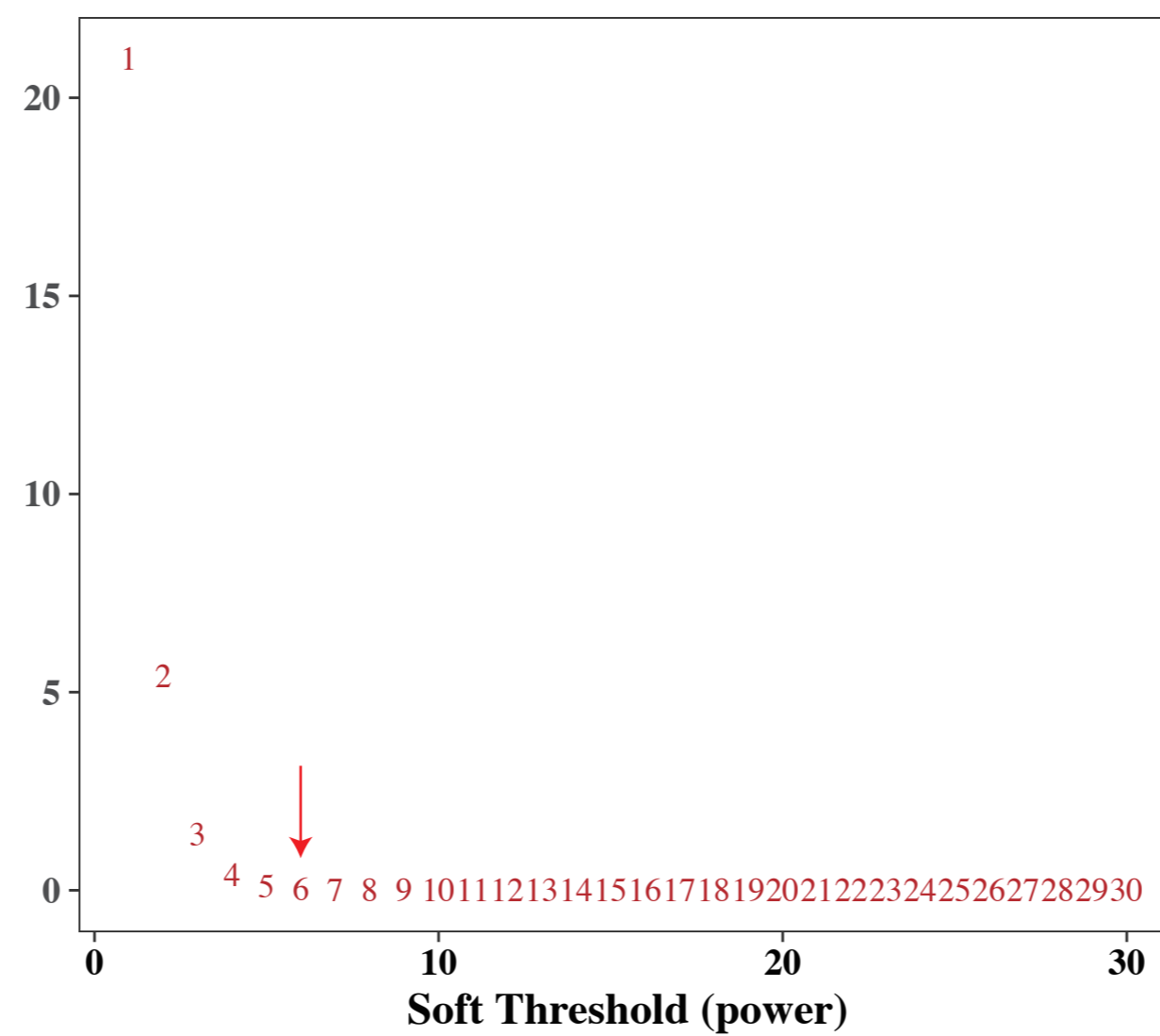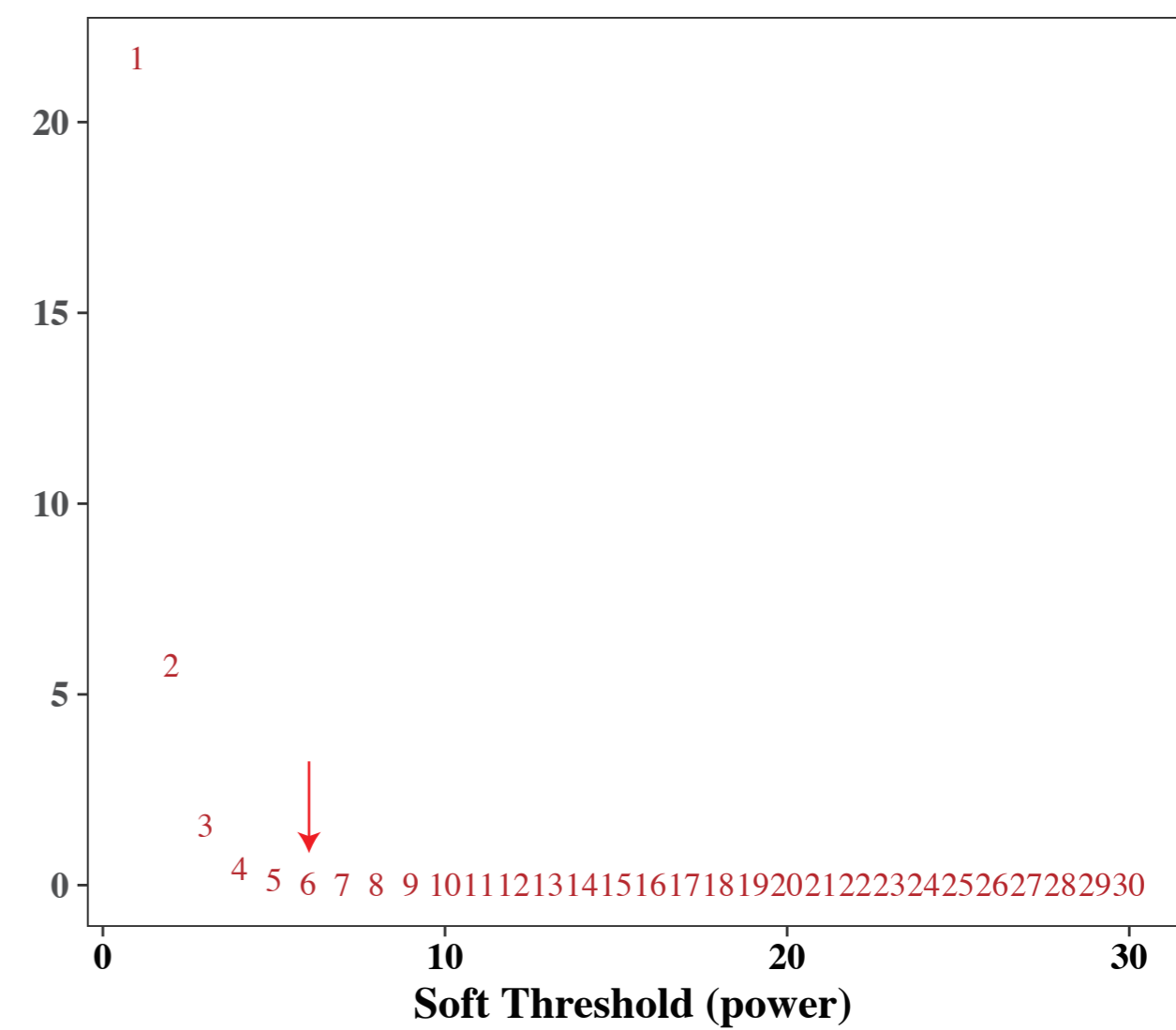

Supplement: Supplementary file 1 — Supporting File 1: advs76559‐sup‐0001‐SuppMatfiguresS1‐S21.zip [file ADVS-9999-e76559-s003.zip › S15.pdf]

A

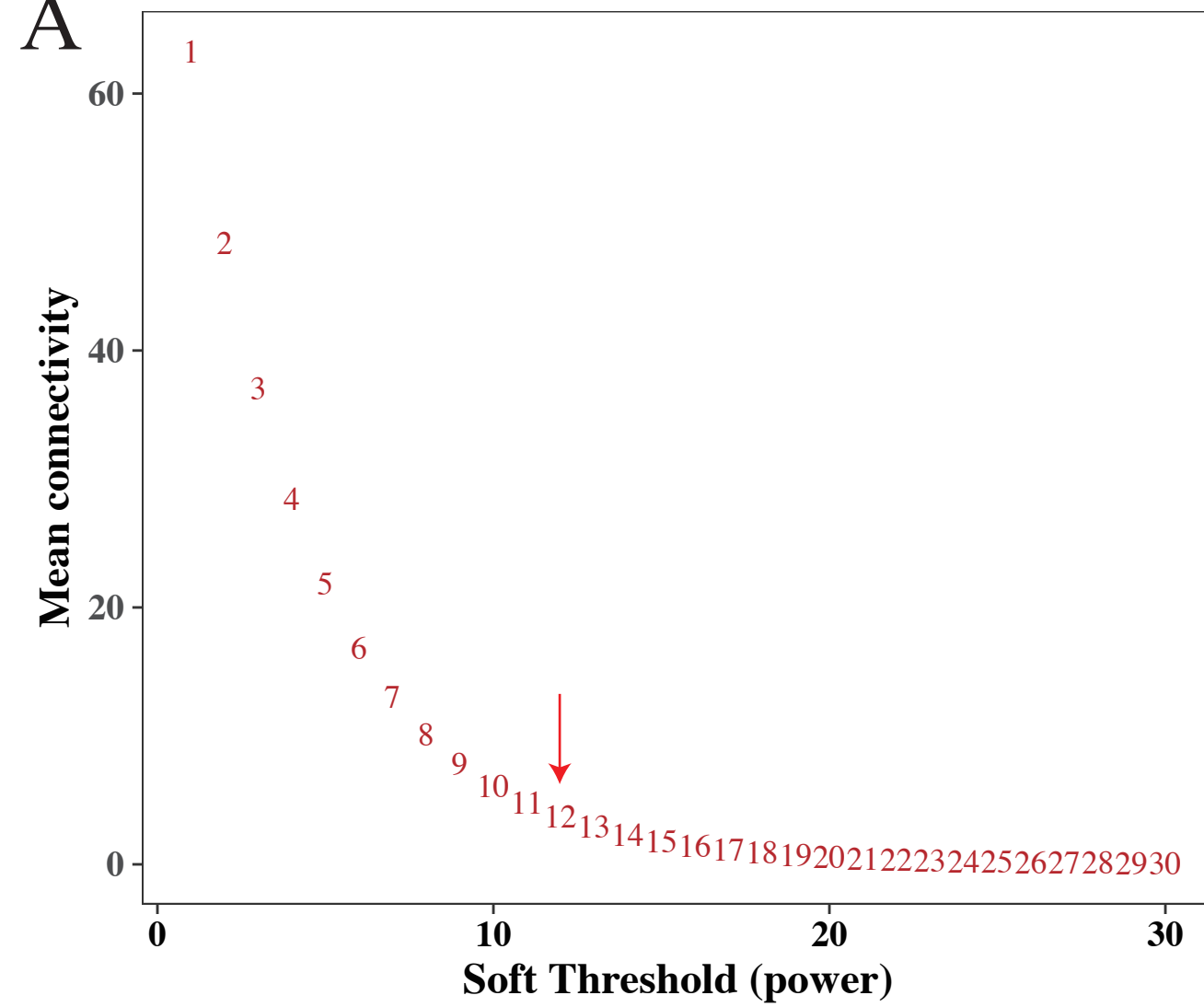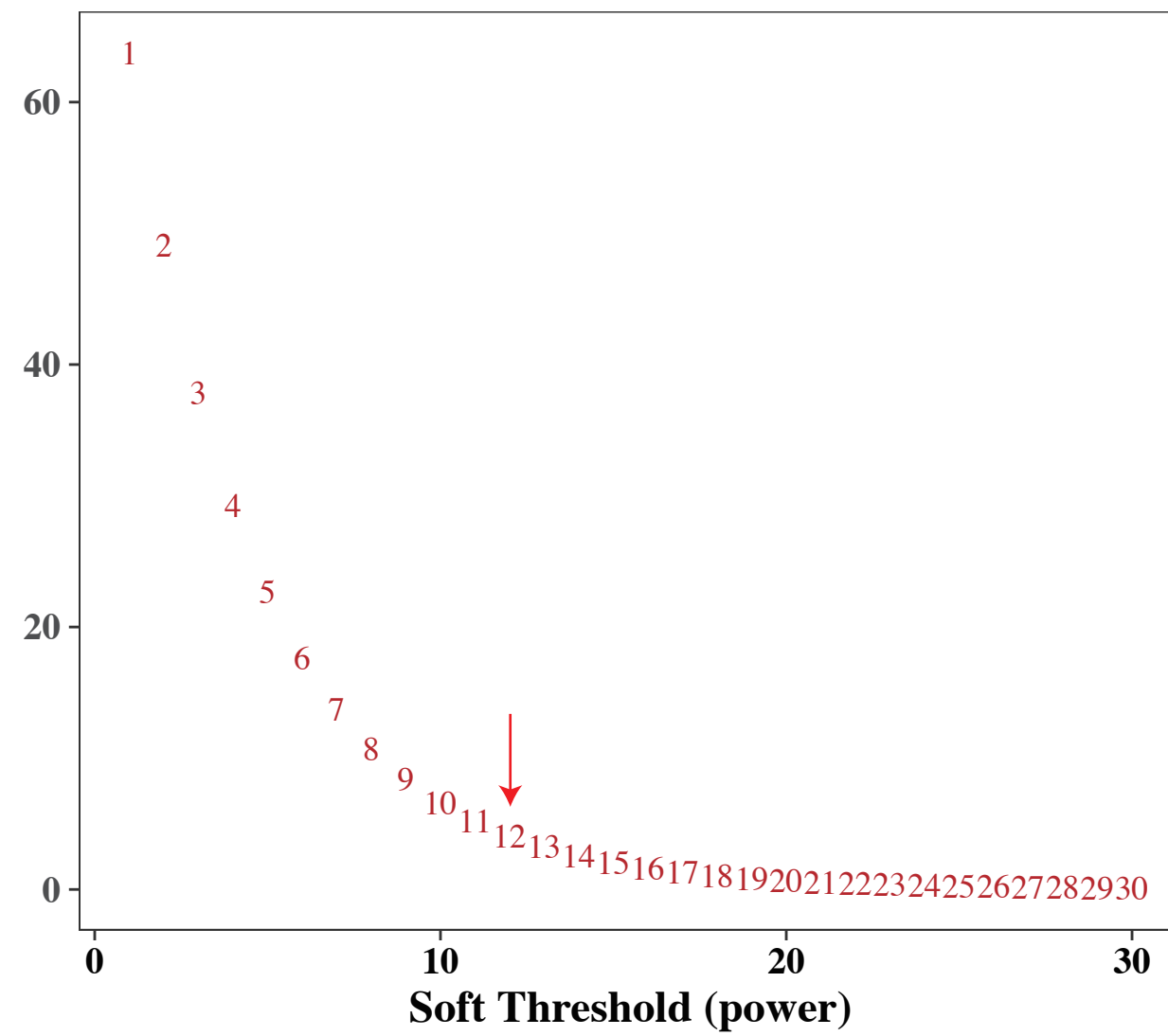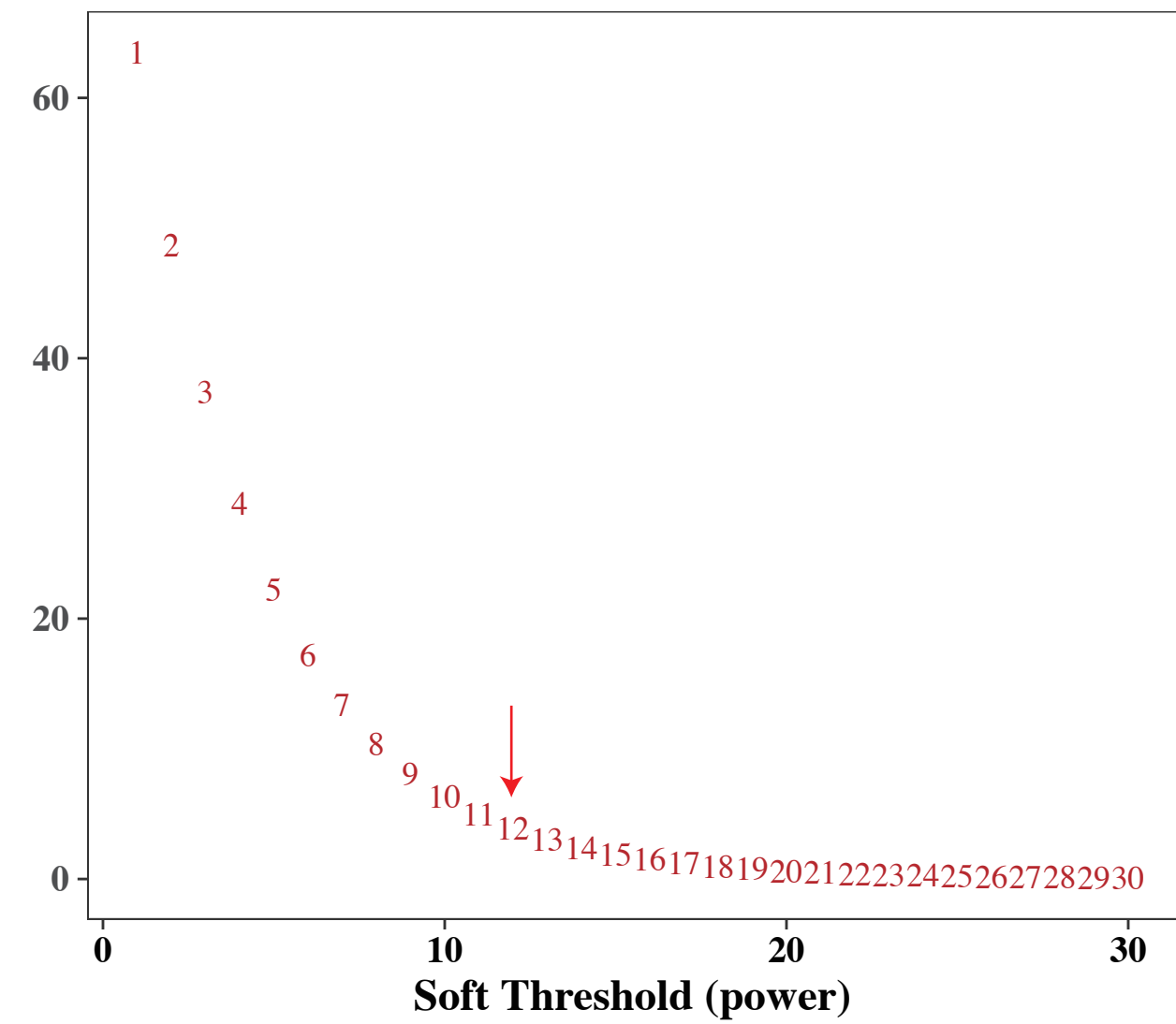

B

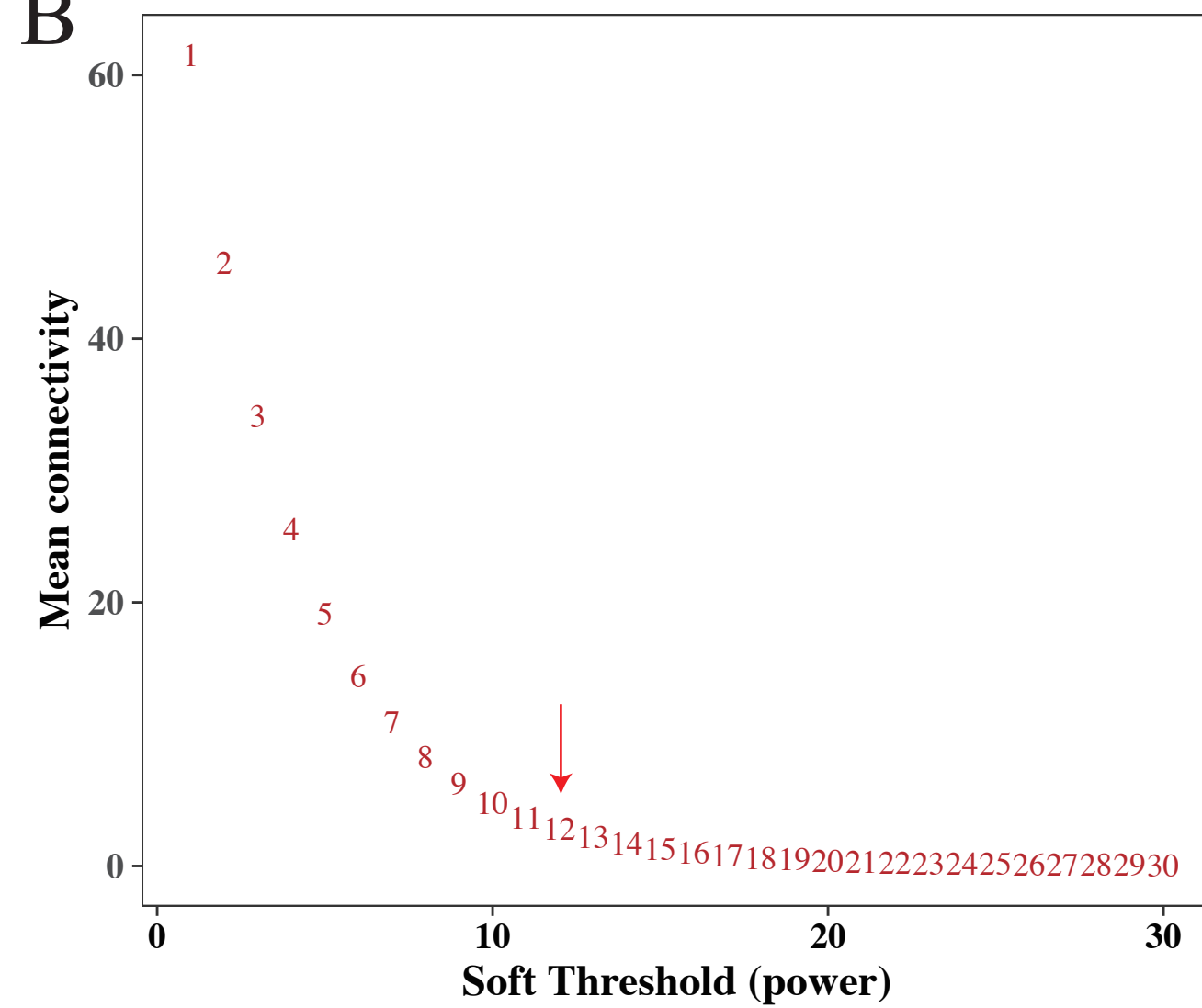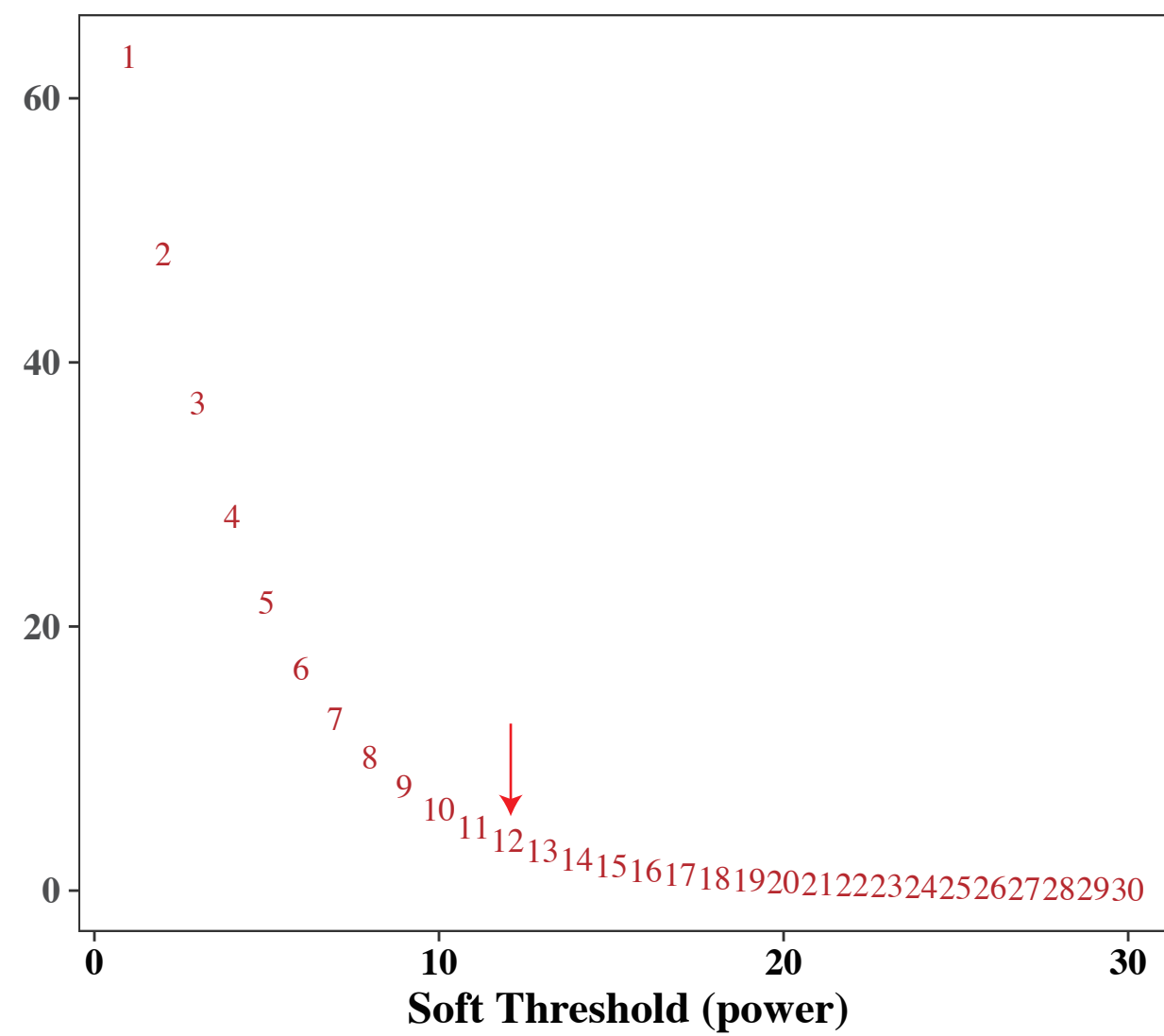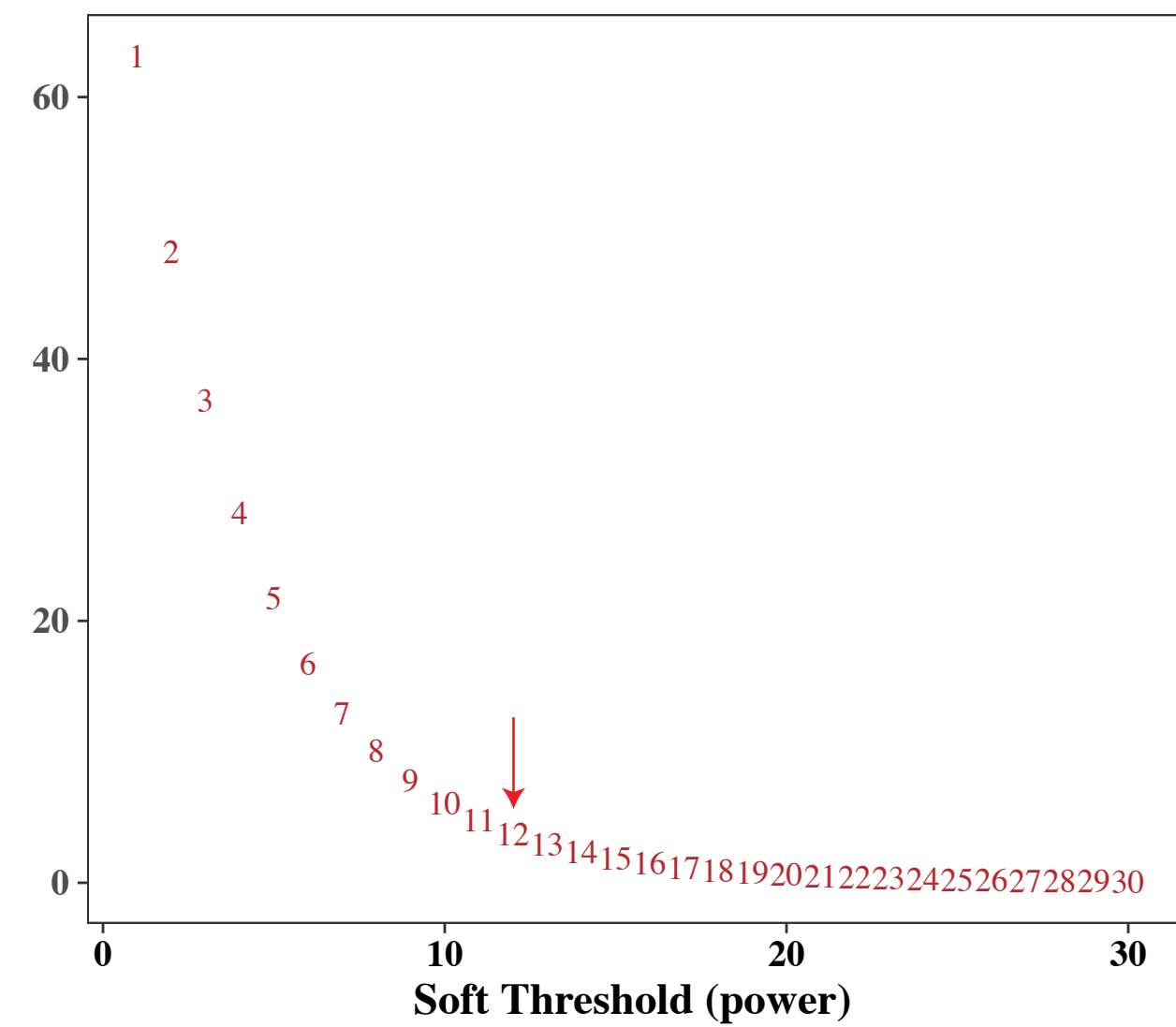

Supplement: Supplementary file 1 — Supporting File 1: advs76559‐sup‐0001‐SuppMatfiguresS1‐S21.zip [file ADVS-9999-e76559-s003.zip › S16.pdf]

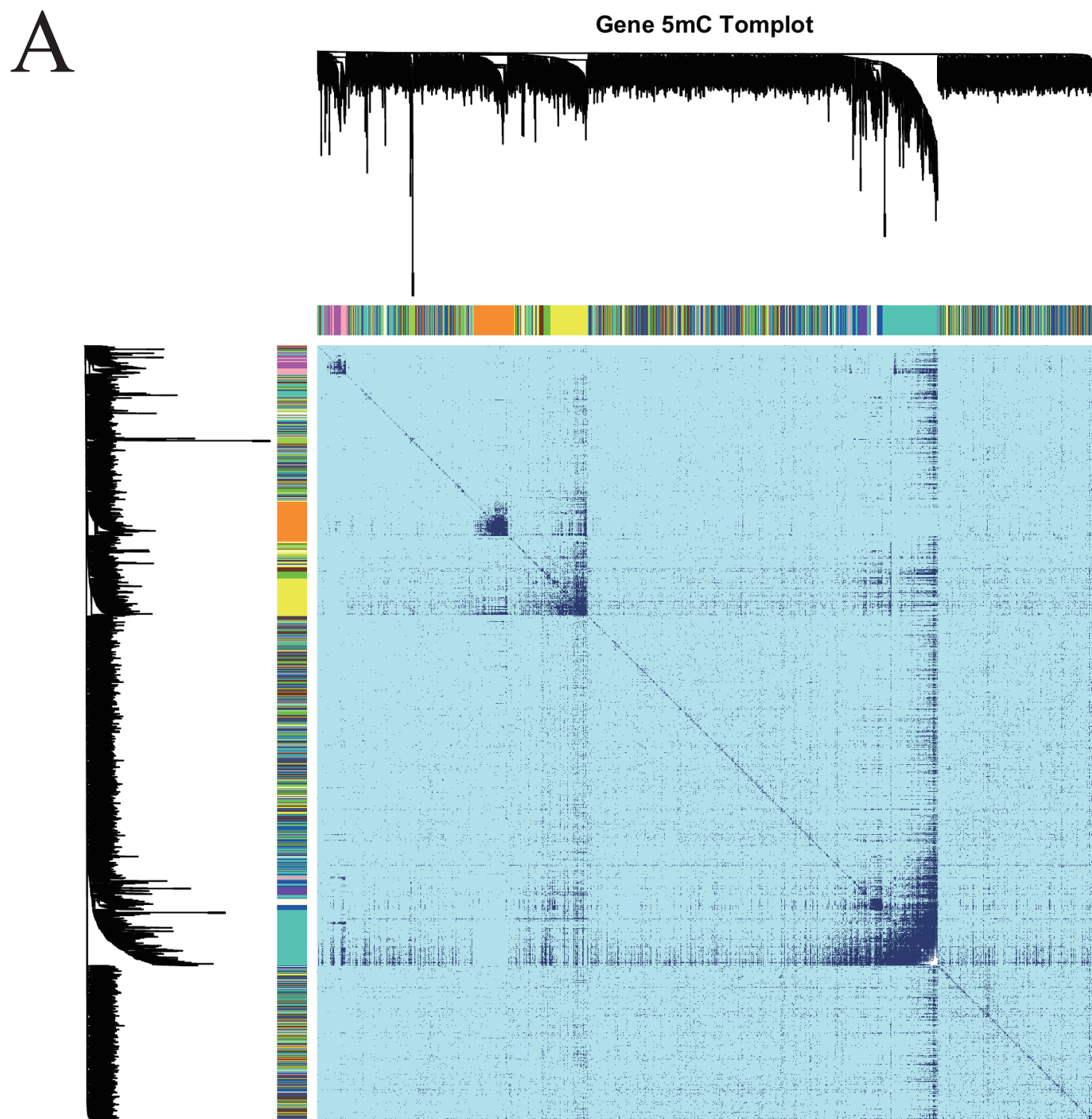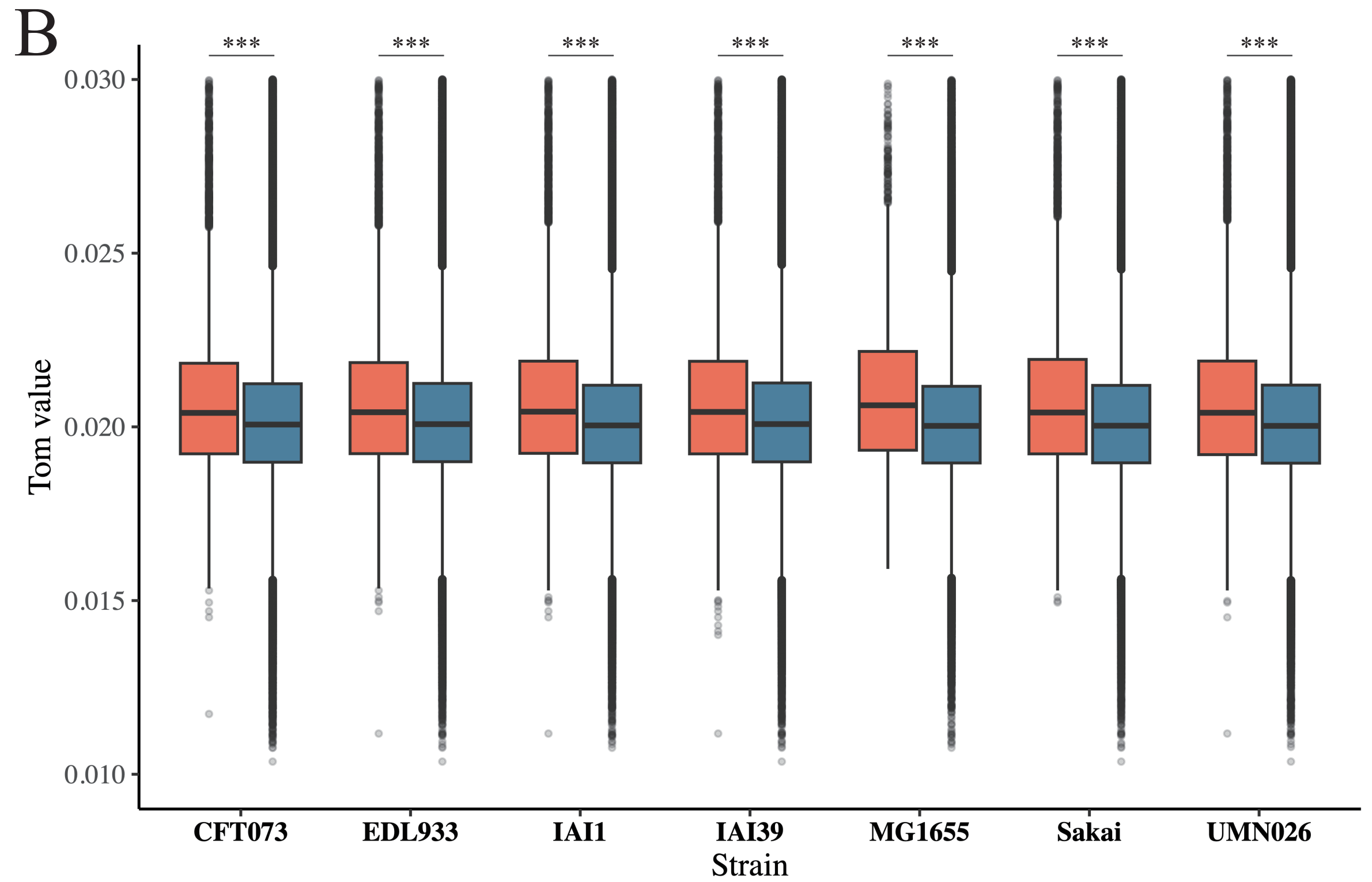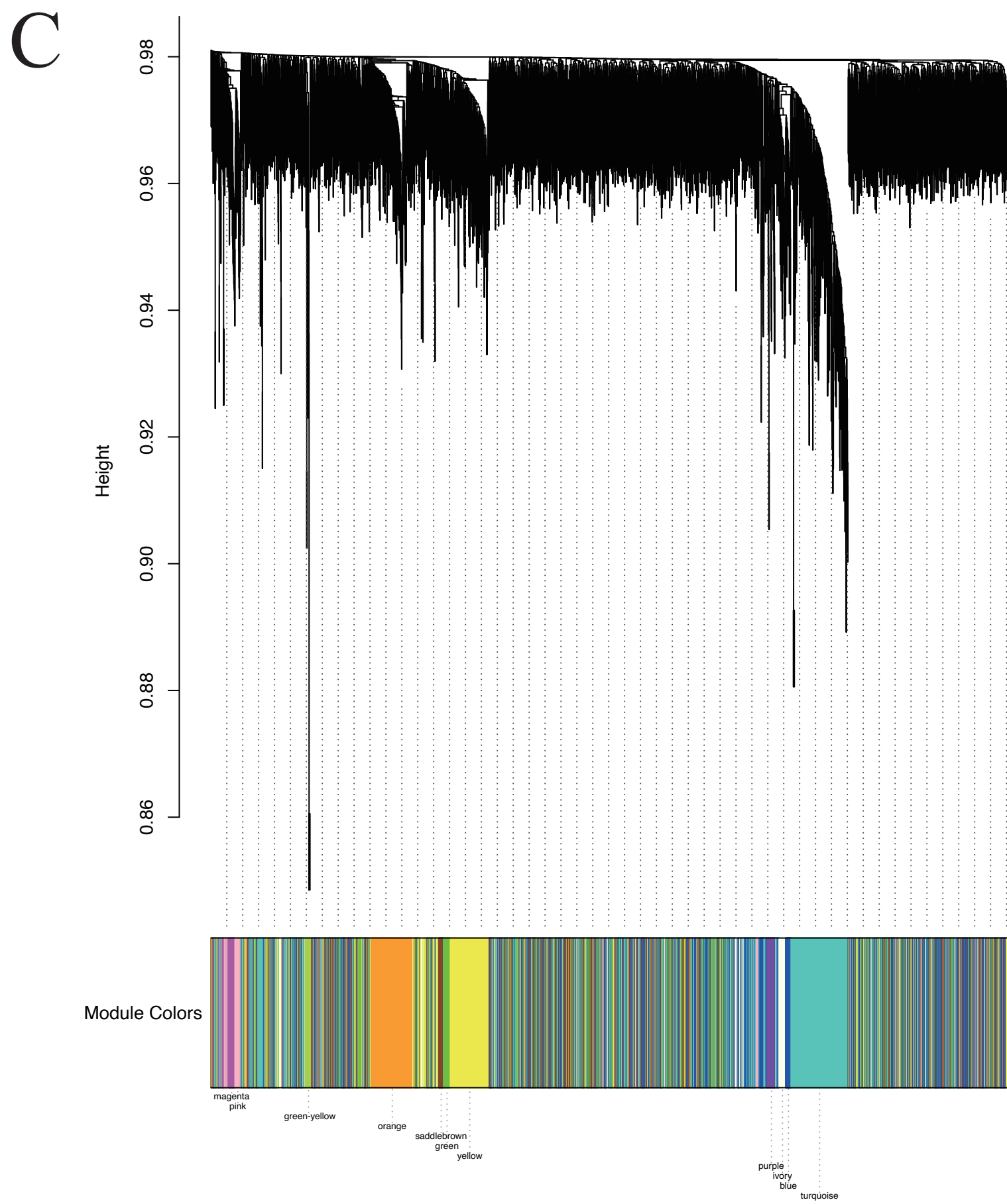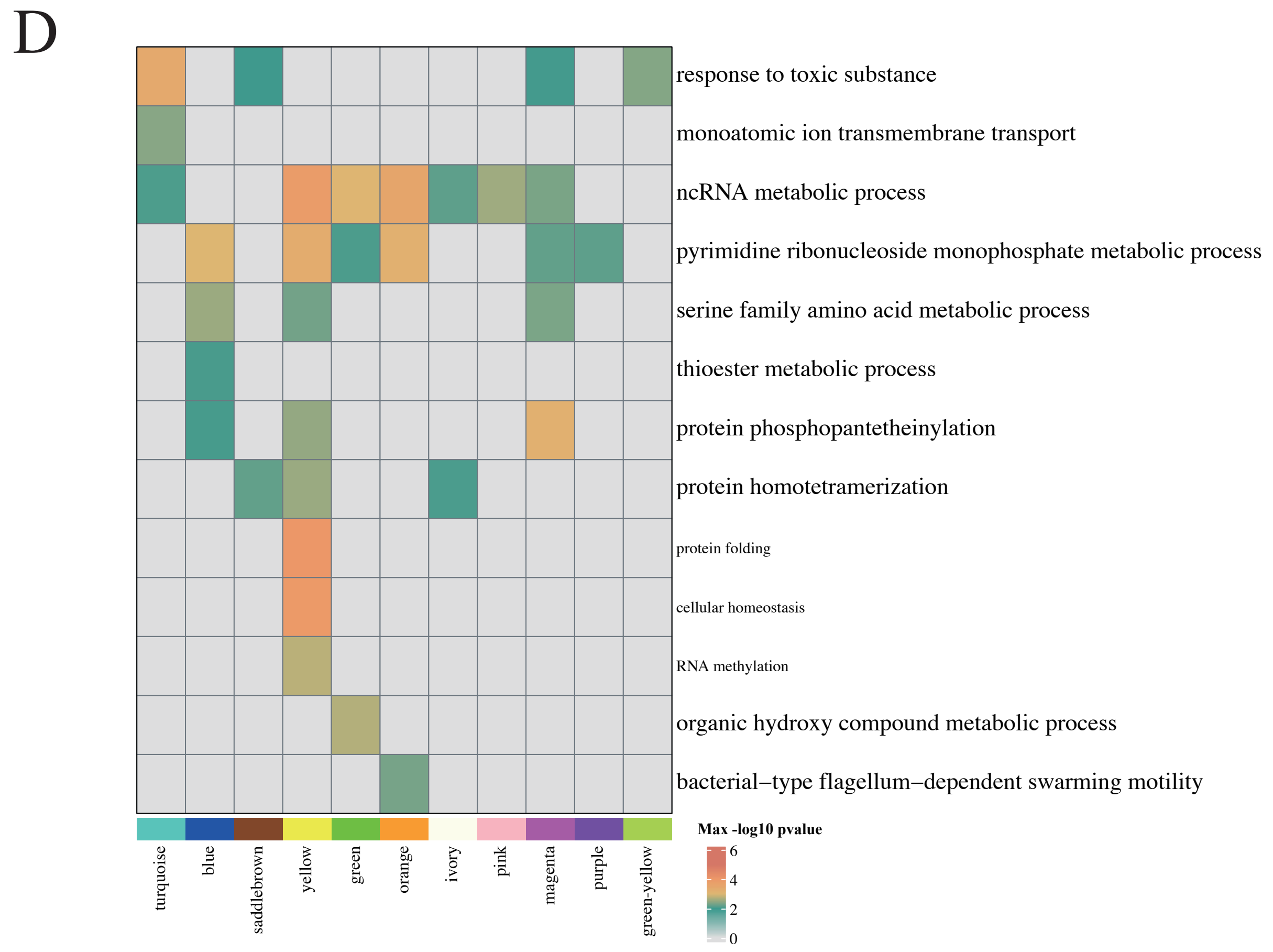

Supplement: Supplementary file 1 — Supporting File 1: advs76559‐sup‐0001‐SuppMatfiguresS1‐S21.zip [file ADVS-9999-e76559-s003.zip › S17.pdf]

A

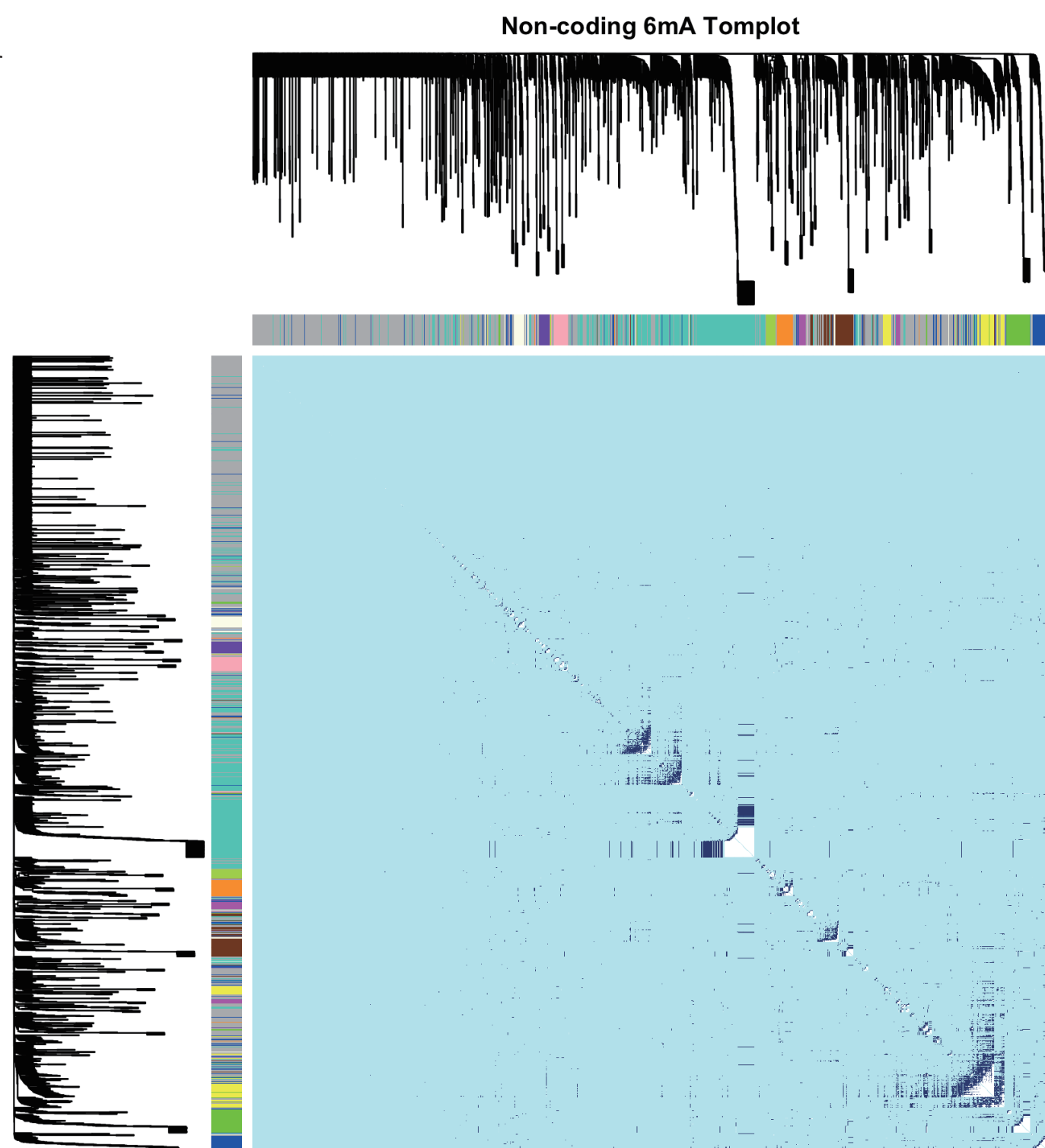

B

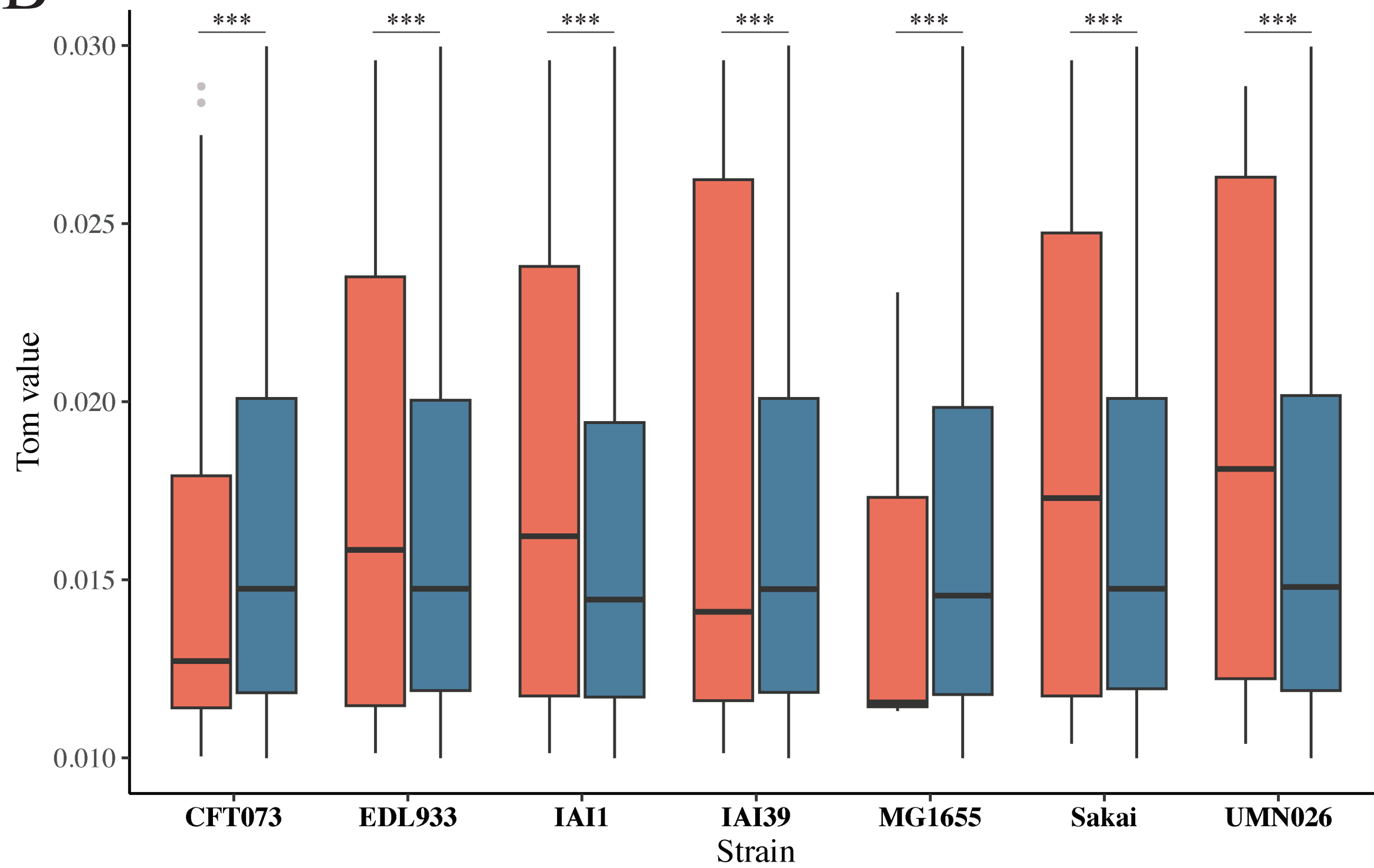

C

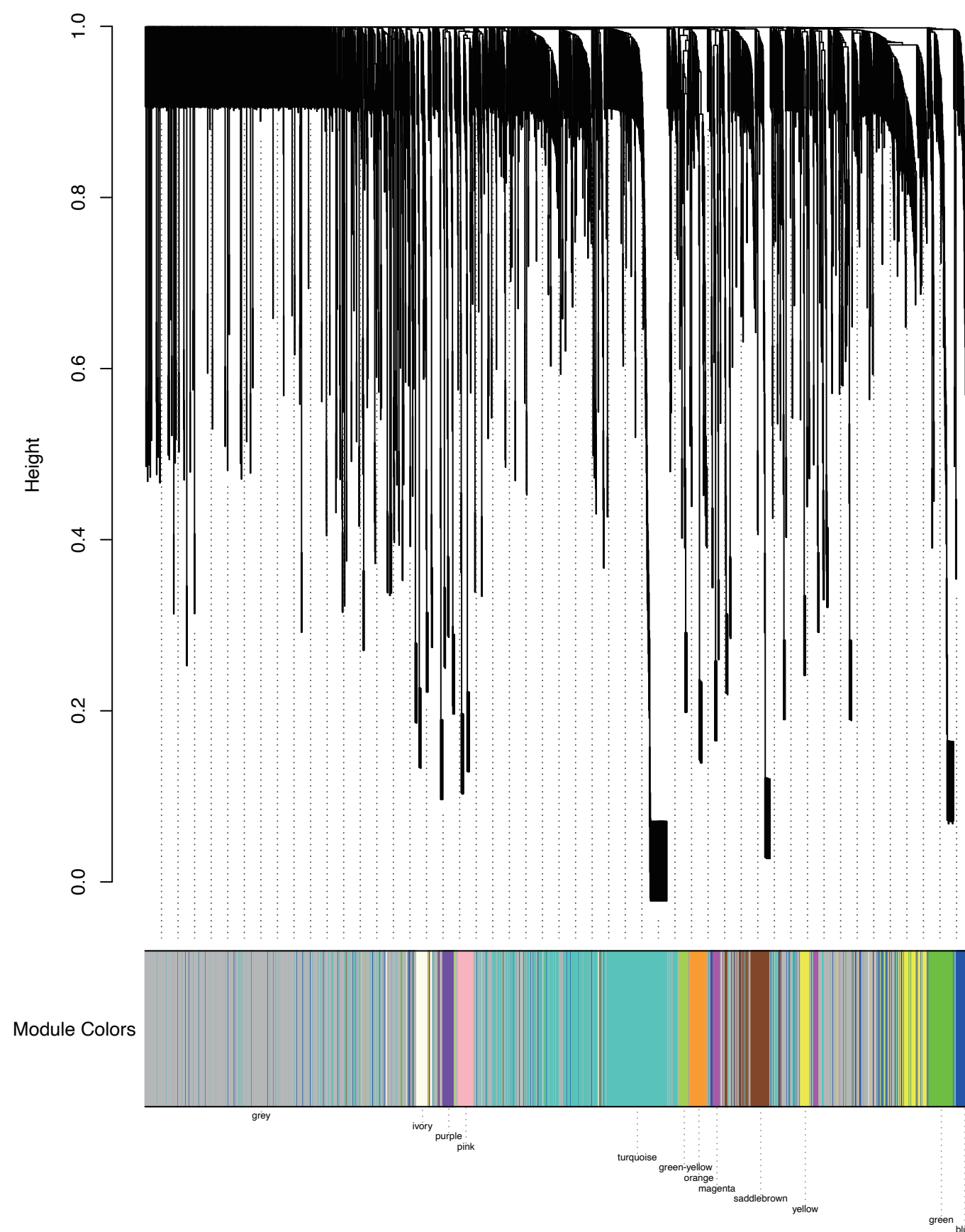

D

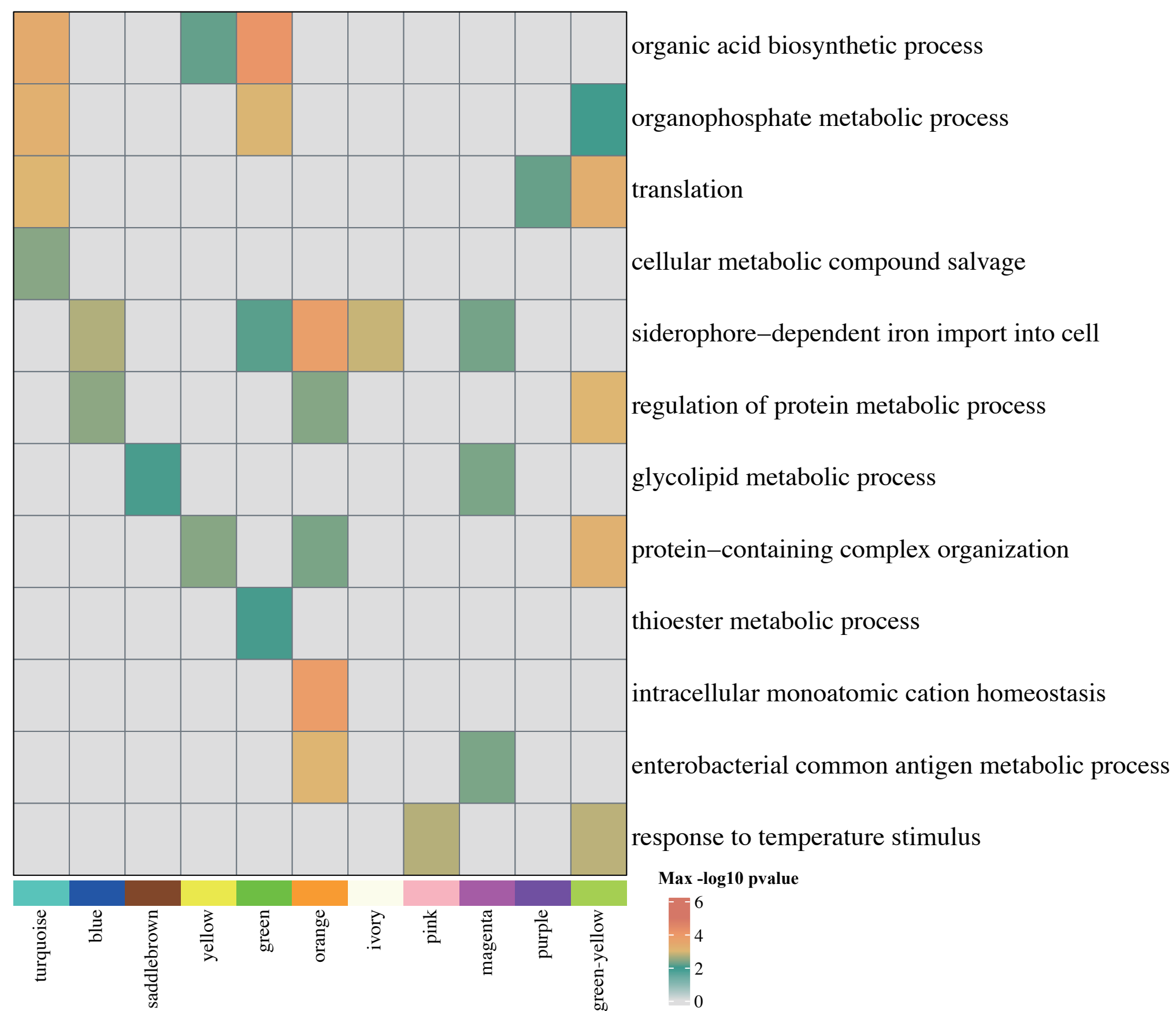

Supplement: Supplementary file 1 — Supporting File 1: advs76559‐sup‐0001‐SuppMatfiguresS1‐S21.zip [file ADVS-9999-e76559-s003.zip › S18.pdf]

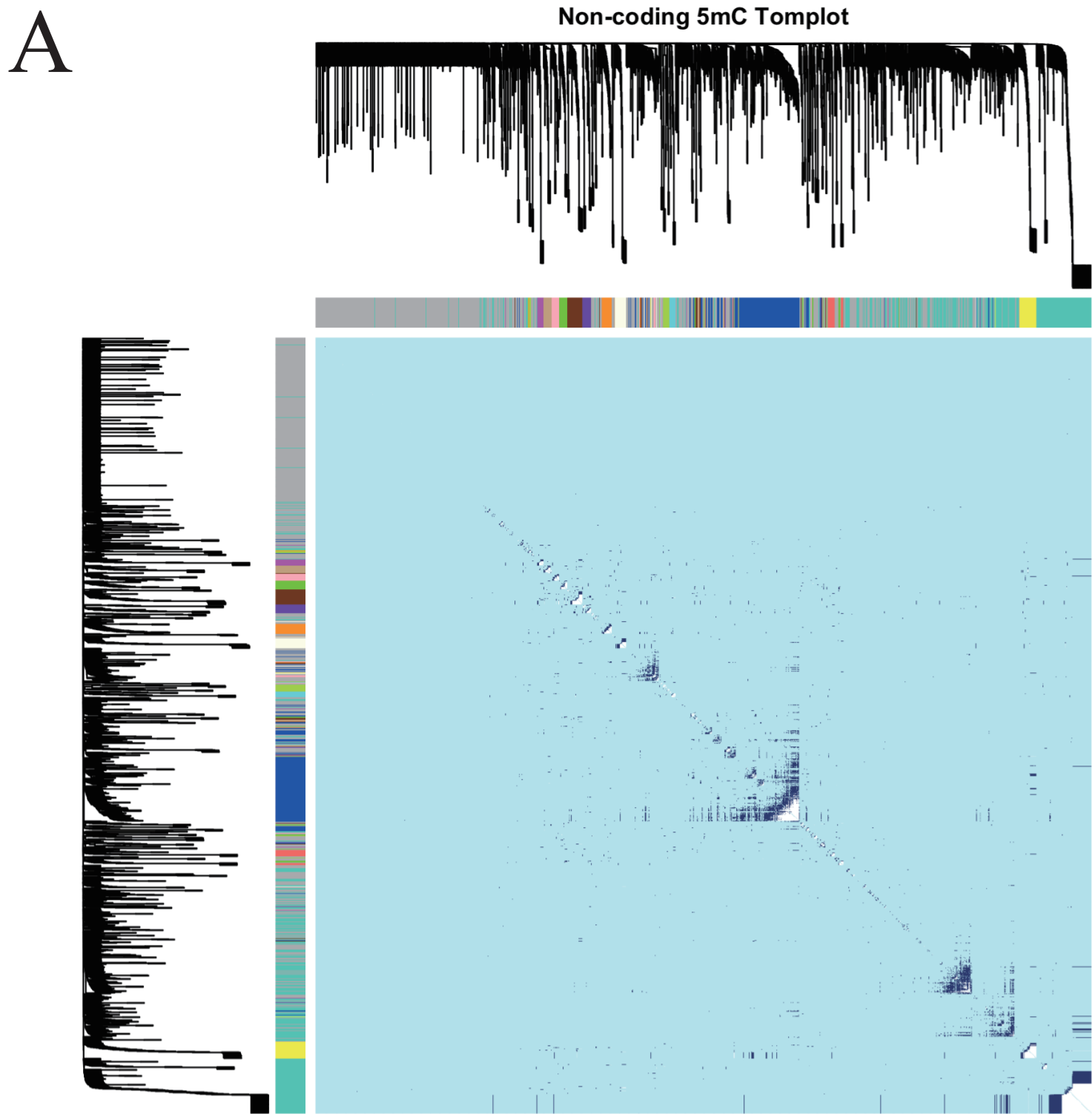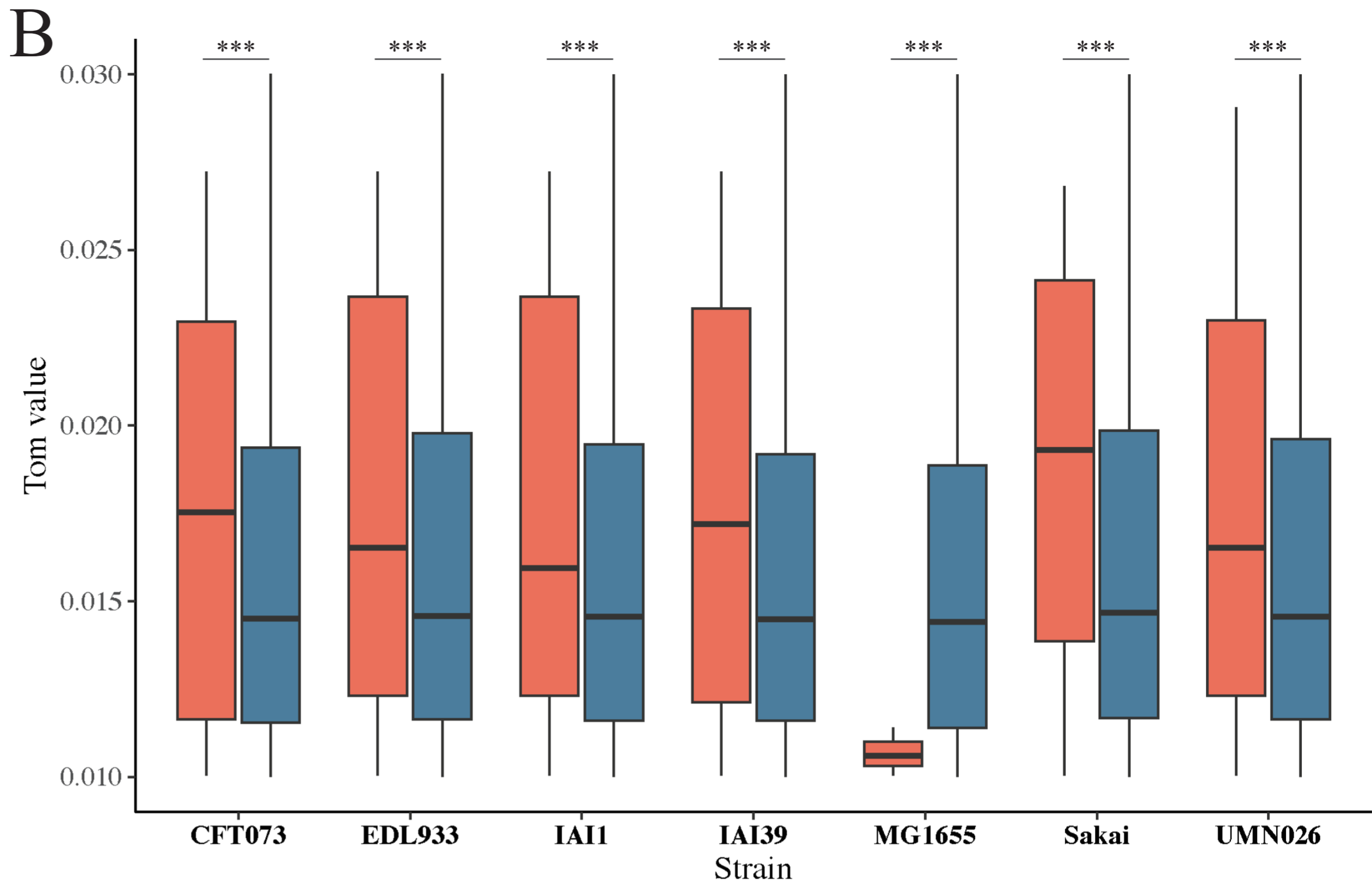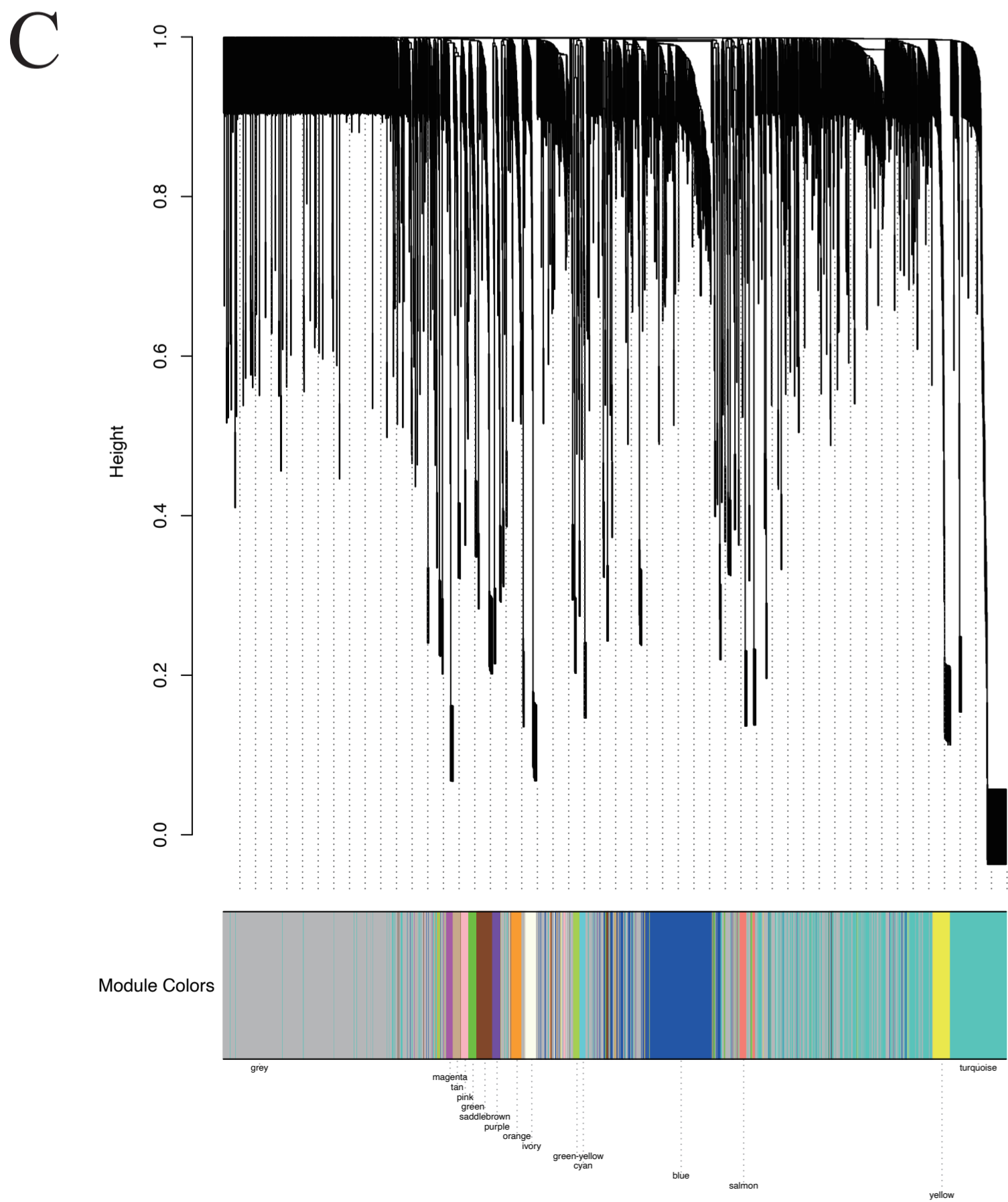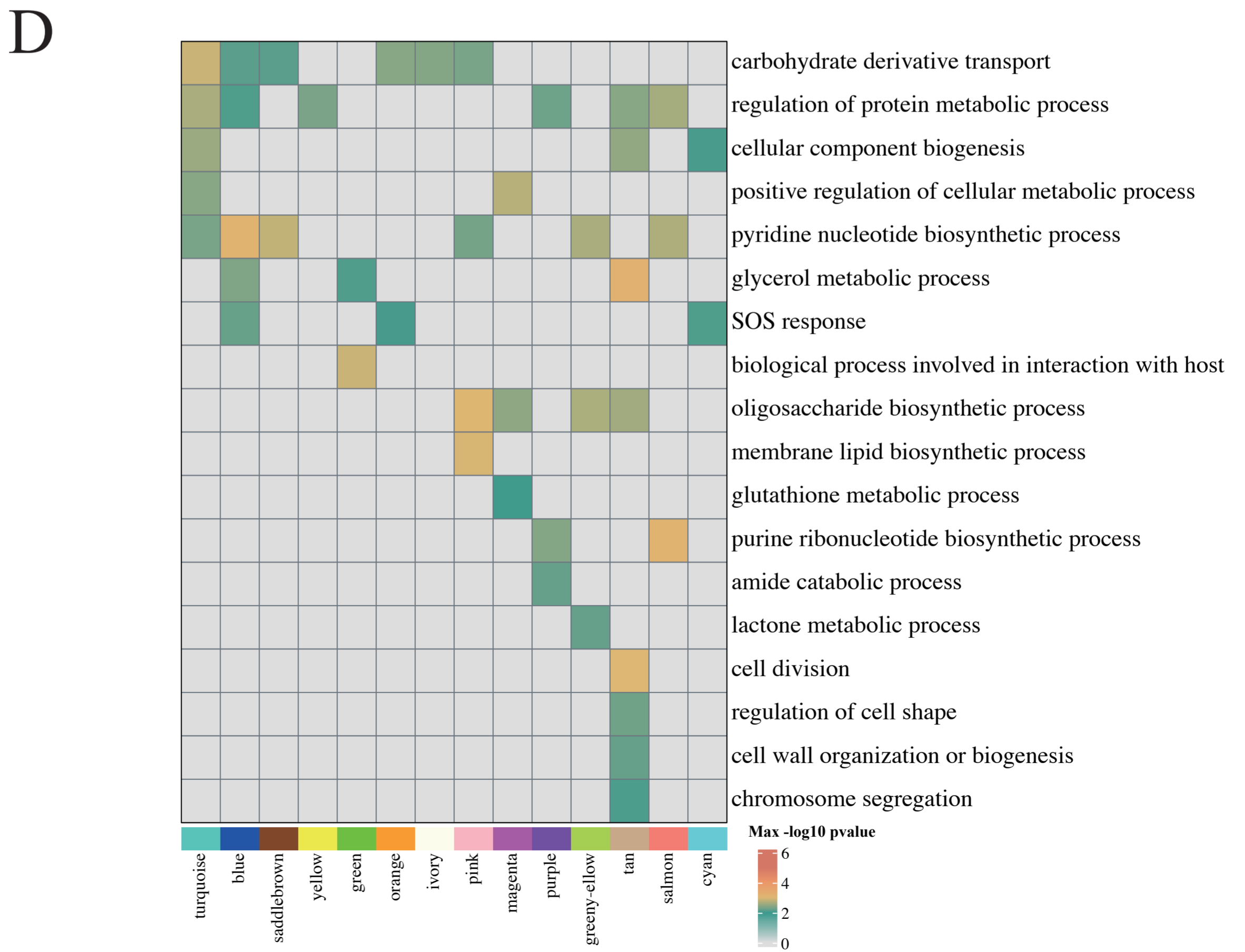

Supplement: Supplementary file 1 — Supporting File 1: advs76559‐sup‐0001‐SuppMatfiguresS1‐S21.zip [file ADVS-9999-e76559-s003.zip › S19.pdf]

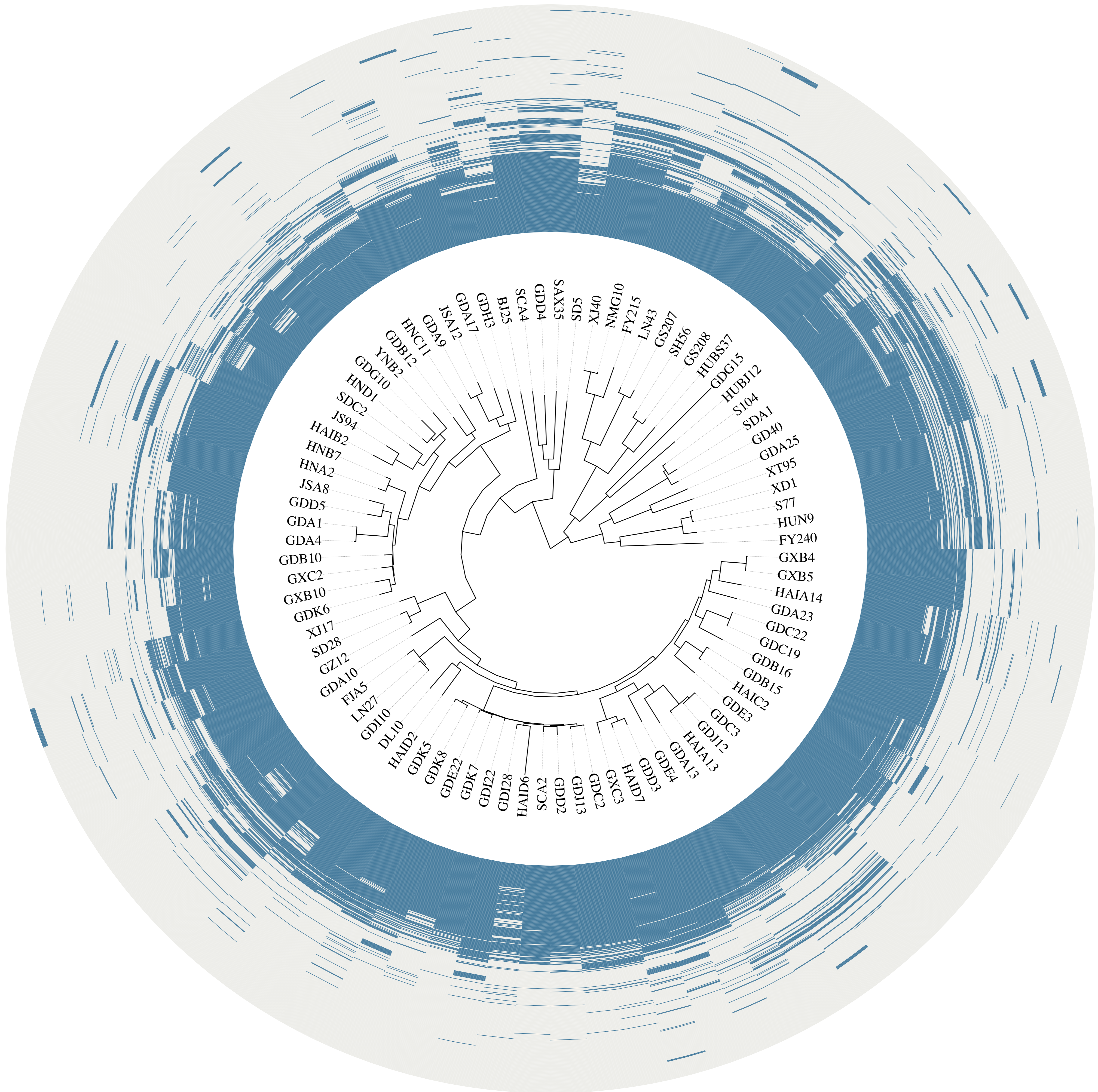

Supplement: Supplementary file 1 — Supporting File 1: advs76559‐sup‐0001‐SuppMatfiguresS1‐S21.zip [file ADVS-9999-e76559-s003.zip › S20.pdf]
