# Supplementary material for: A Pan‐Methylome Framework for Population‐Scale Bacterial Epigenomics
Source: Adv Sci (Weinh). 2026 Jul 13:e76559. Online ahead of print. doi: 10.1002/advs.76559 (PMC13360123; doi:10.1002/advs.76559)
Supplement: Supplementary file 2 — Supporting File 2: advs76559‐sup‐0002‐SuppMatfigurescaptions.docx. [file ADVS-9999-e76559-s001.docx]

Supplemental Figure 1. Pan-genome profile of 84 *E. coli* isolates. The pan-genome was constructed using Panaroo, with a phylogenetic tree generated from core gene SNPs using a maximum likelihood approach in IQ-TREE2.

Supplemental Figure 2. Motif prevalence selection. Presence/absence profiles of all motif types from the REBASE database across 84 *E. coli* strains.

Supplemental Figure 3. Functional enrichment of biological processes for genes with core methylation. Genes corresponding to core methylation sites were selected separately from Dam sense, Dam antisense, Dcm sense, and Dcm antisense strands. Gene Ontology (GO) biological process enrichment analysis was performed using the ClusterProfiler2. For each category, the top three biological processes with the lowest adjusted *P*-values were selected for display.

Supplemental Figure 4. Relationship between sequencing depth and the number of methylation sites. Methylation counts were calculated separately for Dam sense, Dam antisense, Dcm sense, and Dcm antisense strands. Linear regression analysis was performed to assess the relationship between sequencing coverage and the number of methylation sites across 84 *E. coli* strains.

Supplemental Figure 5. Methylation frequency and Observed / Expected (O/E) ratios in gene and non-coding regions. (A) The number and proportion of methylation events, motifs present but unmethylated, and motifs absent in the sense and antisense strands of gene and non-coding regions across 84 *E. coli* strains. (B) The O/E ratio in the sense and antisense strands of gene and non-coding regions. The two upper panels show the zero-order observed/expected (O/E) ratios, and the two lower panels show the first-order O/E ratios. Group comparisons were performed using the one-sided Mann–Whitney U test. *P* < 0.05 is denoted by *, *P* < 0.01 by **, and *P* < 10^-4^ by ***.

Supplemental Figure 6. Pan-methylome of antisense strands in gene and non-coding regions. (A) The pan-methylome constructed from antisense Dam and antisense Dcm methylation in gene regions. (B) The pan-methylome constructed from antisense Dam and antisense Dcm methylation in non-coding regions. Different colors represent each type of methylation, motif, and non-motif.

Supplemental Figure 7. Phylogroup composition of epi-phylogroups Epi-I, Epi-II, and Epi-III. The phylogroup distribution within each epi-phylogroup (Epi-I, Epi-II, and Epi-III) is shown. Different phylogroups are represented by distinct colors.

Supplemental Figure 8. Threshold determination for identifying high-fitness *E. coli* strains under environmental stress. *E. coli* strains were classified as high- or low-fitness based on *P* values from two-sided paired t-tests comparing fitness under treatment versus control conditions. The x-axis represents a series of P-value thresholds, and the y-axis indicates the number of strains identified as high-fitness under each threshold. Red arrows indicate inflection points where the number of high-fitness strains increases sharply; these points were defined as the fitness-based thresholds for each environmental condition.

Supplemental Figure 9. Comparison of complete methylation and hemi-methylation (sense and antisense) in gene regions. Dam (A) and Dcm (B) methylation sites in gene regions were classified into three categories: complete methylation, sense-strand methylation, and antisense-strand methylation. The left panels show the classification of methylation types, and the right panels display their distributions across 84 *E. coli* strains. Statistical significance was determined using a two-sided Mann–Whitney U test. *P* < 0.05 is denoted by *, *P* < 0.01 by **, and *P* < 10^-4^ by ***.

Supplemental Figure 10. Comparison of complete methylation and hemi-methylation (sense and antisense) in non-coding regions. Dam (A) and Dcm (B) methylation sites in non-coding regions were classified into three categories: complete methylation, sense-strand methylation, and antisense-strand methylation. The left panels show the classification of methylation types, and the right panels display their distributions across 84 *E. coli* strains. Statistical significance was determined using a two-sided Mann–Whitney U test. *P* < 0.05 is denoted by *, *P* < 0.01 by **, and *P* < 10^-4^ by ***.

Supplemental Figure 11. The Dcm methylation quantification of gene regions is associated with gene prevalence and gene quasi-essentiality. (A) Dcm methylation of gene region profiles in core and accessory genes of 84 *E. coli* strains, showing the proportions of methylation, unmethylated motif, and missing motifs. (B) For Dcm methylation in gene regions, the distributions of methylation per kilobase (MPK), methylation ratio (MR), and motif frequency ratio (MFR) are shown across essential genes and genes with varying prevalence. (C) Identification of high-methylation core genes based on Dcm methylation in gene regions. Genes in the top 10% for MPK, MR, and MFR were intersected with the core genes shared by 84 *E. coli* strains, resulting in 11 high-methylation core genes.

Supplemental Figure 12. The Dam methylation quantification of non-coding regions is associated with gene prevalence and gene quasi-essentiality. (A) Dam methylation profiles of non-coding regions in core and accessory genes of 84 *E. coli* strains, showing the proportions of methylation, unmethylated motif, and missing motifs. (B) For Dam methylation in non-coding regions, the distributions of methylation per kilobase (MPK), methylation ratio (MR), and motif frequency ratio (MFR) are shown across essential genes and genes with varying prevalence. (C) Identification of high-methylation core genes based on Dam methylation in non-coding regions. Genes in the top 10% for MPK, MR, and MFR were intersected with the core genes shared by 84 *E. coli* strains, resulting in 28 high-methylation core genes.

Supplemental Figure 13. The Dcm methylation quantification of non-coding regions is associated with gene prevalence and gene quasi-essentiality. (A) Dcm methylation profiles of non-coding regions in core and accessory genes of 84 *E. coli* strains, showing the proportions of methylation, unmethylated motif, and missing motifs. (B) For Dcm methylation in non-coding regions, the distributions of methylation per kilobase (MPK), methylation ratio (MR), and motif frequency ratio (MFR) are shown across essential genes and genes with varying prevalence. (C) Identification of high-methylation core genes based on Dcm methylation in non-coding regions. Genes in the top 10% for MPK, MR, and MFR were intersected with the core genes shared by 84 *E. coli* strains, resulting in 29 high-methylation core genes.

Supplemental Figure 14. Statistical comparison of methylation metrics across essential genes and genes of varying prevalence. (A–C) The color of each square indicates the –log10 *P* value, and the font color indicates whether the difference between groups is statistically significant (P < 0.05). Gene groups are as follows: (a) essential genes, (b) genes with prevalence = 100%, (c) prevalence 99–100%, (d) 90–99%, (e) 80–90%, (f) 70–80%, (g) 60–70%, and (h) < 60%. Group comparisons were performed using the one-sided Mann–Whitney U test. (A) Dcm methylation in gene regions; (B) Dam methylation in non-coding regions; (C) Dcm methylation in non-coding regions.

Supplemental Figure 15. Soft-threshold selection for WGCNA of gene regions based on mean connectivity. (A–B) For each panel: left, complete methylation; middle, sense methylation; right, antisense methylation. Red arrows denote the selected soft thresholds. (A) Dam methylation in gene regions. (B) Dcm methylation in gene regions.

Supplemental Figure 16. Soft-threshold selection for WGCNA of non-coding regions based on mean connectivity. (A–B) For each panel: left, complete methylation; middle, sense methylation; right, antisense methylation. Red arrows denote the selected soft thresholds. (A) Dam methylation in non-coding regions. (B) Dcm methylation in non-coding regions.

Supplemental Figure 17. Co-methylation network based on Dcm methylation in gene regions is associated with gene connectivity and biological processes. (A) TOM (Topological Overlap Matrix) of Dcm methylation in gene regions. (B) Comparison of TOM values between high-confidence and low-confidence gene pairs across seven *E. coli* strains in the STRING database, including CFT073, EDL933, IAI1, IAI39, MG1655, Sakai, and UMN026. Statistical significance was determined using a two-sided Mann–Whitney U test. (C) Consensus co-methylation network based on Dcm methylation in gene regions, constructed using complete, sense, and antisense methylation data. Modules containing at least 20 genes were identified. The gene names for each module are provided in Supplemental Table 2. (D) GO biological process annotations for each consensus co-methylation module. Functional enrichment was performed using the ClusterProfiler2 package and grouped under broader ontology terms using the "rrvgo" package. *P* < 0.05 is denoted by *, *P* < 0.01 by **, and *P* < 10^-4^ by ***.

Supplemental Figure 18. Co-methylation network based on Dam methylation in non-coding regions is associated with gene connectivity and biological processes. (A) TOM (Topological Overlap Matrix) of Dam methylation in non-coding regions. (B) Comparison of TOM values between high-confidence and low-confidence gene pairs across seven *E. coli* strains in the STRING database, including CFT073, EDL933, IAI1, IAI39, MG1655, and UMN026. Statistical significance was determined using a two-sided Mann–Whitney U test. (C) Consensus co-methylation network based on Dam methylation in non-coding regions, constructed using complete, sense, and antisense methylation data. Modules containing at least 20 genes were identified. The gene names for each module are provided in Supplemental Table 3. (D) GO biological process annotations for each consensus co-methylation module. Functional enrichment was performed using the ClusterProfiler2 package and grouped under broader ontology terms using the "rrvgo" package. *P* < 0.05 is denoted by *, *P* < 0.01 by **, and *P* < 10^-4^ by ***.

Supplemental Figure 19. Co-methylation network based on Dcm methylation in non-coding regions is associated with gene connectivity and biological processes. (A) TOM (Topological Overlap Matrix) of Dcm methylation in non-coding regions. (B) Comparison of TOM values between high-confidence and low-confidence gene pairs across seven *E. coli* strains in the STRING database, including CFT073, EDL933, IAI1, IAI39, MG1655, and UMN026. Statistical significance was determined using a two-sided Mann–Whitney U test. (C) Consensus co-methylation network based on Dcm methylation in non-coding regions, constructed using complete, sense, and antisense methylation data. Modules containing at least 20 genes were identified. The gene names for each module are provided in Supplemental Table 4. (D) GO biological process annotations for each consensus co-methylation module. Functional enrichment was performed using the ClusterProfiler2 package and grouped under broader ontology terms using the "rrvgo" package. *P* < 0.05 is denoted by *, *P* < 0.01 by **, and *P* < 10^-4^ by ***.

Supplemental Figure 20. Virulence gene annotation of 84 *E. coli* strains. Virulence genes were annotated for 84 *E. coli* strains based on the VFDB (Virulence Factors Database). A heatmap displays the presence or absence of virulence genes, with strains ordered according to the phylogenetic tree.

Supplemental Figure 21. Classification of methylation types. Methylation types are categorized as sense or antisense based on the direction of transcription.
