# Supplementary material for: A Pan‐Methylome Framework for Population‐Scale Bacterial Epigenomics
Source: Adv Sci (Weinh). 2026 Jul 13:e76559. Online ahead of print. doi: 10.1002/advs.76559 (PMC13360123; doi:10.1002/advs.76559)
Supplement: Supplementary file 3 — Supporting File 3: advs76559‐sup‐0003‐SuppMatTablesS1‐S11.zip. [file ADVS-9999-e76559-s002.zip › TableS9.docx]

**Supplemental Table 9. CRISPRi primer design.**

| Primer | Sequence |
| --- | --- |
| pgRNA-F | TTAACAGGGAAGTGAGAGGG |
| pgRNA-R | CTCAATGATGATGATGATGATGG |
| atpG-sgRNA-F | TCGACCGACCGTGGTTTGTGCGGGTTTTAGAGCTAGAAATAGCAA |
| atpG-sgRNA-R | CCGCACAAACCACGGTCGGTCGAACTAGTATTATACCTAGGACTG |
| chbA-sgRNA-F | AGCTGGAAGAAGTGGTGATGGGGGTTTTAGAGCTAGAAATAGCAA |
| chbA-sgRNA-R | CCCCATCACCACTTCTTCCAGCTACTAGTATTATACCTAGGACTG |
| citG-sgRNA-F | CTCGTGGATCGCATTAACTGCGGGTTTTAGAGCTAGAAATAGCAA |
| citG-sgRNA-R | CCGCAGTTAATGCGATCCACGAGACTAGTATTATACCTAGGACTG |
| cysN-sgRNA-F | GATCAACAAAATGGATCTGGTGGGTTTTAGAGCTAGAAATAGCAA |
| cysN-sgRNA-R | CCACCAGATCCATTTTGTTGATCACTAGTATTATACCTAGGACTG |
| dcuB-sgRNA-F | TTCCGGTATGATCGCCATCGTGGGTTTTAGAGCTAGAAATAGCAA |
| dcuB-sgRNA-R | CCACGATGGCGATCATACCGGAAACTAGTATTATACCTAGGACTG |
| dnaC-sgRNA-F | AACCGGCAAAAACCATCTGGCGGGTTTTAGAGCTAGAAATAGCAA |
| dnaC-sgRNA-R | CCGCCAGATGGTTTTTGCCGGTTACTAGTATTATACCTAGGACTG |
| glnQ-sgRNA-F | GGCTGAAGAAGGGATGACGATGGGTTTTAGAGCTAGAAATAGCAA |
| glnQ-sgRNA-R | CCATCGTCATCCCTTCTTCAGCCACTAGTATTATACCTAGGACTG |
| grxD-sgRNA-F | GTGATCGAAATGTATCAGCGTGGGTTTTAGAGCTAGAAATAGCAA |
| grxD-sgRNA-R | CCACGCTGATACATTTCGATCACACTAGTATTATACCTAGGACTG |
| gyrA-sgRNA-F | GAACCTGAAAGACATCATCGCGGGTTTTAGAGCTAGAAATAGCAA |
| gyrA-sgRNA-R | CCGCGATGATGTCTTTCAGGTTCACTAGTATTATACCTAGGACTG |
| lepA-sgRNA-F | AGTCGGCGATACCTTAACGCTGGGTTTTAGAGCTAGAAATAGCAA |
| lepA-sgRNA-R | CCAGCGTTAAGGTATCGCCGACTACTAGTATTATACCTAGGACTG |
| lptE-sgRNA-F | CATCGCGAAAGATACCGCATCGGGTTTTAGAGCTAGAAATAGCAA |
| lptE-sgRNA-R | CCGATGCGGTATCTTTCGCGATGACTAGTATTATACCTAGGACTG |
| maeB-sgRNA-F | GATTGCAGAACTCGCCCATGCGGGTTTTAGAGCTAGAAATAGCAA |
| maeB-sgRNA-R | CCGCATGGGCGAGTTCTGCAATCACTAGTATTATACCTAGGACTG |
| mlaB-sgRNA-F | CAACAATGTGACGCTTCAGGGGGGTTTTAGAGCTAGAAATAGCAA |
| mlaB-sgRNA-R | CCCCCTGAAGCGTCACATTGTTGACTAGTATTATACCTAGGACTG |
| rplP-sgRNA-F | GGGTAAAGGTAAAGGTAACGTGGGTTTTAGAGCTAGAAATAGCAA |
| rplP-sgRNA-R | CCACGTTACCTTTACCTTTACCCACTAGTATTATACCTAGGACTG |
| ybaK-sgRNA-F | TGATATGAAACACCTTGCCGTGGGTTTTAGAGCTAGAAATAGCAA |
| ybaK-sgRNA-R | CCACGGCAAGGTGTTTCATATCAACTAGTATTATACCTAGGACTG |
| yddW-sgRNA-F | CAATCGTCACAACCGATGCGTGGGTTTTAGAGCTAGAAATAGCAA |
| yddW-sgRNA-R | CCACGCATCGGTTGTGACGATTGACTAGTATTATACCTAGGACTG |
| yehS-sgRNA-F | GCTACGTAAAGAAGACGAAGAGGGTTTTAGAGCTAGAAATAGCAA |
| yehS-sgRNA-R | CCTCTTCGTCTTCTTTACGTAGCACTAGTATTATACCTAGGACTG |
| yhhJ-sgRNA-F | TGGCGAAGATCTGGTCGATGGGGGTTTTAGAGCTAGAAATAGCAA |
| yhhJ-sgRNA-R | CCCCATCGACCAGATCTTCGCCAACTAGTATTATACCTAGGACTG |
| yhhW-sgRNA-F | GTTGAGCATCAGGACAGCATGGGGTTTTAGAGCTAGAAATAGCAA |
| yhhW-sgRNA-R | CCCATGCTGTCCTGATGCTCAACACTAGTATTATACCTAGGACTG |
| yjbD-sgRNA-F | TCAGCAAATACGTCGACGCGCGGGTTTTAGAGCTAGAAATAGCAA |
| yjbD-sgRNA-R | CCGCGCGTCGACGTATTTGCTGAACTAGTATTATACCTAGGACTG |
