# Supplementary material for: A Pan‐Methylome Framework for Population‐Scale Bacterial Epigenomics
Source: Adv Sci (Weinh). 2026 Jul 13:e76559. Online ahead of print. doi: 10.1002/advs.76559 (PMC13360123; doi:10.1002/advs.76559)
Supplement: Supplementary file 3 — Supporting File 3: advs76559‐sup‐0003‐SuppMatTablesS1‐S11.zip. [file ADVS-9999-e76559-s002.zip › TableS10.docx]

**Supplemental Table 10. DNA fragments designed by synonymous codon shuffling.**

| Gene | Sequence |
| --- | --- |
| HAIA13 | ATGCAGAATGCTGGTTCTTTAGTCGTCTTAGGTTCTATCAACGCCGACCATATCTTAAACTTACAGTCCTTCCCCACCCCGGGTGAGACTGTTACTGGCAATCATTACCAAGTCGCTTTCGGTGGTAAGGGTGCTAACCAAGCCGTTGCCGCCGGTCGCTCTGGCGCTAACATTGCTTTCATCGCTTGCACTGGCGACGATTCTATCGGCGAATCTGTCCGTCAACAATTAGCTACCGACAATATCGACATCACCCCTGTTTCTGTGATCAAGGGTGAGTCTACTGGCGTTGCTTTAATCTTCGTCAACGGTGAGGGCGAAAACGTTATTGGCATCCACGCTGGTGCCAACGCCGCTTTATCTCCTGCTTTAGTTGAGGCTCAGCGCGAACGCATCGCTAATGCTTCTGCTTTGTTGATGCAATTAGAGTCTCCTTTAGAGTCTGTTATGGCTGCTGCTAAGATTGCTCACCAGAACAAAACCATTGTCGCTTTAAATCCTGCCCCTGCCCGTGAGTTACCCGATGAGTTATTAGCTTTAGTTGATATCATCACTCCTAATGAGACTGAGGCTGAGAAATTAACTGGCATCCGCGTCGAGAACGACGAGGACGCTGCTAAAGCTGCTCAAGTTTTACACGAGAAGGGCATTCGCACCGTTTTAATCACCTTGGGTTCTCGCGGCGTTTGGGCTAGTGTTAACGGCGAGGGCCAACGTGTCCCTGGTTTTCGTGTTCAAGCCGTTGACACTATCGCCGCTGGTGACACTTTCAATGGTGCTTTGATTACTGCTTTATTAGAGGAGAAGCCTTTACCTGAAGCTATCCGTTTCGCTCACGCCGCTGCCGCTATCGCTGTTACTCGCAAGGGTGCTCAGCCCTCTGTTCCTTGGCGCGAGGAGATCGATGCTTTCTTGGACCGTCAACGTTAA |
